# Supplementary material for: IMPDH inhibition activates TLR‐VCAM1 pathway and suppresses the development of MLL‐fusion leukemia
Source: EMBO Mol Med. 2022 Dec 1;15(1):e15631. doi: 10.15252/emmm.202115631 (PMC9832838; doi:10.15252/emmm.202115631)
Supplement: Supplementary file 1 — Appendix [file EMMM-15-e15631-s001.pdf]

IMPDH inhibition activates TLR-VCAM1 pathway and suppresses the development of MLL-fusion leukemia

Xiaoxiao Liu, et al.

* Correspondence: goyama@edu.k.u-tokyo.ac.jp

This PDF file includes 4 Appendix tables and 10 Appendix Figures together with legends:

- Appendix Table S1: Information of PDX samples
- Appendix Table S2: Antibodies used for Western Blotting
- Appendix Table S3: Antibodies used for Flow Cytometry
- Appendix Table S4: Antibodies used for Single Cell Mass Cytometry
- Appendix Figures S1: Guanosine supplementation reversed the effect of IMPDH inhibitors on human myeloid leukemia cells in vitro
- Appendix Figures S2: MPA induced cell cycle arrest, apoptosis of MOLM13 cells
- Appendix Figures S3: Combination effects of MPA with Decitabine or with Venetoclax on MLL-AF9 cells
- Appendix Figures S4: The effect of IMPDH inhibitors on human lymphoid leukemia cells in vitro
- Appendix Figures S5: IMPDH inhibitors show less efficiency in PDX-derived leukemia in immunodeficiency mice
- Appendix Figures S6: No substantial effects of the alternate-day administration of FF-10501-01 on the numbers of immune cells in mice
- Appendix Figures S7: Reduced purine and pyrimidine levels in FF-10501-01 treated MLL-AF9 cells
- Appendix Figures S8: Altered levels of bases and amino acids in FF-10501-01 treated MLL-AF9 cells
- Appendix Figures S9: Single-cell Mass Cytometry's gating strategy and results
- Appendix Figures S10: Vcam1 is dispensable for FF-10501-01-induced myeloid differentiation of MLL-AF9 cells

Appendix Table S1. Information of PDX samples

Sample No.	PDX ID	Disease Stage	Genetic abnormalities	Lineage
AML#1	2017-14	R/R	tAML, MLL/AF10	AML
AML#2	2016-7	R/R	MLL/AF9	AML
AML#3	2013-11	R/R	MLL/SEPT6	AML
AML#4	2017-94	R/R	Complex Karyotype	AML
AML#5	2017-63	R/R	Mnx1/ETV6 PTPN11	AML
AML#6	2016-97	R/R	CBFA2T3-GLIS2	AML
AML#7	2017-38	R/R	MLL/AF9 with Nras mutation	AML
ALL#1	2019-116		t (4;11) -- MLL/AF4	ALL
ALL#2	2018-190	De novo	Infant MLL/MA9	ALL

Appendix Table S2. Antibodies used for Western Blotting

Protein detected	Catalog number	Supplier	Dilution ratio
p53 (DO-1)	sc-126	Santa Cruz	1/1000
p53 (1C12)	2524	Cell Signaling Technology	1/1000
Bax	2774	Cell Signaling Technology	1/1000
BCL2 (D55G8)	4223	Cell Signaling Technology	1/1000
p21 Waf1/Cip1 (12D1)	2947	Cell Signaling Technology	1/1000
IMPDH2 (E2Q1Z)	57068	Cell Signaling Technology	1/1000
IMPDH1 (E5Z5F)	35914	Cell Signaling Technology	1/1000
phospho-IKb (Ser32/36)	9246	Cell Signaling Technology	1/1000
Ikb (L35A5)	4814	Cell Signaling Technology	1/1000
phospho-p38 (Thr180/Tyr182) (28B10)	9216	Cell Signaling Technology	1/1000
p38 MAPK	9212	Cell Signaling Technology	1/1000
GAPDH (D16H11) XP	5174	Cell Signaling Technology	1/2000
Tubulin (D-66)	T0198	Sigma-Aldrich	1/2000

Appendix Table S3. Antibodies used for Flow Cytometry

Antibody Name	Fluorescence	Clone	Catalog Number	Supplier	Dilution ratio
anti-mouse-Gr-1 (Ly6G/6C)	PE	RB-8C6	108408	Biolegend	1/400
anti-mouse-CD117 (c-Kit)	APC	2B8	17-1171-83	eBioscience	1/400
anti-mouse/human-CD11b	PE-Cy7	M1/70	101216	Biolegend	1/400
Annexin V	APC		640920	Biolegend	1/100
Vybrant DyeCycle	violet		V35003	Invitrogen	1/250
anti-human-CD11b	PE	ICRF44	301306	Biolegend	1/400
anti-human-CD14	APC	M5E2	301808	Biolegend	1/400
anti-human-CD66b	PE-Cy7	G10F5	305115	Biolegend	1/400
anti-human-CD115	APC	9-4D2-1E4	347306	Biolegend	1/400
anti-human-CD45	FITC	HI30(RUO)	555482	BD Bioscience	1/200
anti-human-CD19	PE-Cy7	HIB19	302215	Biolegend	1/400
anti-human-CD10	PE	HI10a	312204	Biolegend	1/400
anti-human-CD34	APC	561	343608	Biolegend	1/200
anti-mouse-NK1.1	PE	PK136	12-5941-83	eBioscience	1/400
anti-mouse-CD3	eFluor 450	17A2	48-0032-82	eBioscience	1/400
anti-mouse-CD8a	PE	53-6.7	100707	Biolegend	1/500
anti-mouse-CD4	BV421	GK1.5	100438	Biolegend	1/400
anti-mouse-CD19	PE-Cy7	6D5	115520	Biolegend	1/400

anti-mouse/human-B220	APC	RA3-6B2	10-0452-81	eBioscience	1/400
anti-mouse-CD115	PE	AFS98	135505	Biolegend	1/400
anti-mouse-Gr1	biotin	RB6-8C5	108404	Biolegend	1/400
streptavidin	BV421		405226	Biolegend	1/200
anti-mouse-F4/80	APC	BM8	123115	Biolegend	1/400
anti-mouse-CD106 (VCAM-1)	Alexa Fluor 647	429 (MVCAM.A)	105712	Biolegend	1/400
anti-mouse-CD98 (4F2)	PE	RL388	128207	Biolegend	1/400
anti-human-CD98	PE	UM7F8	556077	BD Biosciences	1/400

Appendix Table S4. Antibodies used for Single Cell Mass Cytometry

Metal Tag	Antigen	Clone	Vendor	Cat No.	Dilution ratio
141Pr	Ly-6G/C(Gr1)	RB6-8C5	Fluidigm	3141005B	1/100
143Nd	CD41	MWreg30	Fluidigm	3143009B	1/100
145Nd	CD4	RM4-5	Fluidigm	3145002B	1/100
146Nd	*VCAM1	MVCAM.A(429)	Bio Legend	105702	1/100
147Sm	CD45.2	104	Fluidigm	3147004B	1/100
149Sm	*CXCR4(CD184)	L276F12	Bio legend	146502	1/100
150Nd	*HIF1α	H206	Santa Claus	sc-10790	1/100
151Eu	CD49d (Integrin alpha 4)	R1-2	Fluidigm	3151016B	1/100
152Sm	phospho-Akt [S473]	D9E	Fluidigm	3152005A	1/100
153Eu	phospho-STAT1 [Y701]	58D6	Fluidigm	3153003A	1/100
156Gd	phospho-p38 MAP Kinase [T180/Y182]	D3F9	Fluidigm	3156002A	1/100
158Gd	phospho-STAT3 [Y705]	4/P-STAT3	Fluidigm	3158005A	1/100
159Tb	phospho-MAPKAP Kinase2 [T334]	27B7	Fluidigm	3159010A	1/100
160Gd	CD45R (B220)	RA3-6B2	Fluidigm	3160012B	1/100
161Dy	Ki-67	B56	Fluidigm	3161007B	1/100
162Dy	*p53 (For whole protein)	X77	Abcam	ab16465	1/100
163Dy	*phospho-mTOR [S2448]	EPR426(2)	Abcam	ab109268	1/100

165Ho	beta-Catenin	D13A1	Fluidigm	3165027A	1/100
167Er	*EPCR（CD201)	eBio 1560	eBioscience	16-2012-83	1/100
168Er	CD8a	53-6.7	Fluidigm	3168003B	1/100
169Tm	Ly-6A/E (Sca-1)	D7	Fluidigm	3169015B	1/100
170Er	CD49b	HMa2	Fluidigm	3170008B	1/100
171Yb	phospho-ERK 1/2 [T202/Y204]	D13.14.4E	Fluidigm	3171010A	1/100
172Yb	CD11b (Mac-1)	M1/70	Fluidigm	3172012B	1/100
173Yb	CD117 (c-kit)	2B8	Fluidigm	3173004B	1/100
174Yb	phospho-STAT4	38/p-Stat4	Fluidigm	3174005A	1/100
175Lu	*PGC1 alpha	polyclonal	Abcam	ab54481	1/100
191Ir	Cell-ID^{TM} Intercalator–Ir (DNA)	NA	Fluidigm	201192A	1/100
193Ir	Cell-ID^{TM} Intercalator–Ir (DNA)	NA	Fluidigm	201192A	1/100
198Pt	Cell-ID^{TM} Cisplatin (Live/Dead)	NA	Fluidigm	201198	1/100

NA: not available

Appendix Figure legends

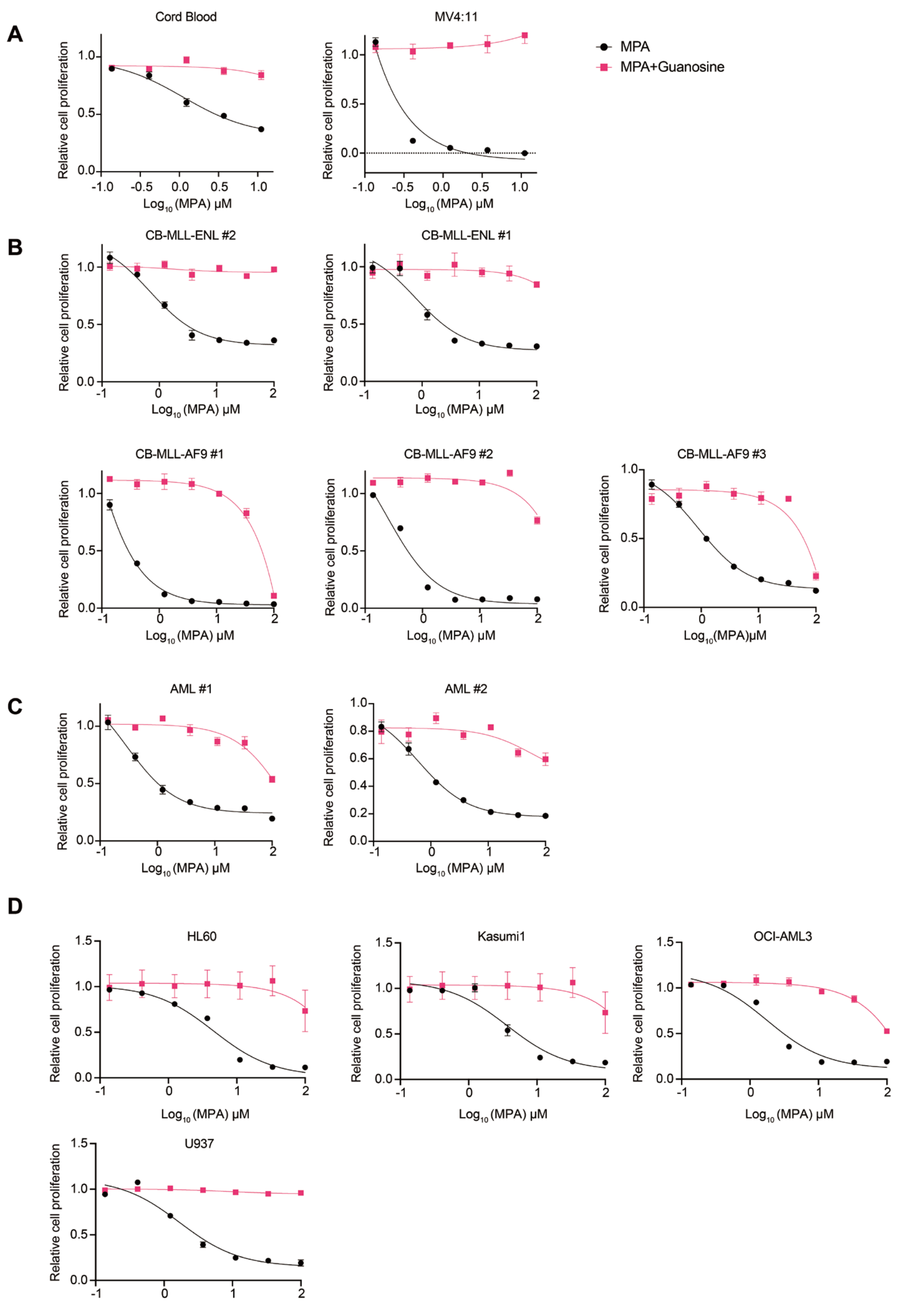

Appendix Figure S1. Guanosine supplementation reversed the effect of IMPDH inhibitors on human myeloid leukemia cells *in vitro*
Cell viability assays were performed using WST-8 or WST-1. Human CB CD34^{+} cells and MV4; 11 cells (**A**), CB cells expressing MLL-ENL (CB-MLL-ENL-#1, 2) or MLL-AF9 (CB-MLL-AF9#1, 2, 3) (**B**) and patient cells (AML#1 and AML#2) , (**C**) Human myeloid leukemia cell lines (HL60, Kasumi-1, OCI-AML3, U937) **(D)** were treated with titrating doses of MPA (1-100 mM) for 72 hours with/without 100 μM Guanosine in triplicates.

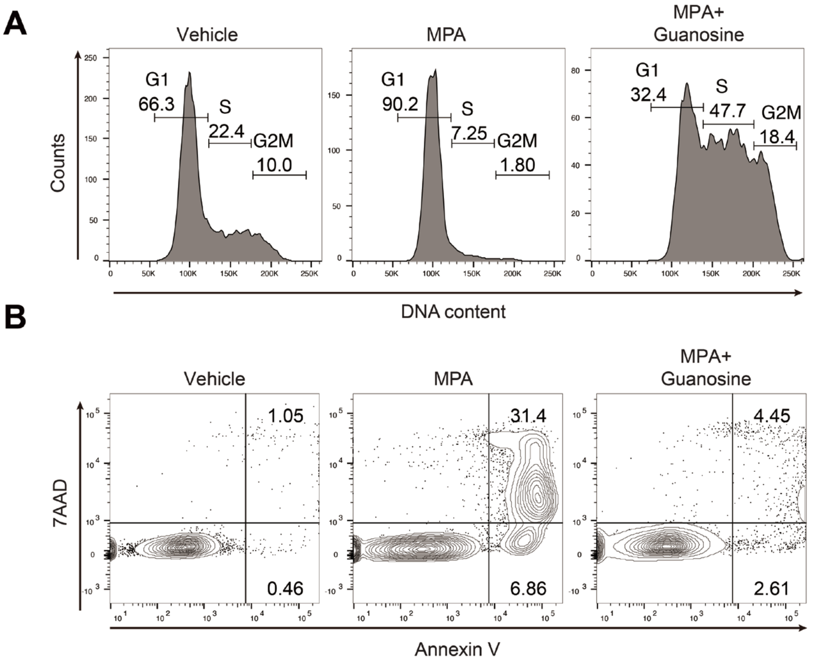

Appendix Figure S2. MPA induced cell cycle arrest, apoptosis of MOLM13 cells

FCM plots showing the cell cycle status (**A**), apoptosis (**B**) in MOLM13 cells treated with/without 1 mM MPA and 100 mM Guanosine for 36 hours. The number shows in (**A**) indicate the frequency of G1, S, G2M phase of the cells and the number shows in (**B**) indicate the frequency of apoptotic cells.

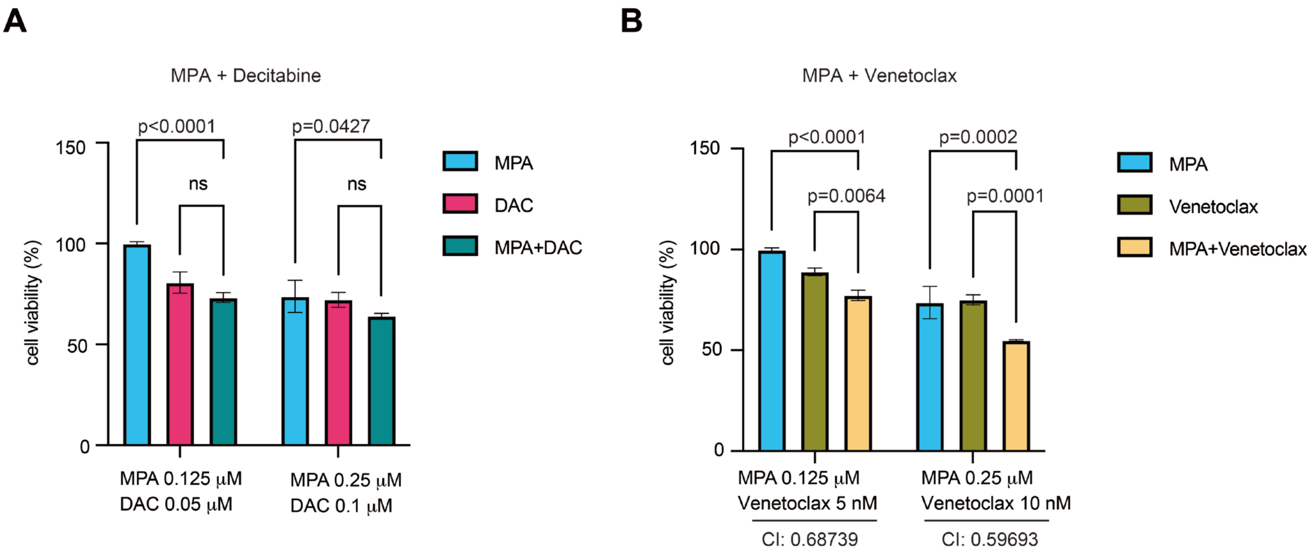

Appendix Figure S3. Combination effects of MPA with Decitabine or with Venetoclax on MLL-AF9 cells

Cell viability assays were performed using WST-8. MLL-AF9-expressing CB cells were treated with the indicated doses of MPA (0.125 or 0.25 mM) together with (**A**) decitabine (0.05 or 0.1 mM) or with (**B**) Venetoclax (5 or 10 nM) for 72 hours in triplicates. Combination index (CI) was calculated using COMPUSYN (https://www.combosyn.com/).

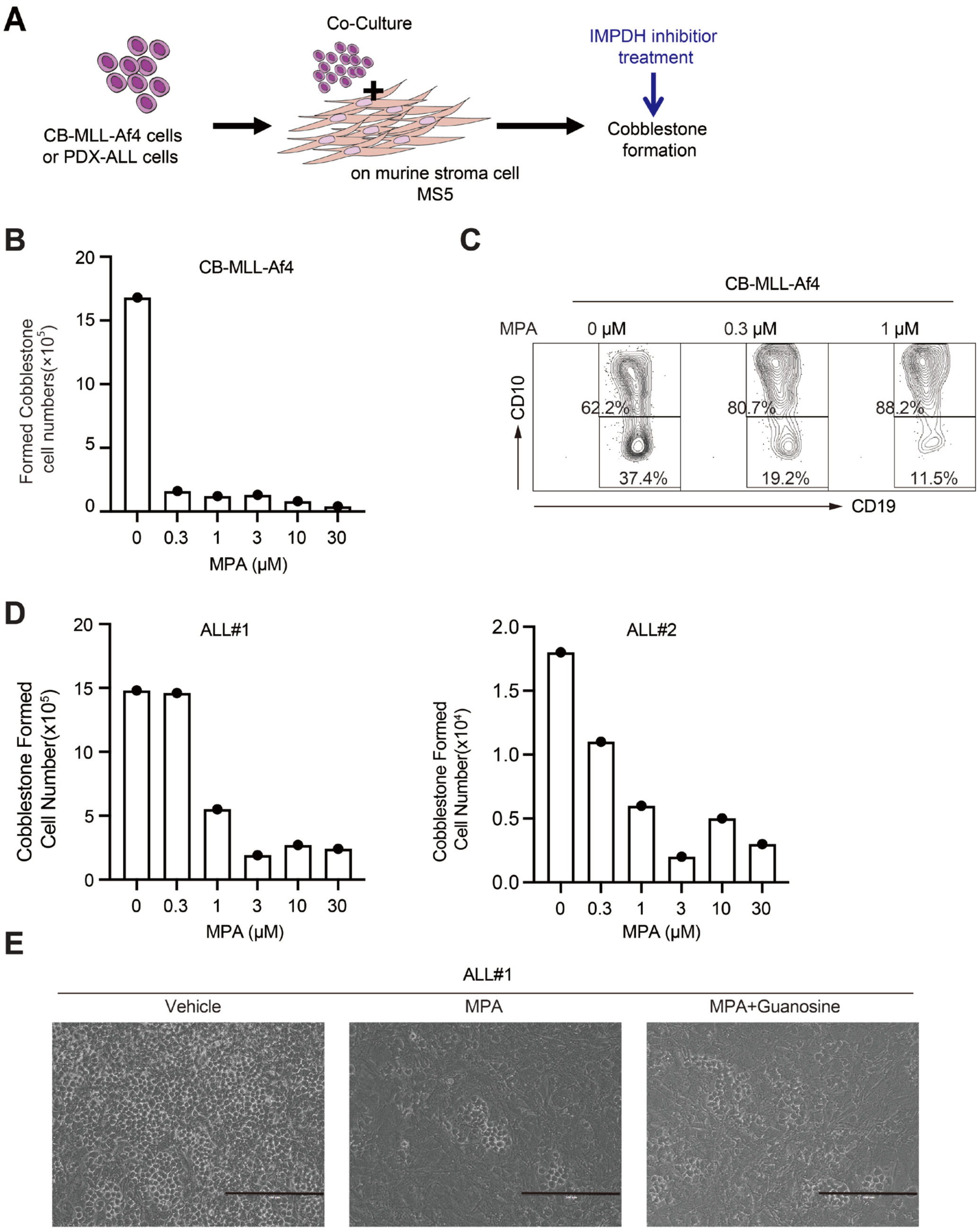

Appendix Figure S4. The effect of IMPDH inhibitors on human lymphoid leukemia cells *in vitro*

(A) Experimental Scheme used in (**B**)-(**E**). MLL-Af4-expressing CB (CB-MLL-Af4) cells or patient-derived ALL cells were co-cultured with murine stromal cell line MS-5. MPA was added at the indicated concentration to the coculture. The numbers of cobblestone forming cells were counted after 6 days culture. **(B)** Counts of cobblestone forming CB-MLL-Af4 cells are shown. **(C)** FCM plots of CD10 and CD19 expression in cobblestone formed MLL-Af4 cells with MPA at indicated concentrations after one-week culture. (**D**) Counts of cobblestone forming patient derived ALL cells ALL#1 (left) or ALL#2 (right) were shown. (**E**) Representative pictures of cobblestone forming cells treated with vehicle, 1 µM MPA with/without 100 µM guanosine are shown. Scale bar is 200 mm

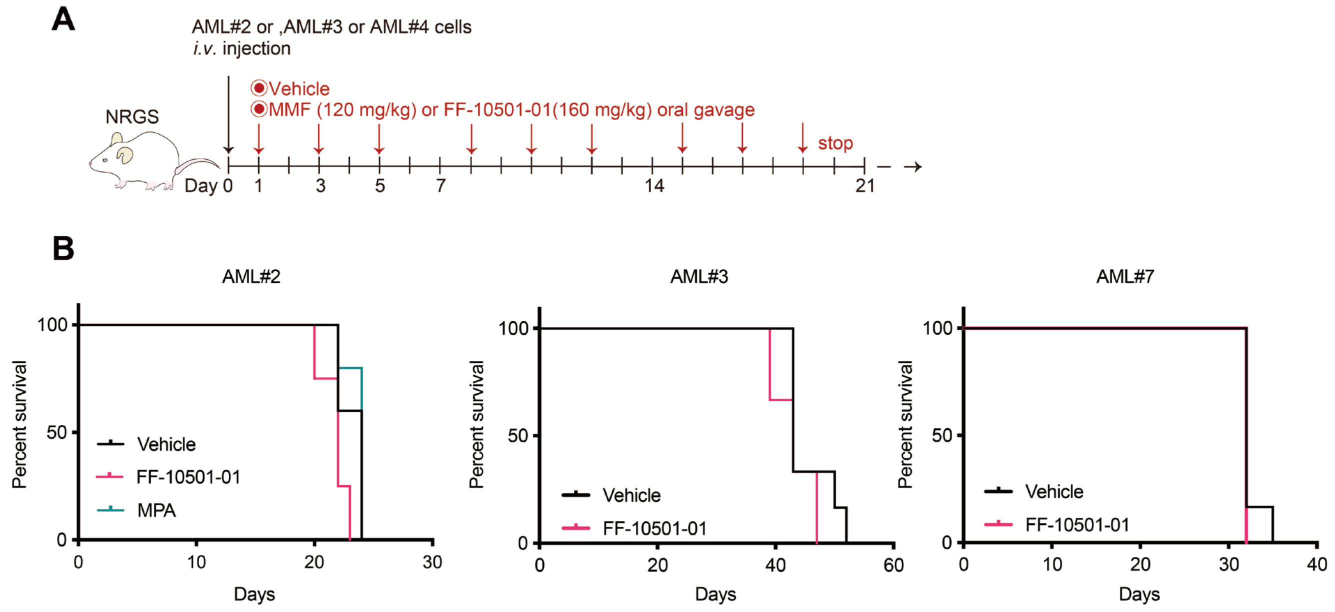

Appendix Figure S5. IMPDH inhibitors show less efficiency in PDX-derived leukemia in immunodeficiency mice.
(**A**) Experimental scheme used in (**B**). MLL-fusion PDX-derived cells (AML#2, AML#3, AML#7, and see Appendix Table S1) were transplanted to NRGS mice and treated with vehicle, MMF or FF-10501-01 every other day for three weeks. (**B**) Kaplan-Meier survival curves were shown. For AML#2, vehicle: n=5, FF: n=4, MMF: n=5. For AML#3 and AML#7, n=6 per group.

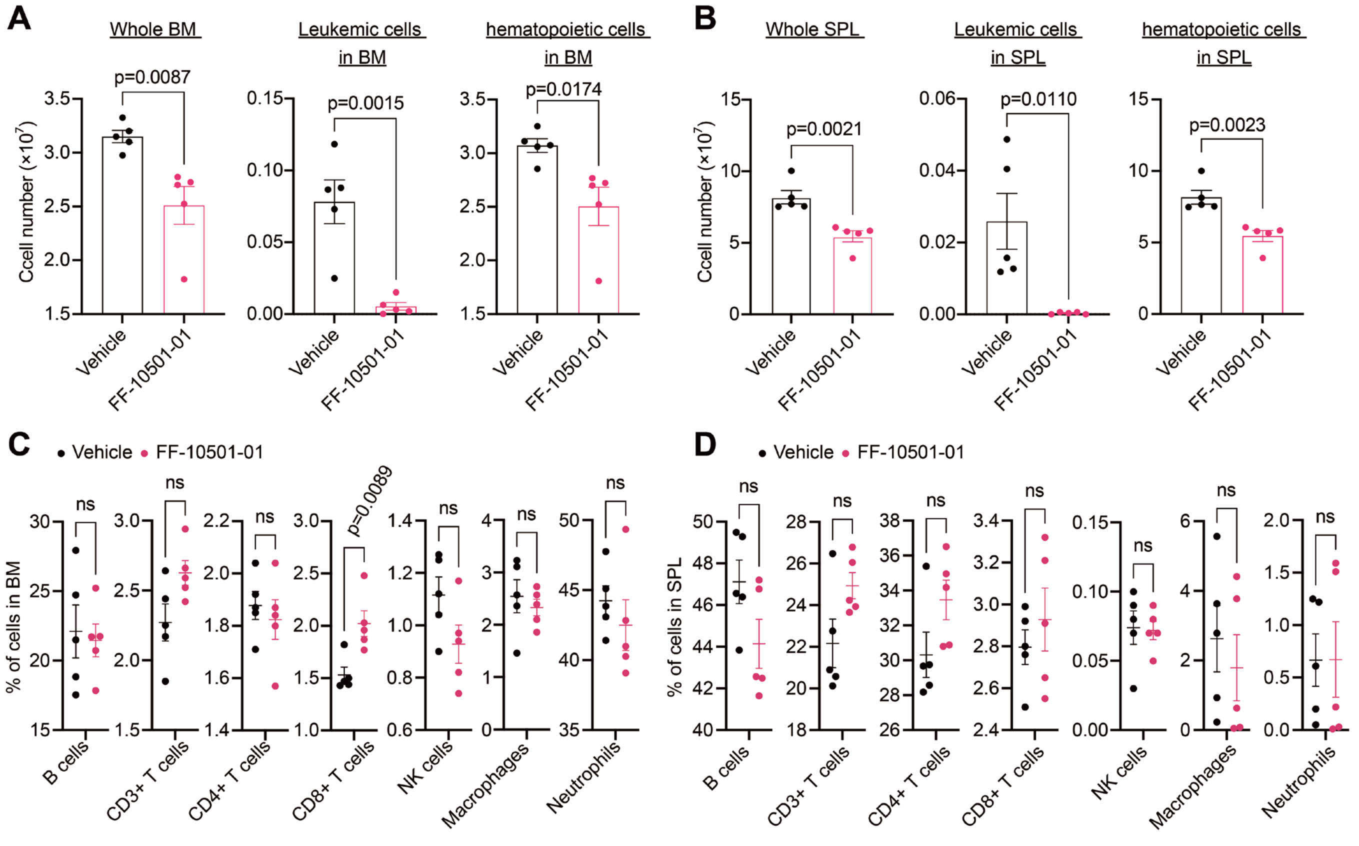

Appendix Figure S6. No substantial effects of the alternate-day administration of FF-10501-01 on the numbers of immune cells in mice
(**A** and **B**) C57BL/6J mice were transplanted with MLL-AF9-GFP cells and were then treated with vehicle (n=5) or FF-10501-01 (n=5) every other day. Bone marrow and spleen cells were collected and analyzed on day 10. The numbers of whole bone marrow cells, GFP^{+} leukemic cells, and GFP^{-} recipient-derived hematopoietic cells in bone marrow (**A**) and spleen (**B**), and the frequencies of immune cells (B220^{+}CD19^{+} B cells, CD3^{+}, CD4^{+}, CD8^{+} T cells, CD3+NK1.1^{+} NK cells, CD11b^{+}/CD11b^{low}Gr1^{-}F4/80^{+} macrophages, Gr1^{high}CD11b^{+} neutrophils) in bone marrow (**C**) and spleen (**D**) are shown. A two-tailed unpaired t-test was used for the comparison.

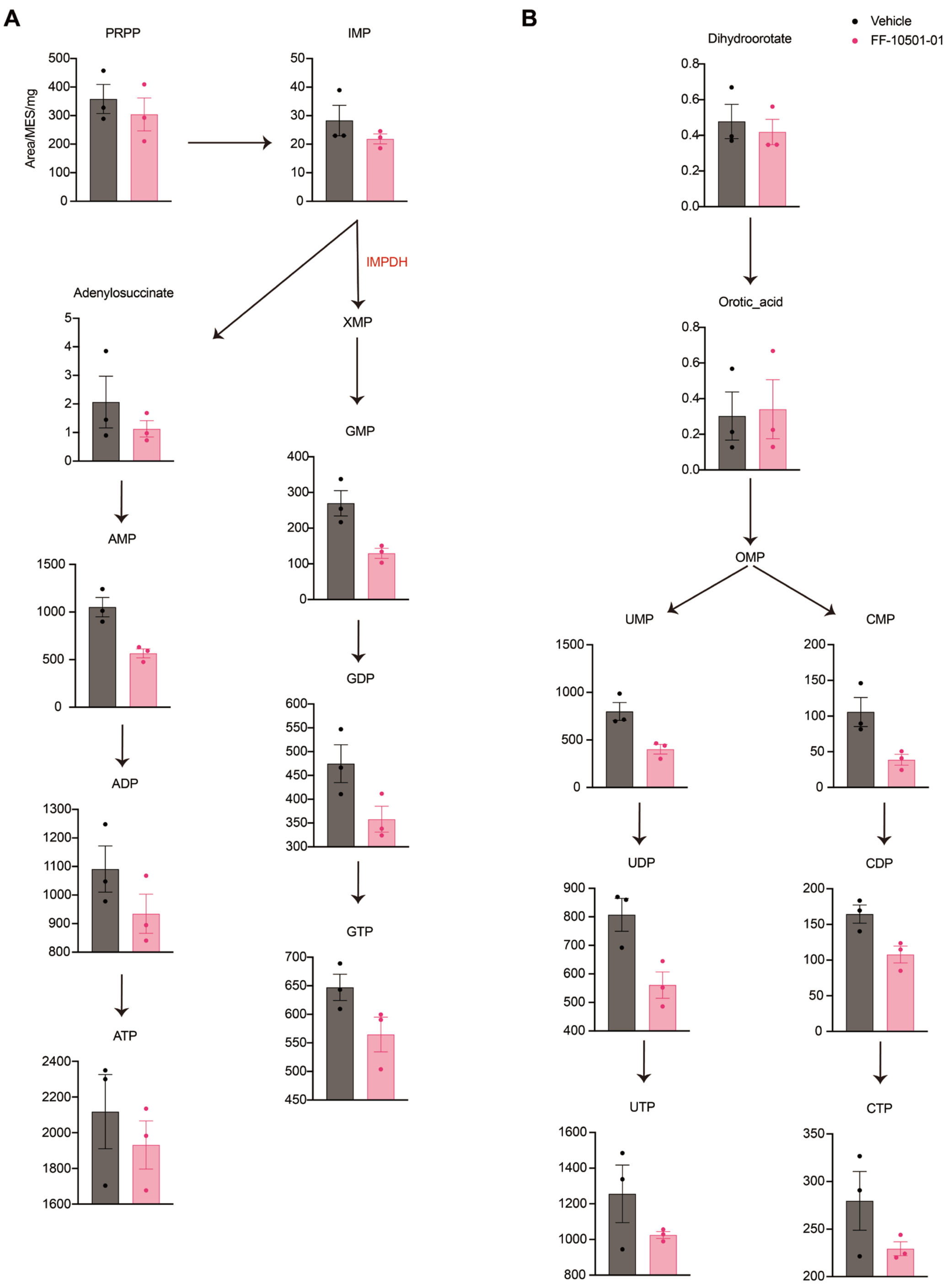

Appendix Figure S7. Reduced purine and pyrimidine levels in FF-10501-01 treated MLL-AF9 cells

C57BL/6J mice were transplanted with MLL-AF9-GFP cells and were treated with vehicle or FF-10501-01 on day 11 and day 12. GFP$^+$ MLL-AF9 cells were collected from vehicle- or FF-10501-01-treated mice and were used for metabolome analysis on day 13. Levels of the molecules in purine (**A**) and pyrimidine (**B**) synthesis pathway were assessed using LC-MS analysis. Black: Vehicle-, Pink: FF-10501-01-treated MLL-AF9 cells.

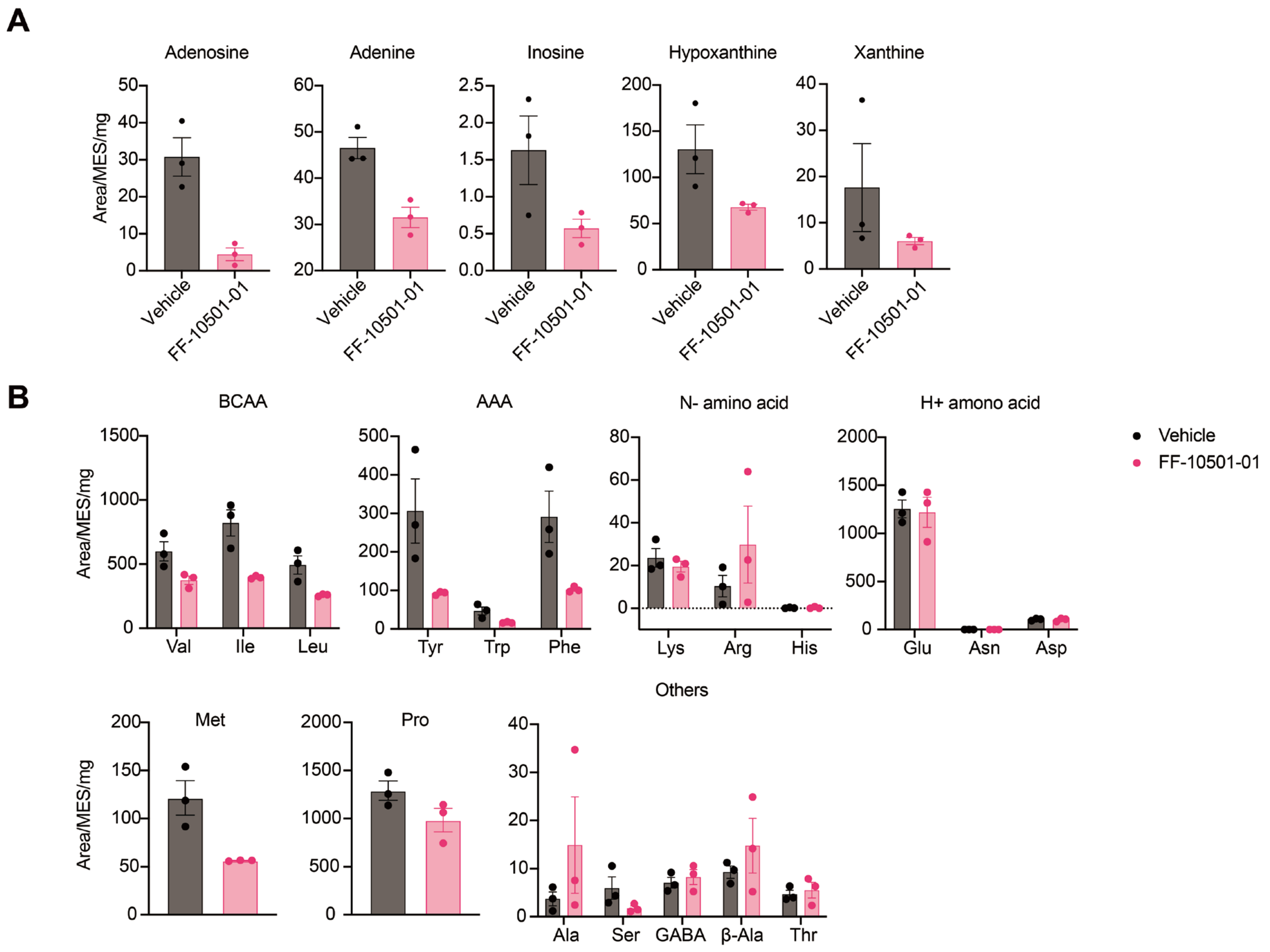

Appendix Figure S8. Altered levels of bases and amino acids in FF-10501-01 treated MLL-AF9 cells.
(**A**) Levels of adenosine, adenine, inosine, hypoxanthine, xanthine in Vehicle (Black) or FF-10501-01 (Pink) treated MLL-AF9 cells. The y-axis is calculated by area/MES/mg. (**B**) Amino acid levels in Vehicle (Black) or FF-10501-01 (Pink) treated MLL-AF9 cells. The y-axis is calculated by area/MES/mg.

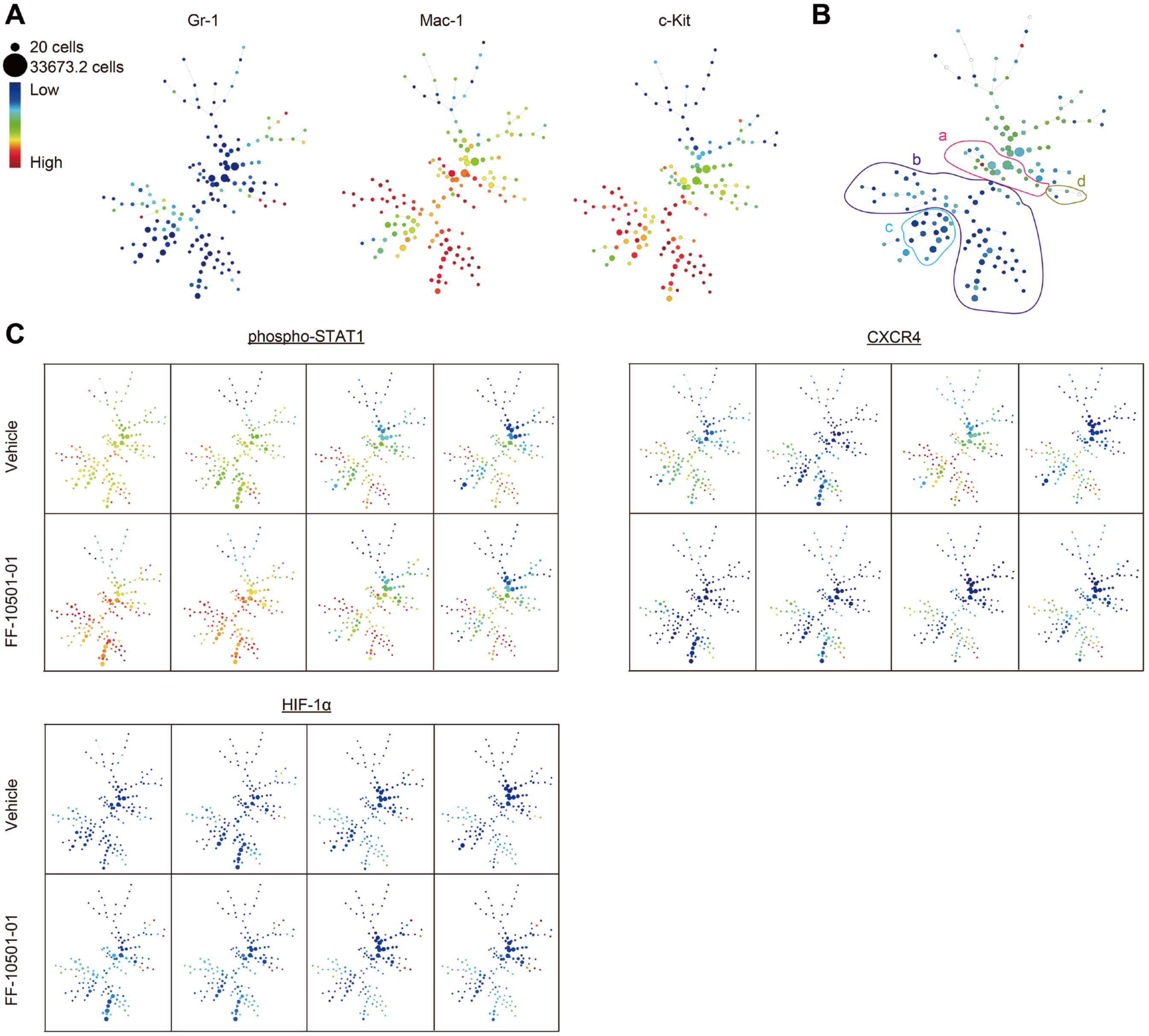

Appendix Figure S9. Single-cell Mass Cytometry's gating strategy and results

CD45.2$^+$ MLL-AF9 cells were collected from CD45.1$^+$ recipient mice treated with vehicle (n=4) or FF-10501-01 (n=4) and were subjected to mass cytometry analysis. CD45.2$^+$ fraction was used for SPADE (Spanning-tree Progression Analysis of Density-normalized Events) analysis in Cytobank (https://premium.cytobank.org/cytobank/login). (**A**) Gr-1$^+$, Mac-1$^+$, and c-Kit$^+$ fractions were shown on the SPADE tree. (**B**) Gating strategy on SPADE tree using Gr-1, Mac-1 and c-Kit expression. Four populations were defined (related to Figure 6B). (**C**) Expression of phospho-STAT1, CXCR4, and HIF-1α in each mouse was shown. The SPADE trees show each marker's median expression.

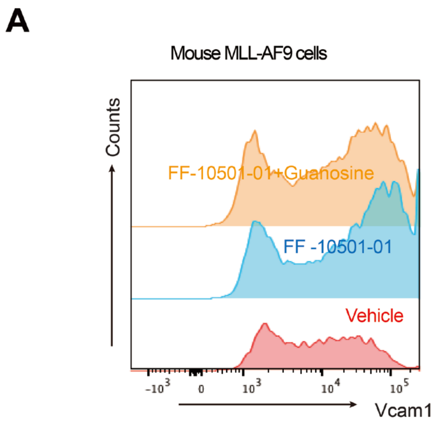

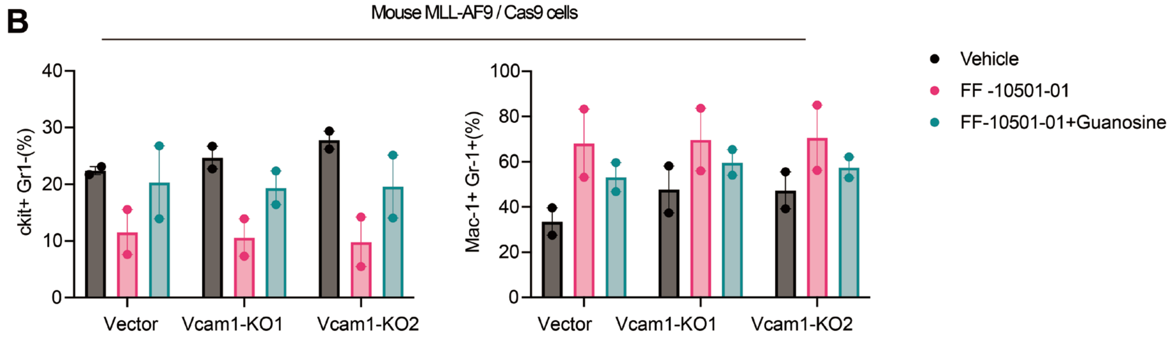

Appendix Figure S10. Vcam1 is dispensable for FF-10501-01-induced myeloid differentiation of MLL-AF9 cells

(**A**) Mouse MLL-AF9 cells were treated with/ without 5 mM FF-10501-01 and 10 mM guanosine in cytokine containing suspension medium for 48 hours. Vcam1 expression was evaluated by FCM. (**B**) Mouse MLL-AF9-Cas9 transduced with vector or two independent Vcam1-targeting sgRNAs (KO1 and KO2) were with/without 0.5 mM FF-10501-01 and 10 mM guanosine in cytokine containing suspension medium for 72 hours. Frequency of c-Kit^{+} Gr-1^{-} and Mac-1^{+} Gr-1^{+} in vehicle, FF-1050-01 or FF-10501-01+ guanosine treated cells with/without Vcma1-depeletion. For each group, n=2.
