# Supplementary material for: IMPDH inhibition activates TLR‐VCAM1 pathway and suppresses the development of MLL‐fusion leukemia
Source: EMBO Mol Med. 2022 Dec 1;15(1):e15631. doi: 10.15252/emmm.202115631 (PMC9832838; doi:10.15252/emmm.202115631)
Supplement: Supplementary file 2 — Expanded View Figures PDF [file EMMM-15-e15631-s005.pdf]

Expanded View Figures

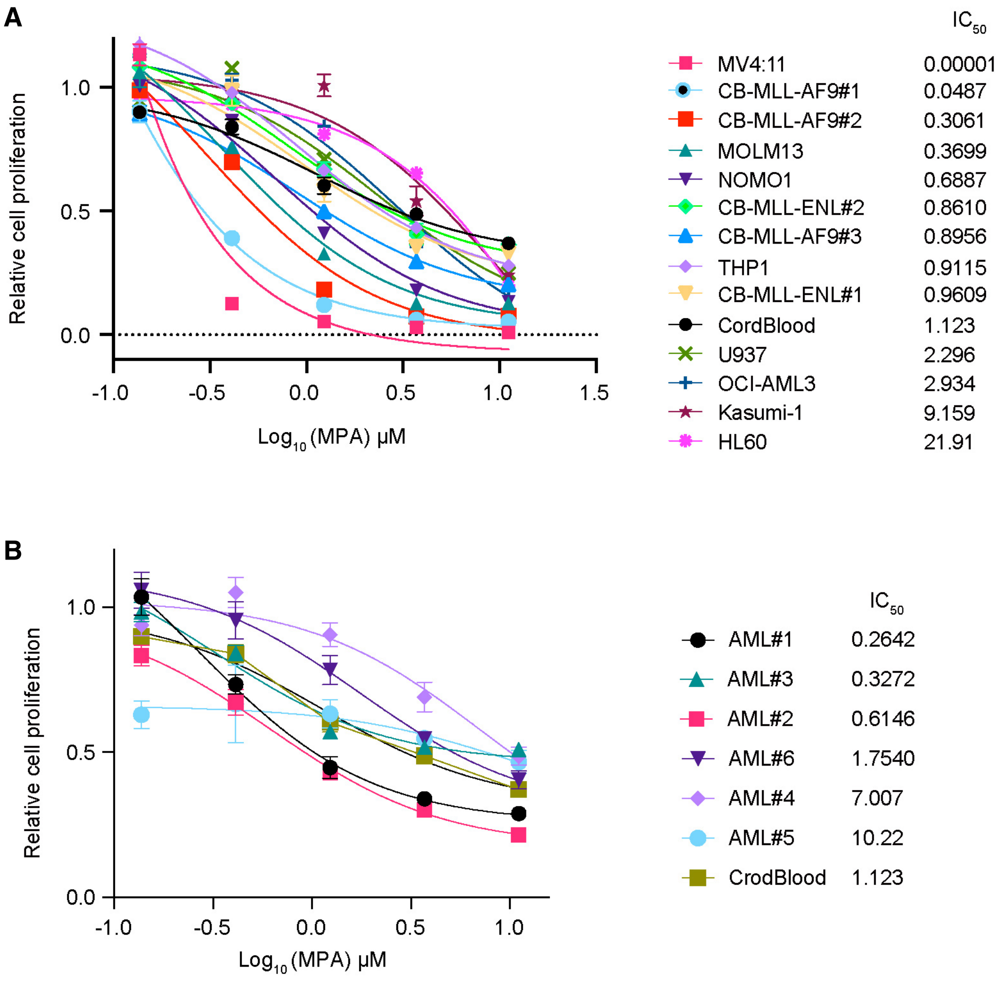

Figure EV1. The effect of IMPDH inhibitors on human myeloid leukemia cells *in vitro*.

A, B Cell viability assays were performed using WST-1. Human CB $CD34^+$ cells and human AML cell lines (with MLL-fusions: MV4;11, MOLM13, NOMO1 and THP1, without MLL-fusions: HL60, Kasumi-1, OCI-AML3 and U937), CB cells expressing MLL-AF9 (CB-MA9#1, 2, 3) or MLL-ENL (CB-MLL-ENL#1, 2) (A) and PDX cell lines with or without MLL-fusions (AML#1, 2, 3 or AML#4, 5, 6) (B) were treated with titrating doses of MPA (1–100 μM) for 72 h in three technical replicates. IC50 values for each experiment are summarized in Fig 1B and C. Data are shown as mean ± SEM.

Source data are available online for this figure.

©2022 The Authors

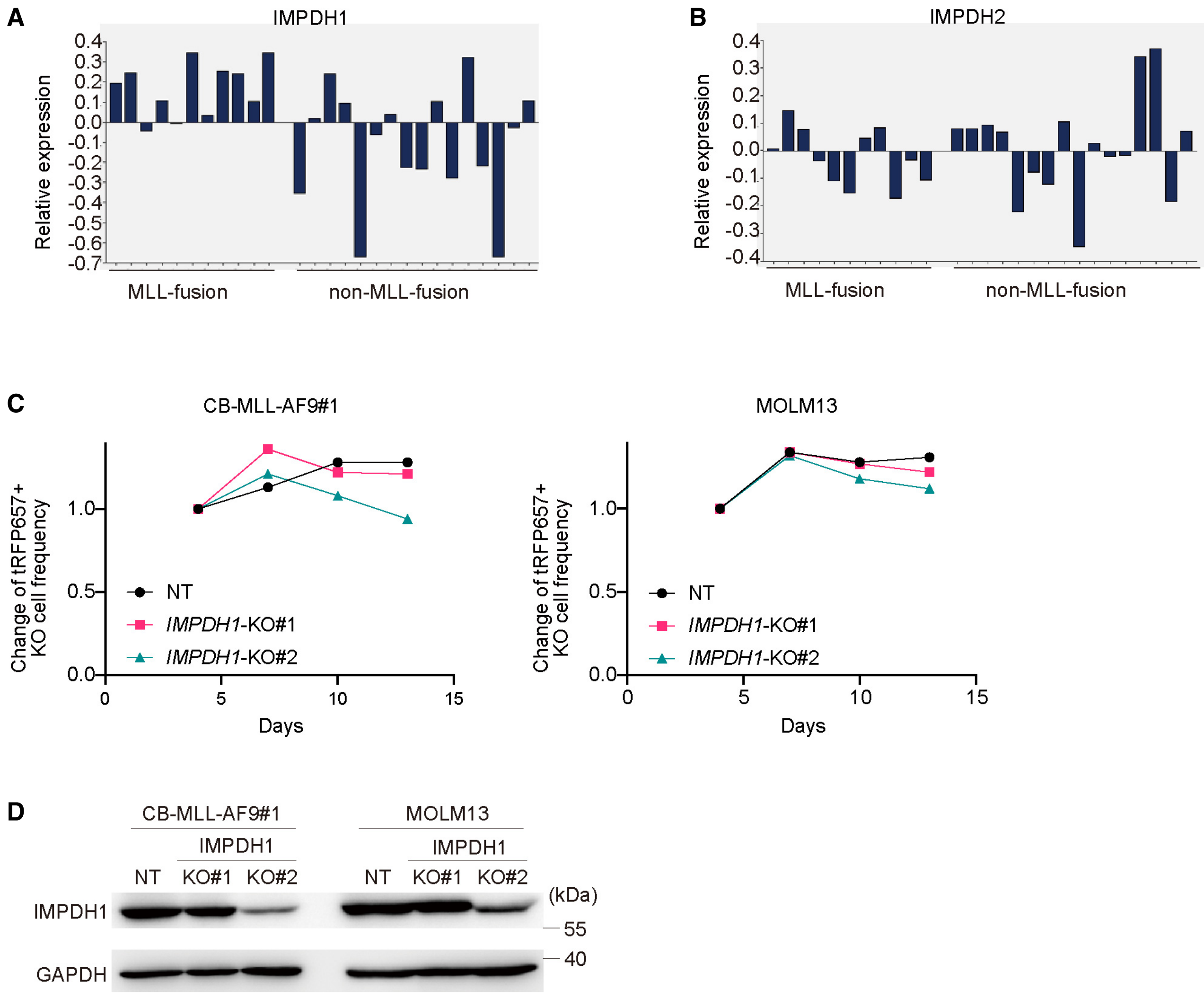

Figure EV2. Dependency of IMPDH1 and IMPDH2 in human AML cells.

A, B Relative mRNA expression of IMPDH1 (A) and IMPDH2 (B) in PDX cells derived from patients with MLL-fusion or non-MLL-fusion AMLs.

C MLL-AF9-expressing CB cells and MOLM13 cells were transduced with Cas9 together with non-targeting (NT) sgRNA or IMPDH1-targeting sgRNAs (IMPDH1-KO1 and KO2) co-expressing tRFP657. Changes in the frequency of tRFP657^{+} cells (sgRNA-transduced cells) in cell cultures are shown. Results are normalized to the frequency of tRFP657^{+} cells at day 4, set to 1.

D The tRFP657^{+} cells were sorted and subjected to western blotting.

Source data are available online for this figure.

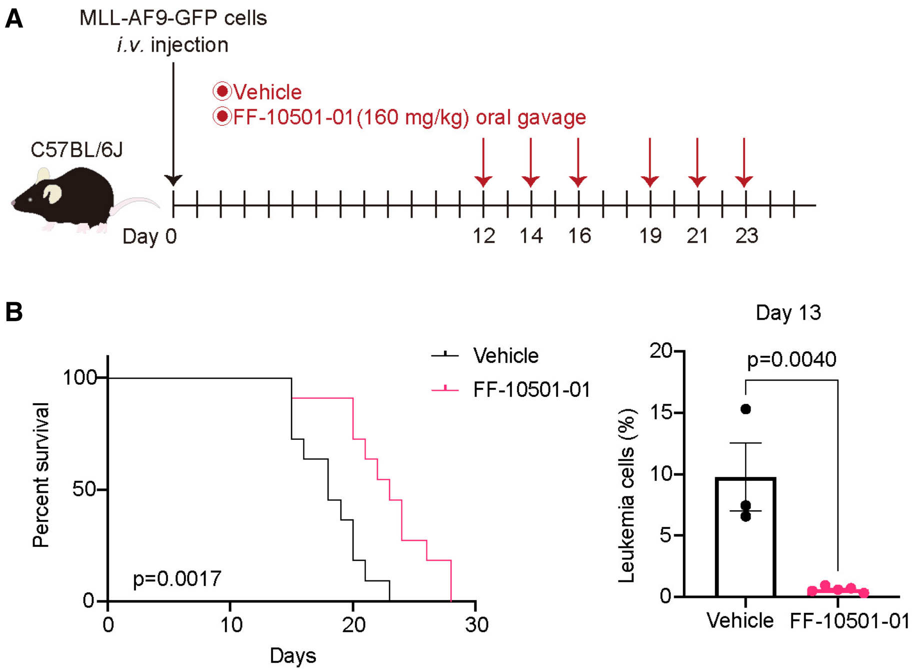

Figure EV3. Therapeutic effect of IMPDH inhibitor is significant on overt leukemia in mice.

A Experimental scheme used in (B). C57BL/6J mice were transplanted with mouse MLL-AF9-GFP cells and were treated with vehicle or FF-10501-01 from day 12 to 23, as indicated. GFP$^+$ MLL-AF9 leukemia cells collected from vehicle- or FF-10501-01-treated mice were analyzed on day 13.

B (left) Kaplan–Meier survival curves of MLL-AF9 leukemia mice treated with vehicle or MMF (n = 11 per group). Statistical significance was evaluated by the log-rank test. (right) Frequencies of GFP$^+$ leukemic cells in peripheral blood at day 13 are also shown (vehicle: n = 3, FF-10501-01: n = 5). A two-tailed unpaired t-test was used for the comparison. Data are shown as mean ± SEM.

Source data are available online for this figure.

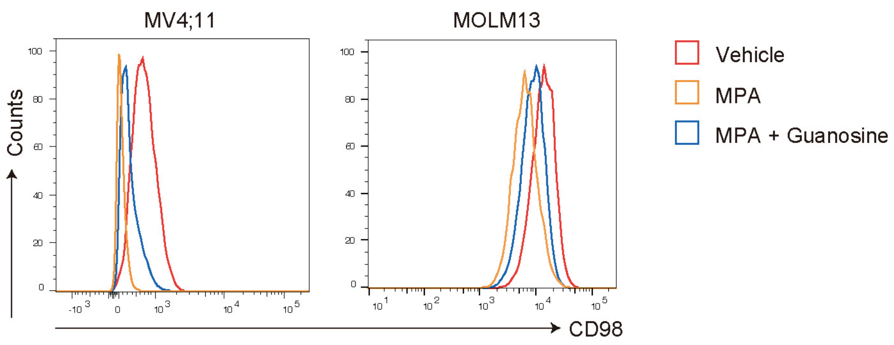

Figure EV4. IMPDH inhibition induces downregulation of CD98 in human AML cell lines *in vitro*.

FCM plots showing the CD98 expression in MV4:11 (left) and MOLM13 cells (right) after treated with/without 1 μM MPA and 100 μM Guanosine for 36 h.

Source data are available online for this figure.

©2022 The Authors
