# Supplementary figures and images for: IMPDH inhibition activates TLR‐VCAM1 pathway and suppresses the development of MLL‐fusion leukemia
Source: EMBO Mol Med. 2022 Dec 1;15(1):e15631. doi: 10.15252/emmm.202115631 (PMC9832838; doi:10.15252/emmm.202115631)

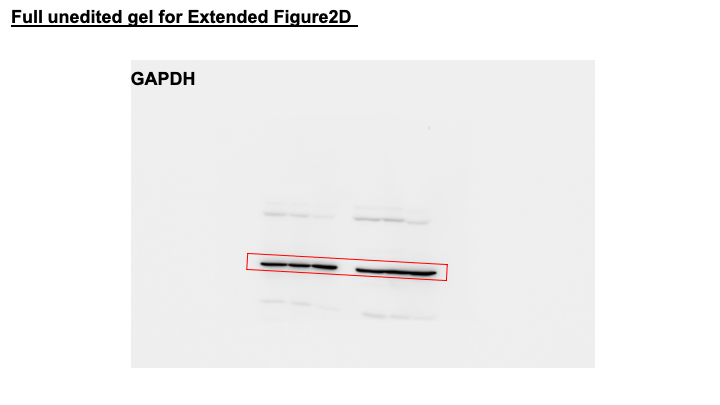

Supplement: Supplementary file 3 — Source Data for Expanded View [file EMMM-15-e15631-s004.zip › Extend Figure2/Extend Figure2D/Western-blotting/GAPDH.tiff]

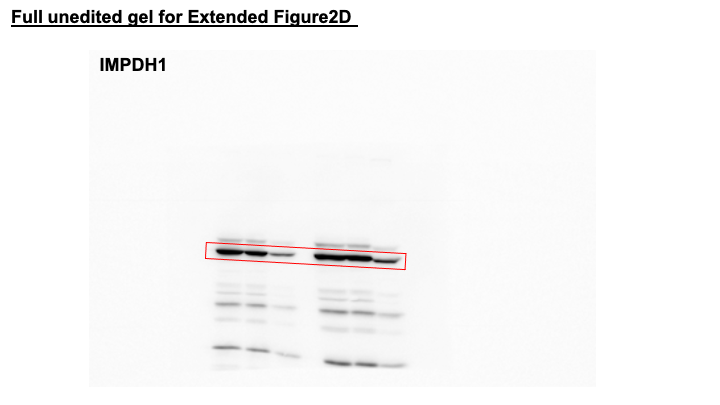

Supplement: Supplementary file 3 — Source Data for Expanded View [file EMMM-15-e15631-s004.zip › Extend Figure2/Extend Figure2D/Western-blotting/IMPDH1.tiff]

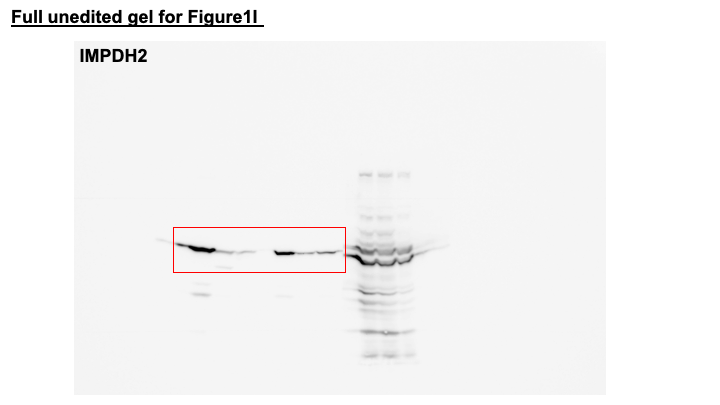

Supplement: Supplementary file 5 — Source Data for Figure 1 [file EMMM-15-e15631-s002.zip › Figure1/Figure 1I/Western-blotting/IMPDH2.tiff]

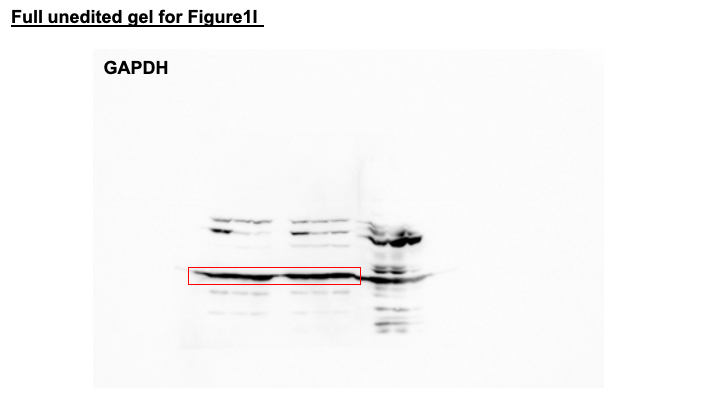

Supplement: Supplementary file 5 — Source Data for Figure 1 [file EMMM-15-e15631-s002.zip › Figure1/Figure 1I/Western-blotting/GAPDH.tiff]

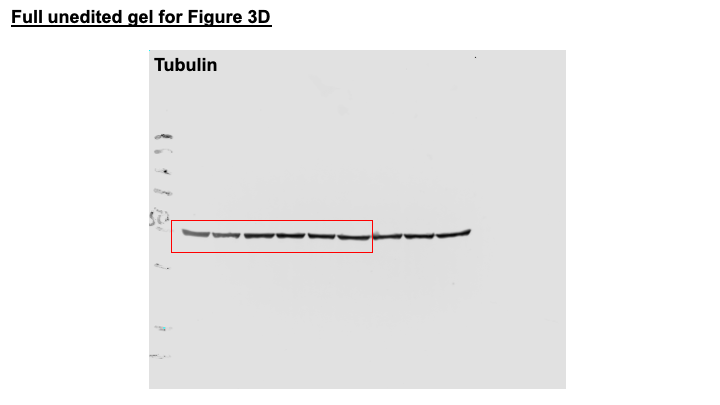

Supplement: Supplementary file 6 — Source Data for Figure 3 [file EMMM-15-e15631-s008.zip › Figure3/Figure3D/Western-blotting/Tubulin.tiff]

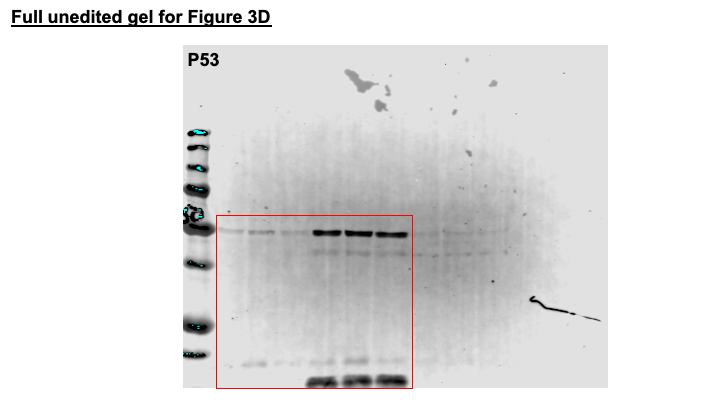

Supplement: Supplementary file 6 — Source Data for Figure 3 [file EMMM-15-e15631-s008.zip › Figure3/Figure3D/Western-blotting/P53.tiff]

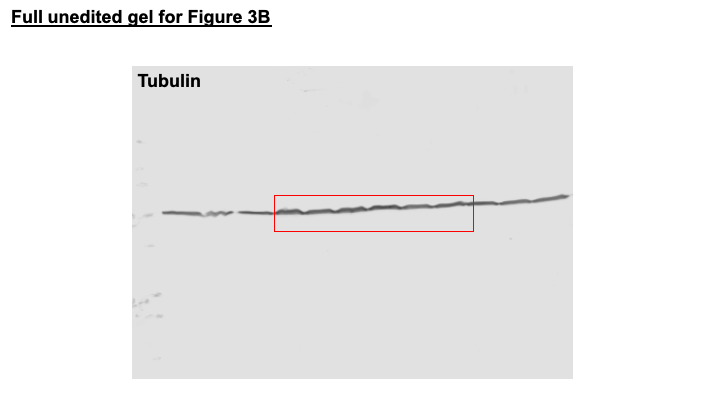

Supplement: Supplementary file 6 — Source Data for Figure 3 [file EMMM-15-e15631-s008.zip › Figure3/Figure3B/Western-blotting/Tubulin.tiff]

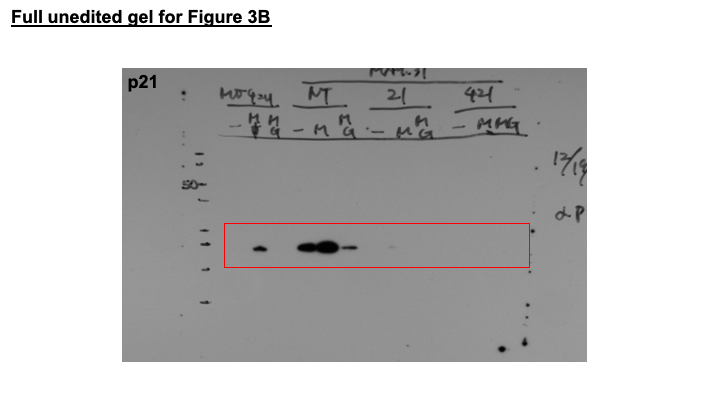

Supplement: Supplementary file 6 — Source Data for Figure 3 [file EMMM-15-e15631-s008.zip › Figure3/Figure3B/Western-blotting/P21.tiff]

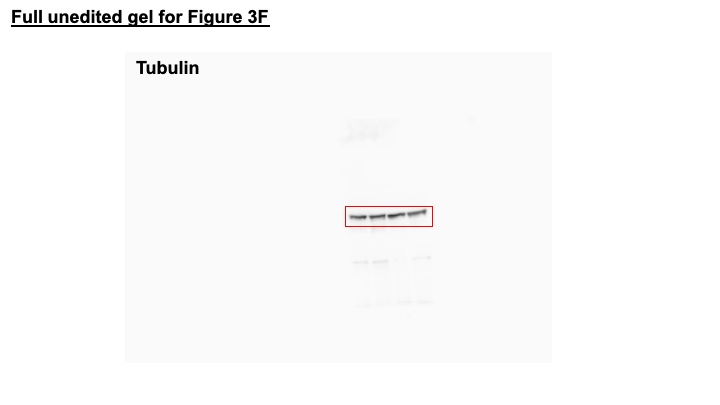

Supplement: Supplementary file 6 — Source Data for Figure 3 [file EMMM-15-e15631-s008.zip › Figure3/Figure3F/Western-blotting/Tubulin.tiff]

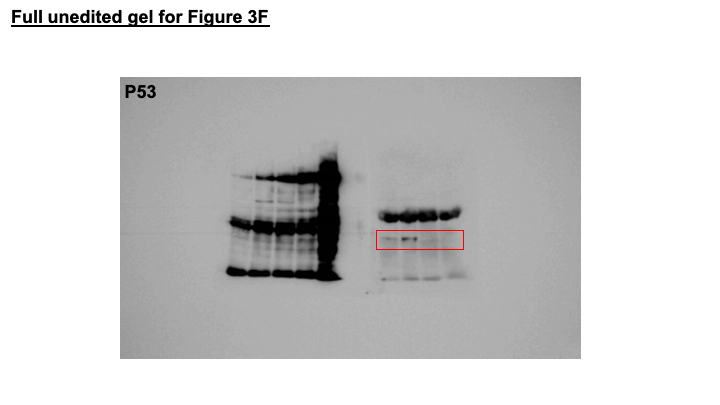

Supplement: Supplementary file 6 — Source Data for Figure 3 [file EMMM-15-e15631-s008.zip › Figure3/Figure3F/Western-blotting/P53.tiff]

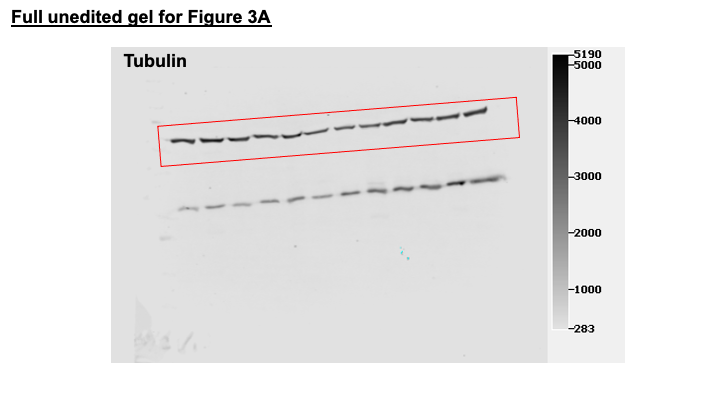

Supplement: Supplementary file 6 — Source Data for Figure 3 [file EMMM-15-e15631-s008.zip › Figure3/Figure3A/Western-blotting/Tubulin.tiff]

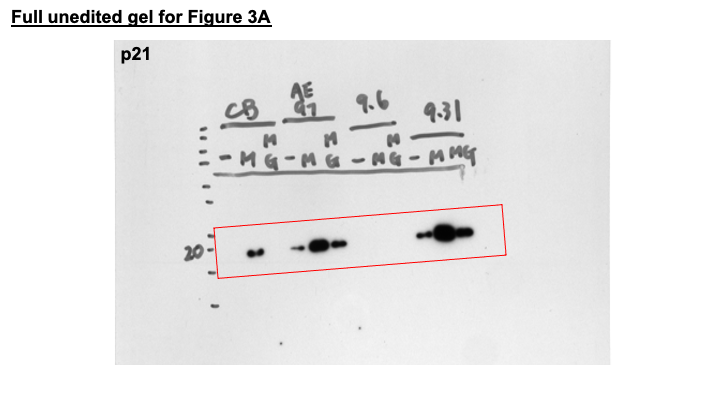

Supplement: Supplementary file 6 — Source Data for Figure 3 [file EMMM-15-e15631-s008.zip › Figure3/Figure3A/Western-blotting/P21.tiff]

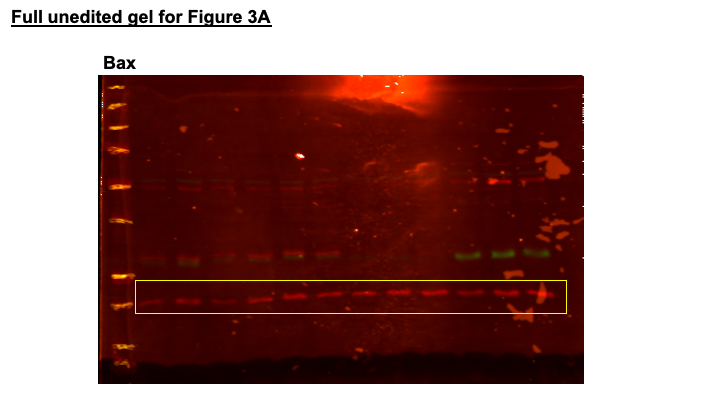

Supplement: Supplementary file 6 — Source Data for Figure 3 [file EMMM-15-e15631-s008.zip › Figure3/Figure3A/Western-blotting/Bax.tiff]

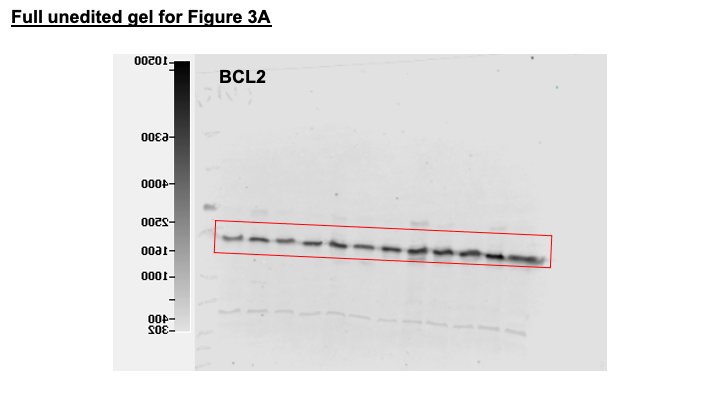

Supplement: Supplementary file 6 — Source Data for Figure 3 [file EMMM-15-e15631-s008.zip › Figure3/Figure3A/Western-blotting/BCL2.tiff]

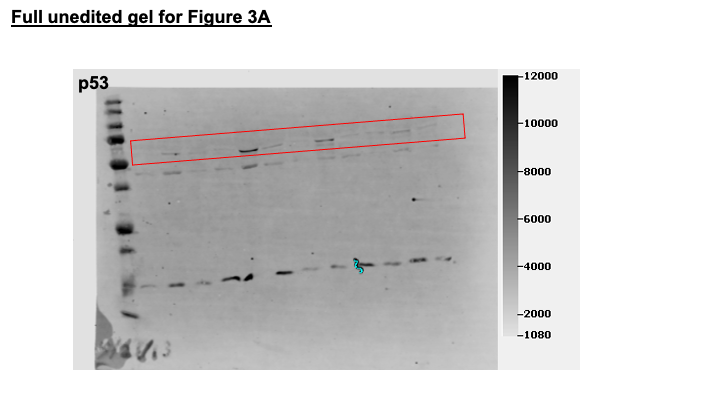

Supplement: Supplementary file 6 — Source Data for Figure 3 [file EMMM-15-e15631-s008.zip › Figure3/Figure3A/Western-blotting/P53.tiff]

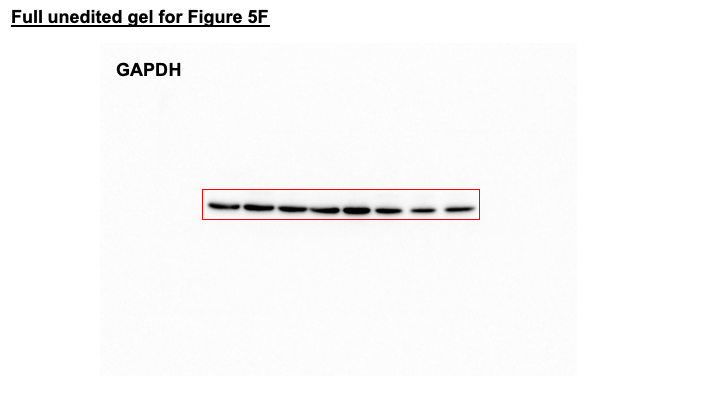

Supplement: Supplementary file 7 — Source Data for Figure 5 [file EMMM-15-e15631-s006.zip › Figure5/Figure5F/Western-blotting/GAPDH.tiff]

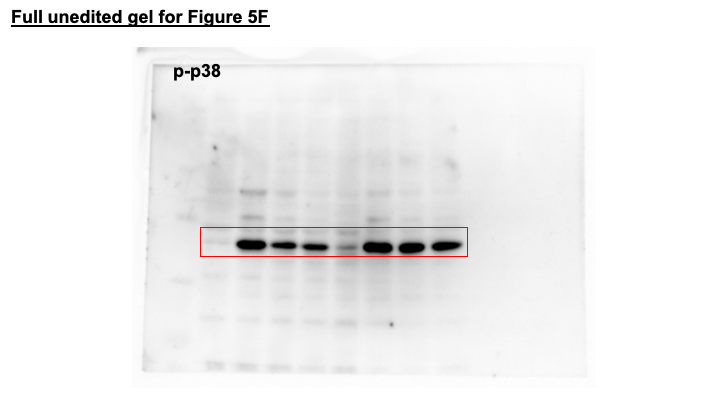

Supplement: Supplementary file 7 — Source Data for Figure 5 [file EMMM-15-e15631-s006.zip › Figure5/Figure5F/Western-blotting/p-p38 .tiff]

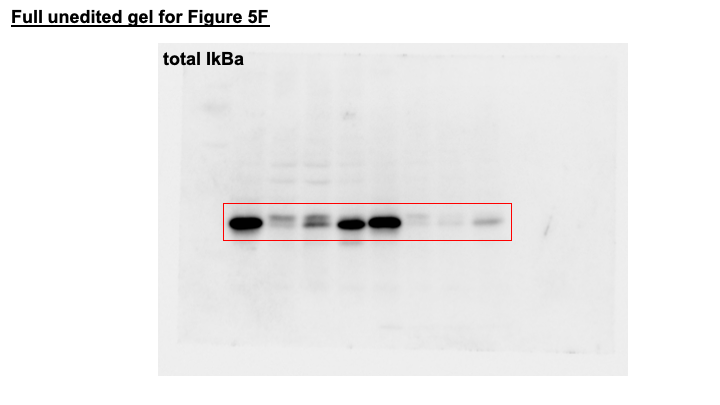

Supplement: Supplementary file 7 — Source Data for Figure 5 [file EMMM-15-e15631-s006.zip › Figure5/Figure5F/Western-blotting/total IkBa .tiff]

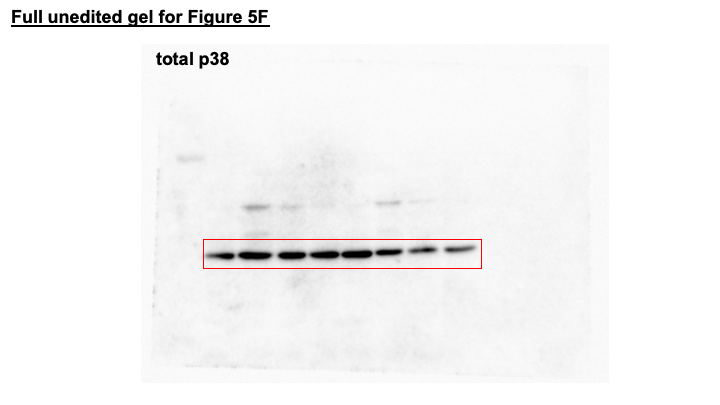

Supplement: Supplementary file 7 — Source Data for Figure 5 [file EMMM-15-e15631-s006.zip › Figure5/Figure5F/Western-blotting/total p38 .tiff]

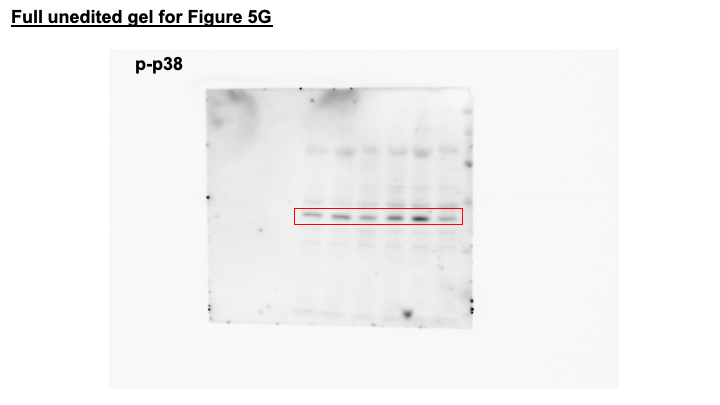

Supplement: Supplementary file 7 — Source Data for Figure 5 [file EMMM-15-e15631-s006.zip › Figure5/Figure5G/Western-blotting/p-p38.tiff]

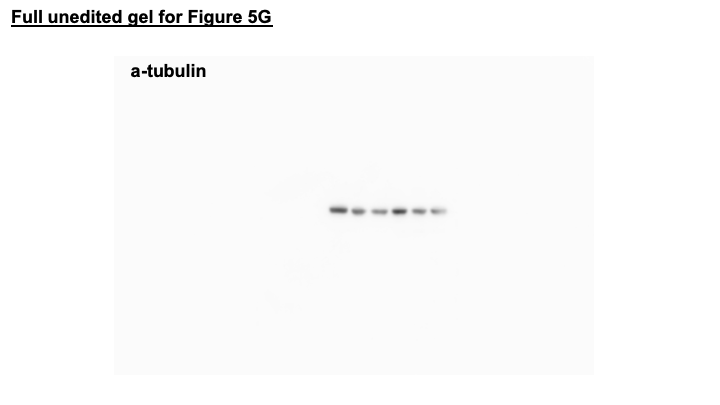

Supplement: Supplementary file 7 — Source Data for Figure 5 [file EMMM-15-e15631-s006.zip › Figure5/Figure5G/Western-blotting/a-tubulin.tiff]

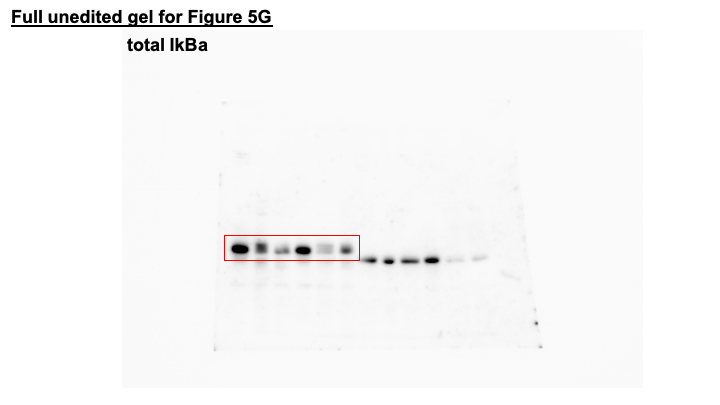

Supplement: Supplementary file 7 — Source Data for Figure 5 [file EMMM-15-e15631-s006.zip › Figure5/Figure5G/Western-blotting/total IkBa .tiff]

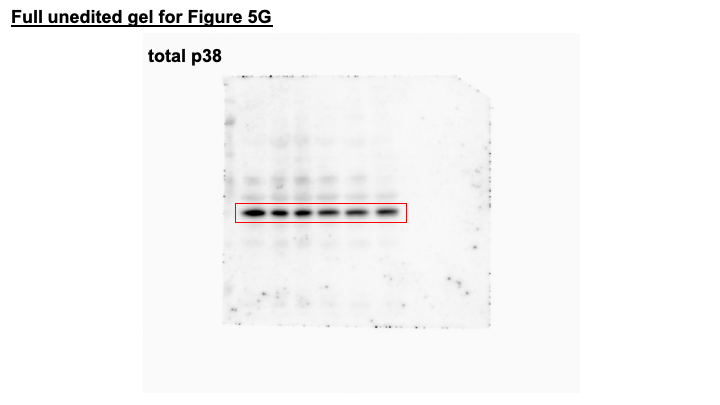

Supplement: Supplementary file 7 — Source Data for Figure 5 [file EMMM-15-e15631-s006.zip › Figure5/Figure5G/Western-blotting/total p38 .tiff]

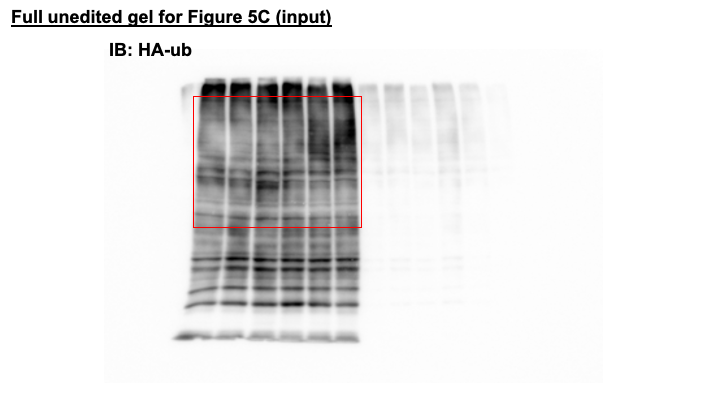

Supplement: Supplementary file 7 — Source Data for Figure 5 [file EMMM-15-e15631-s006.zip › Figure5/Figure5C/Western-blotting/Input_IB- HA-ub .tiff]

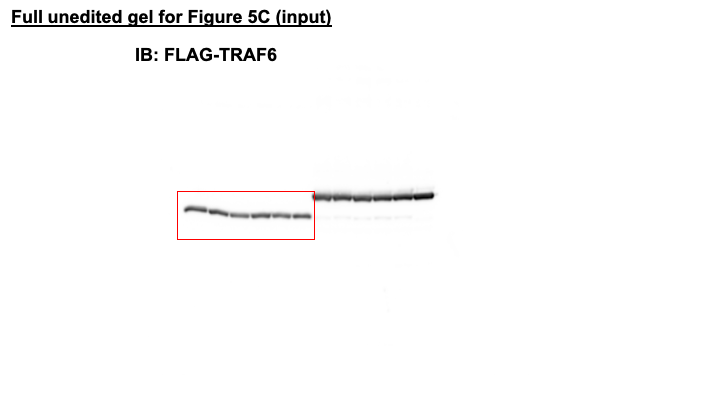

Supplement: Supplementary file 7 — Source Data for Figure 5 [file EMMM-15-e15631-s006.zip › Figure5/Figure5C/Western-blotting/Input_FLAG-TRAF6.tiff]

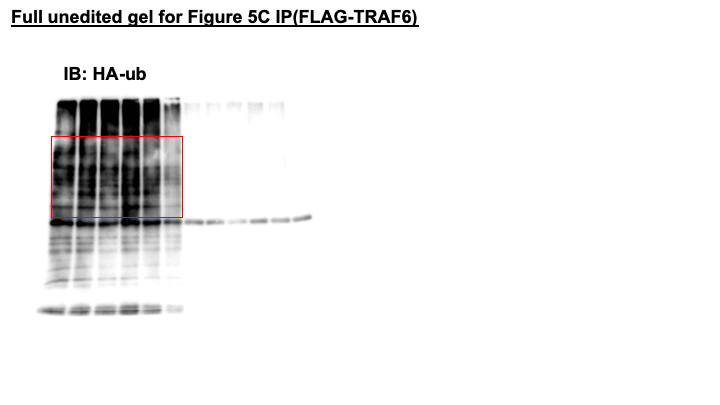

Supplement: Supplementary file 7 — Source Data for Figure 5 [file EMMM-15-e15631-s006.zip › Figure5/Figure5C/Western-blotting/IP_FLAG-TRAF6.tiff]
