# Supplementary material for: IMPDH inhibition activates TLR‐VCAM1 pathway and suppresses the development of MLL‐fusion leukemia
Source: EMBO Mol Med. 2022 Dec 1;15(1):e15631. doi: 10.15252/emmm.202115631 (PMC9832838; doi:10.15252/emmm.202115631)
Supplement: Supplementary file 4 — PDF+ [file EMMM-15-e15631-s003.pdf]

Article

SOURCE DATA | TRANSPARENT PROCESS | OPEN ACCESS

EMBO Molecular Medicine

IMPDH inhibition activates TLR-VCAM1 pathway and suppresses the development of MLL-fusion leukemia

Xiaoxiao Liu[1], Naru Sato[2], Tomohiro Yabushita[2], Jingmei Li[1], Yuhan Jia[1], Moe Tamura[1], Shuhei Asada[2,3], Takeshi Fujino[2], Tsuyoshi Fukushima[2], Taishi Yonezawa[1], Yosuke Tanaka[2], Tomofusa Fukuyama[2], Akiho Tsuchiya[2], Shiori Shikata[2], Hiroyuki Iwamura[4], Chieko Kinouchi[5], Kensuke Komatsu[5], Satoshi Yamasaki[6], Tatsuhiro Shibata[6], Atsuo T Sasaki[7], Janet Schibler[8], Mark Wunderlich[8], Eric O'Brien[9], Benjamin Mizukawa[8], James C Mulloy[8], Yuki Sugiura[10], Hitoshi Takizawa[11], Takuma Shibata[12], Kensuke Miyake[12], Toshio Kitamura[2] & Susumu Goyama[1,*]

Abstract

Inosine monophosphate dehydrogenase (IMPDH) is a rate-limiting enzyme in *de novo* guanine nucleotide synthesis pathway. Although IMPDH inhibitors are widely used as effective immunosuppressants, their antitumor effects have not been proven in the clinical setting. Here, we found that acute myeloid leukemias (AMLs) with MLL-fusions are susceptible to IMPDH inhibitors *in vitro*. We also showed that alternate-day administration of IMPDH inhibitors suppressed the development of MLL-AF9-driven AML *in vivo* without having a devastating effect on immune function. Mechanistically, IMPDH inhibition induced overactivation of Toll-like receptor (TLR)-TRAF6-NF-κB signaling and upregulation of an adhesion molecule VCAM1, which contribute to the antileukemia effect of IMPDH inhibitors. Consequently, combined treatment with IMPDH inhibitors and the TLR1/2 agonist effectively inhibited the development of MLL-fusion AML. These findings provide a rational basis for clinical testing of IMPDH inhibitors against MLL-fusion AMLs and potentially other aggressive tumors with active TLR signaling.

Keywords IMPDH inhibitor; MLL-fusion leukemia; TLR signaling; Vcam1
Subject Categories Cancer; Haematology
DOI 10.15252/emmm.202115631 | Received 22 December 2021 | Revised 8 November 2022 | Accepted 8 November 2022 | Published online 1 December 2022

EMBO Mol Med (2023) 15: e15631
See also: **M Sezaki & G Huang** (January 2023)

Introduction

MLL-fusion leukemias are aggressive forms of acute leukemias carrying chimeric fusion of the *MLL* (also called *KMT2A*) gene. The *MLL* gene encodes a histone H3 lysine 4 (H3K4) methyltransferase and is the frequent target of chromosomal translocations, resulting in the fusion of the 5′ portion of *MLL* to a number of different partner genes (Yokoyama, 2015; Slany, 2016). MLL-fusions, such as MLL-AF9, MLL-AF4, and MLL-ENL, are recurrently found in a subset of acute myeloid leukemia (AML) and B-cell precursor acute lymphoblastic leukemia (B-ALL). The MLL-fusion proteins lose the H3K4 methyltransferase activity but instead acquire aberrant functions for epigenetic regulation. Despite recent advances in understanding molecular pathogenesis and therapeutic approaches, many patients with MLL-fusion leukemia still have poor outcomes.

Studies have identified crucial factors that are involved in MLL-fusion driven leukemogenesis, including interacting proteins (Yokoyama *et al*, 2005; Yokoyama & Cleary, 2008), epigenetic regulators (Okada *et al*, 2005; Zuber *et al*, 2011b; Harris *et al*, 2012; Neff *et al*, 2012; Asada *et al*, 2018), transcription factors (Zuber

1 Division of Molecular Oncology, Department of Computational Biology and Medical Sciences, Graduate School of Frontier Sciences, The University of Tokyo, Tokyo, Japan
2 Division of Cellular Therapy, The Institute of Medical Science, The University of Tokyo, Tokyo, Japan
3 The Institute of Laboratory Animals, Tokyo Women's Medical University, Tokyo, Japan
4 FUJIFILM Corporation: Pharmaceutical Products Division, Tokyo, Japan
5 FUJIFILM Corporation: Bio Science & Engineering Laboratories, Kanagawa, Japan
6 Laboratory of Molecular Medicine, Human Genome Center, The Institute of Medical Science, The University of Tokyo, Tokyo, Japan
7 Division of Hematology and Oncology, Department of Internal Medicine, University of Cincinnati, Cincinnati, OH, USA
8 Division of Experimental Hematology and Cancer Biology, Cincinnati Children's Hospital Medical Center, Cincinnati, OH, USA
9 Division of Oncology, Department of Pediatrics, University of Cincinnati, Cincinnati, OH, USA
10 Department of Biochemistry, Keio University School of Medicine, Tokyo, Japan
11 Laboratory of Stem Cell Stress, International Research Center for Medical Sciences, Kumamoto University, Kumamoto, Japan
12 Division of Innate Immunity, Department of Microbiology and Immunology, The Institute of Medical Science, The University of Tokyo, Tokyo, Japan
*Corresponding author. Tel: +81-3-5449-5518; Fax: +81-3-5449-5453; E-mail: goyama@edu.k.u-tokyo.ac.jp

©2022 The Authors. Published under the terms of the CC BY 4.0 license.

et al, 2011a; Goyama *et al*, 2013; Ye *et al*, 2015), and signaling molecules (Wang *et al*, 2008, 2010; Kuo *et al*, 2013). In AML, *MLL* rearrangements are most strongly associated with French-American-British subtypes M4/M5 that have monocytic/macrophage-like characteristics (Tien *et al*, 2000). Because Toll-like receptors (TLRs) constitute a key signaling system in these innate immune cells (Takeda & Akira, 2005), the TLR pathways could also be therapeutic targets in MLL-fusion AMLs. Toll-like receptors mainly transduce signals through an adaptor protein MyD88. MyD88 forms the Myddosome with IRAK kinases, which induces association and activation of the E3 ubiquitin ligase TRAF6. TRAF6 promotes K63-linked polyubiquitination of the protein kinase TAK1, and TAK1 then activates NF-κB and MAPK pathways. The activated NF-κB induces expression of pro-inflammatory cytokines and adhesion molecules, such as TNF-α, IL-6, and Vcam1. The active IRAK-NF-κB pathway has been shown to be necessary to maintain aberrant epigenetic programs induced by MLL-fusion proteins (Goyama & Mulloy, 2013; Kuo *et al*, 2013; Liang *et al*, 2017). Importantly, excessive activation of the TLR-NF-κB pathway could also prevent tumor development. Indeed, studies have shown that agonistic targeting TLR1/2 or TLR8 induced apoptosis and differentiation of MLL-fusion AMLs (Ignatz-Hoover *et al*, 2015; Eriksson *et al*, 2017).

Uncontrolled cell proliferation is a hallmark of tumors, including MLL-fusion leukemia, which requires adequate nucleotide biosynthesis. Guanine nucleotides play essential roles in diverse biological processes and are synthesized *de novo* or recycled via salvage pathways. Inosine monophosphate dehydrogenase (IMPDH) is a rate-limiting enzyme in *de novo* guanine nucleotide synthesis pathway, which is responsible for the conversion of IMP to XMP (Hedstrom, 2009; Naffouje *et al*, 2019; Fig 1A). In mammals, there are two IMPDH isotypes: IMPDH1 and IMPDH2. IMPDH2 is a predominant isotype in most tissues, especially in tumors and highly replicative cells (Collart *et al*, 1992). Several IMPDH inhibitors, such as Mycophenolic acid (MPA) and mizoribine, are widely used as immunosuppressants for the prevention of organ transplant rejection (Naffouje *et al*, 2019). In addition, recent studies have shown the antitumor activity of IMPDH inhibition against a variety of tumors, such as glioblastoma (Kofuji *et al*, 2019), ASCL1-low-small-cell lung cancer (Huang *et al*, 2018), relapsed ALL with NT5C2 mutations (Tzoneva *et al*, 2018) and AML cell lines (Murase *et al*, 2016; Yang *et al*, 2017). In clinical trials, tiazofurin and FF-10501-01 (a prodrug of mizoribine) showed clinical activity in patients with myeloid neoplasms (Tricot *et al*, 1989; Garcia-Manero *et al*, 2020). However, the clinical trial using FF-10501-01 was discontinued due to increased mucositis events (Garcia-Manero *et al*, 2020), indicating that further optimization of dose schedule is required to minimize side effects of IMPDH inhibitors. Another concern for the use of IMPDH inhibitors as anticancer drugs is their immunosuppressive activity. Given the importance of tumor immunosurveillance, the therapeutic effects of IMPDH inhibitors could be attenuated *in vivo*.

In this study, we showed the potent antileukemia effect of two IMPDH inhibitors, MPA and FF-10501-01, on MLL-fusion AMLs. Importantly, we found that alternate-day administration of IMPDH inhibitors to mice suppressed the development of MLL-AF9-driven AML *in vivo* without substantially reducing the number of immune cells. Our study also revealed the unexpected effect of IMPDH inhibitors to induce overactivation of the TLR signaling and upregulation of VCAM1 in AML cells, which is likely to contribute to their antileukemia effects.

Results

Inhibition of IMPDH suppresses the growth of MLL-fusion leukemia cells *in vitro*

We first assessed the effect of MPA on several human AML cell lines (with MLL-fusions: MV4;11, MOLM13, NOMO1 and THP1, without MLL-fusions: HL60, Kasumi-1, OCI-AML3 and U937), human cord blood (CB) $CD34^+$ cells, and those expressing MLL-AF9 (CB-MA9#1, 2, 3) or MLL-ENL (CB-MLL-ENL#1, 2), and patient-derived xenograft (PDX) cell lines derived from AML patients with or without MLL-fusions (AML#1, 2, 3 or AML#4, 5, 6, Appendix Table S1). Most of the AML cells with MLL-fusions are more sensitive to MPA than normal CB cells and non-MLL-fusion AMLs (Figs 1B and C, and EV1; Appendix Fig S1), indicating a high dependency of MLL-fusion AMLs on the guanine nucleotide synthesis pathway. RNA levels of IMPDH1 and IMPDH2 differed substantially between individuals and were not correlated with the MLL-fusions (Fig EV2A

Figure 1. IMPDH inhibitors suppress the growth of MLL-fusion leukemia *in vitro*.

A Schematic of *de novo* purine nucleotide synthesis.

B, C IC_{50} values for MPA-response curves using cell viability assay were shown. Human AML cell lines (with MLL-fusions: MV4;11, MOLM13, NOMO1 and THP1, without MLL-fusions: HL60, Kasumi-1, OCI-AML3 and U937), human cord blood (CB) $CD34^+$ cells, and those expressing MLL-AF9 (CB-MLL-AF9#1, 2, 3) or MLL-ENL (CB#MLL-ENL-1, 2), and patient cells with or without MLL-fusions (AML#1, 2, 3 or AML#4, 5, 6) were treated with titrating doses of MPA (1–100 μM) for 72 h in three technical replicates.

D Response curves of FF-10501-01 (1–100 μM) for 72 h in three technical replicates.

E FCM plots of the cell cycle and apoptosis analyses in MLL-AF9 expressing CB cells and MV4;11 cells. Cells were incubated with/without 1 μM MPA and 100 mM Guanosine for 36 h. Numbers indicate the frequency of S/G2/M-phase cells (left) and Annexin V^+ cells (right).

F, G Immunophenotypes and Wright-Giemsa staining of MLL-AF9 expressing CB cells after 4 days of culture with/without 3.3 μM MPA and 100 μM Guanosine (Scale bar: 20 mm).

H MLL-ENL-expressing CB cells and MOLM13 cells were transduced with Cas9 together with non-targeting (NT) sgRNA or IMPDH2-targeting sgRNAs (IMPDH2-KO1 and KO2) co-expressing tRFP657. Changes in the frequency of $tRFP657^+$ cells (sgRNA-transduced cells) in cell cultures are shown. Results are normalized to the frequency of $tRFP657^+$ cells at day 4, set to 1.

I The $tRFP657^+$ cells in (H) were sorted and subjected to western blotting. The depletion of IMPDH2 protein in MLL-ENL cells and MOLM13 cells was confirmed by western blotting.

Data information: All data are shown as mean ± SEM.
Source data are available online for this figure.

©2022 The Authors

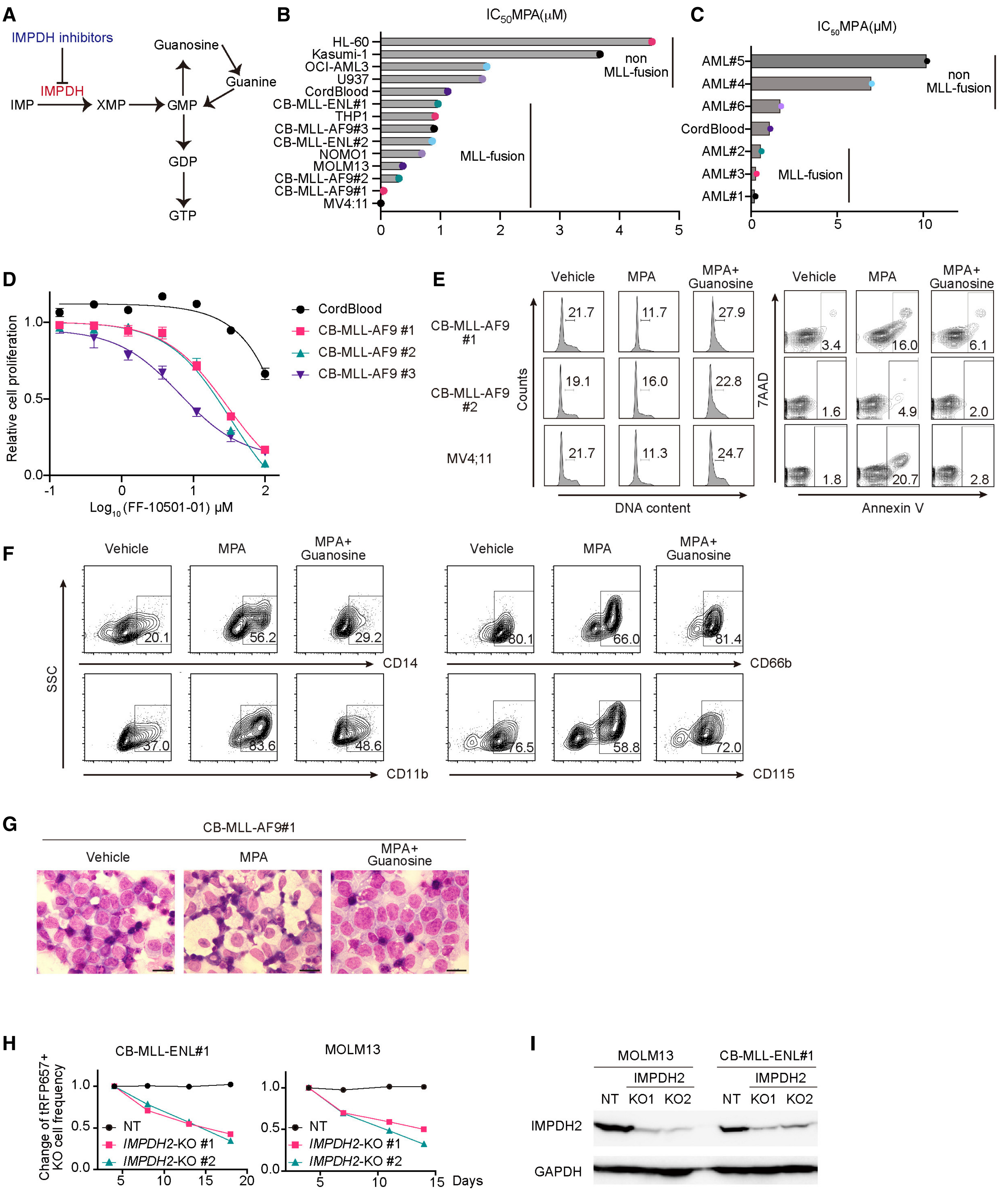
A
IMPDH inhibitors
IMPDH
IMP
XMP
GMP
Guanosine
Guanine
GDP
GTP
B
IC50MPA(μM)
HL-60
Kasumi-1
OCI-AML3
U937
CordBlood
CB-MLL-ENL#1
THP1
CB-MLL-AF9#3
CB-MLL-ENL#2
NOMO1
MOLM13
CB-MLL-AF9#2
CB-MLL-AF9#1
MV4:11
non MLL-fusion
MLL-fusion
C
IC50MPA(μM)
AML#5
AML#4
AML#6
CordBlood
AML#2
AML#3
AML#1
non MLL-fusion
MLL-fusion
D
Relative cell proliferation
Log10(FF-10501-01) μM
CordBlood
CB-MLL-AF9 #1
CB-MLL-AF9 #2
CB-MLL-AF9 #3
E
Vehicle
MPA
MPA+ Guanosine
CB-MLL-AF9 #1
CB-MLL-AF9 #2
MV4;11
Counts
DNA content
7AAD
Annexin V
21.7
11.7
27.9
19.1
16.0
22.8
21.7
11.3
24.7
3.4
16.0
6.1
1.6
4.9
2.0
1.8
20.7
2.8
F
Vehicle
MPA
MPA+ Guanosine
SSC
CD14
CD11b
CD66b
CD115
20.1
56.2
29.2
37.0
83.6
48.6
80.1
66.0
81.4
76.5
58.8
72.0
G
CB-MLL-AF9#1
Vehicle
MPA
MPA+ Guanosine
H
CB-MLL-ENL#1
MOLM13
Change of tRFP657+ KO cell frequency
NT
IMPDH2-KO #1
IMPDH2-KO #2
Days
I
MOLM13
CB-MLL-ENL#1
IMPDH2
NT
KO1
KO2
GAPDH

Figure 1.

and B), indicating that expression of IMPDH1/2 did not predict sensitivity of each PDX-AML to MPA. Another IMPDH inhibitor FF-10501-01 also showed the growth-inhibitory effect on MLL-AF9-expressing CB cells, while it showed only modest effect on normal CB cells (Fig 1D). Mechanistically, MPA treatment caused a cell cycle arrest at G0/G1-phase and triggered apoptosis in MLL-AF9-expressing CB cells, MV4;11 cells and MOLM13 cells (Fig 1E; Appendix Fig S2A and B). Furthermore, MPA-induced differentiation of MLL-AF9-expressing CB cells, as evidenced by the changes in surface marker expression or morphological changes (Fig 1F and G). These MPA-induced changes in MLL-fusion leukemia cells were reversed by the supplementation of guanosine (Fig 1E–G; Appendix Fig S1), confirming that guanine nucleotide depletion underlies the effects of MPA. We also found that MPA synergized with a BCL2 inhibitor venetoclax, but not with a hypomethylating agent decitabine, to inhibit the growth of MLL-AF9-expressing CB cells (Appendix Fig S3A and B). We then assessed the role of IMPDH1 and IMPDH2 in MLL-fusion leukemias using the CRISPR/Cas9 system. Genetic depletion of IMPDH2 inhibited the growth of MOLM13 cells and MLL-ENL-expressing CB cells, while IMPDH1 depletion showed little effects on their growth. (Figs 1H and I, and EV2C and D). These data indicate that IMPDH2 is a key molecule in MLL-fusion leukemia. Thus, IMPDH inhibition suppresses cell cycle progression, induces apoptosis, and enhances differentiation in MLL-fusion AMLs, thereby inhibits their growth.

Because MLL-fusions are also known to drive the development of B-ALL, we then assessed the effect of MPA on CB cells expressing MLL-Af4 that recapitulate t (4;11) pro-B ALL (Lin *et al*, 2016) using a coculture assay with murine stromal cell line MS-5 (Appendix Fig S4A). Addition of MPA induced a dramatic decrease in the formation of leukemic cobblestone-forming cells with a concomitant increase of $CD10^+$ expression in MLL-Af4 cells in a concentration-dependent manner (Appendix Fig S4B and C). Furthermore, MPA inhibited the cobblestone formation of PDX cells derived from two B-ALL patients (ALL#1 and ALL#2) at relatively low concentration (1 μM). The MPA-induced inhibition of cobblestones formation of ALL#1 cells was partially reversed by guanosine supplementation (Appendix Table S1 and Appendix Fig S4D and E). Thus, IMPDH inhibitors show robust growth-inhibitory effects on leukemias with MLL-fusions *in vitro*.

IMPDH inhibitors suppress the development of MLL-AF9-driven AML without eradicating immune cells in mice

We next assessed the *in vivo* effect of IMPDH inhibitors using a mouse AML model driven by MLL-AF9. Mouse bone marrow (BM) progenitors were transduced with MLL-AF9 (co-expressing GFP) and were transplanted into C57BL/6J recipient mice. MLL-AF9-expressing AML cells were collected from moribund mice and used for serial transplantation. The mice that received MLL-AF9 cells were then treated with vehicle or IMPDH inhibitors every other day from Day 1 (Fig 2A). Treatment with both mycophenolate mofetil (MMF; a prodrug of MPA, 120 mg/kg) and FF-10501-01 (160 mg/kg) inhibited the engraftment of MLL-AF9 cells and significantly prolonged survival of these mice (Fig 2B and C). Delayed initiation of FF-10501-01 (from Day 12 to Day 23) still prolonged the survival of MLL-AF9 leukemia mice, with reduction of GFP^+ leukemia cells in peripheral blood (Fig EV3A and B). To assess the mechanisms of drug action *in vivo*, we then treated the mice with IMPDH inhibitors for two consecutive days and analyzed the bone marrow cells on the next day (Fig 2D). We found that FF-10501-01 induced myeloid differentiation of MLL-AF9 cells *in vivo*, as evidenced by the characteristic morphological changes (Fig 2E), the increase in $Mac\text{-}1^+Gr\text{-}1^+$ cells, and the decrease in $c\text{-}Kit^+Gr\text{-}1^-$ cells (Fig 2F). By contrast, FF-10501-01 did not induce cell cycle arrest and apoptosis in MLL-AF9 cells in this experimental condition (Fig 2G).

To assess the effect of IMPDH inhibition on human AML cells *in vivo*, we transplanted the MPA-sensitive PDX cells with MLL-fusions (Appendix Table S1) to the immunodeficient NOD/RAG1/$2^{-/-}IL2R\gamma^{-/-}$ (NRGS) mice expressing human SCF, IL-3, and GM-CSF (Barve *et al*, 2018), and treated them with IMPDH inhibitors (MMF or FF-10501-01) for 3 weeks (Fig 2H). Although both inhibitors showed a weak tendency to reduce the engraftment of human AML cells in bone marrow, neither of them improved the survival

Figure 2. IMPDH inhibitors suppress the growth of MLL-fusion leukemia *in vivo*.

A Experimental scheme used in (B and C). C57BL/6J mice were transplanted with MLL-AF9-GFP cells and were treated with vehicle or MMF or FF-10501-01.
B Kaplan–Meier survival curves of MLL-AF9 leukemia mice treated with vehicle or MMF (n = 12 per group). Frequencies of GFP^+ leukemic cells in peripheral blood at day10 are also shown (mean ± SEM) on the right side (vehicle: n = 3, MMF: n = 4). Unpaired Student's t-test (two-tailed) were used for pairwise comparisons of significance.
C Kaplan–Meier survival curves with vehicle (n = 5), FF-10501-01 (n = 10). Frequencies of GFP^+ leukemic cells in peripheral blood at day10 are also shown (mean ± SEM) on the right side (vehicle: n = 5, FF-10501-01: n = 5). Unpaired Student's t-test (two-tailed) were used for pairwise comparisons of significance.
D Experimental scheme used in (E-G). C57BL/6J mice were transplanted with MLL-AF9-GFP cells and were treated with vehicle or FF-10501-01 on day 11 and 12. GFP^+ MLL-AF9 leukemia cells collected from mice were analyzed on day 13.
E Wright-Giemsa staining of MLL-AF9 cells (Scale bar: 20 μm).
F Representative FCM plots of frequencies of $c\text{-}Kit^+$ $Gr\text{-}1^-$ and $Mac\text{-}1^+$ $Gr\text{-}1^+$ in leukemic cells and their quantification of independent mice (n = 10 per group). Unpaired Student's t-test (two-tailed) were used for pairwise comparisons of significance.
G Cell-cycle status (left) and apoptosis (right) were assessed. Frequencies of S/G2/M phase cells and Annexin V^+ cells in GFP^+ MLL-AF9 leukemia cells (n = 10 per group). Two-tailed unpaired t-tests were used for the comparison.
H Experimental scheme used in (I and J). MPA-sensitive patient AML#1 (MLL-AF10) cells were transplanted to NRGS mice and treated with vehicle, MMF or FF-10501-01 5 days per week for 3 weeks.
I Kaplan–Meier survival curves of PDX-transplanted leukemia mice treated with vehicle (n = 6), MMF (n = 5), or FF-10501-01 (n = 5).
J Frequency of human CD45+ cells in bone marrow at day 22 in mice described in (I). Ordinary one-way ANOVA was used for the comparison.
K Experimental scheme (left). MLL-AF9-GFP cells were transplanted into immunocompetent C57BL/6J mice and immunodeficient NSG mice. Dose schedule with vehicle or FF-10501-01 are the same as described in (A). Kaplan–Meier survival curves were also shown on the right (NSG-vehicle: n = 8, NSG-FF-10501-01: n = 8, C57BL/6J-vehicle: n = 6, C57BL/6J-FF-10501-01: n = 6).

Data information: All data are shown as mean ± SEM. Log-rank test was used to compare the survival curves.

 ©2022 The Authors

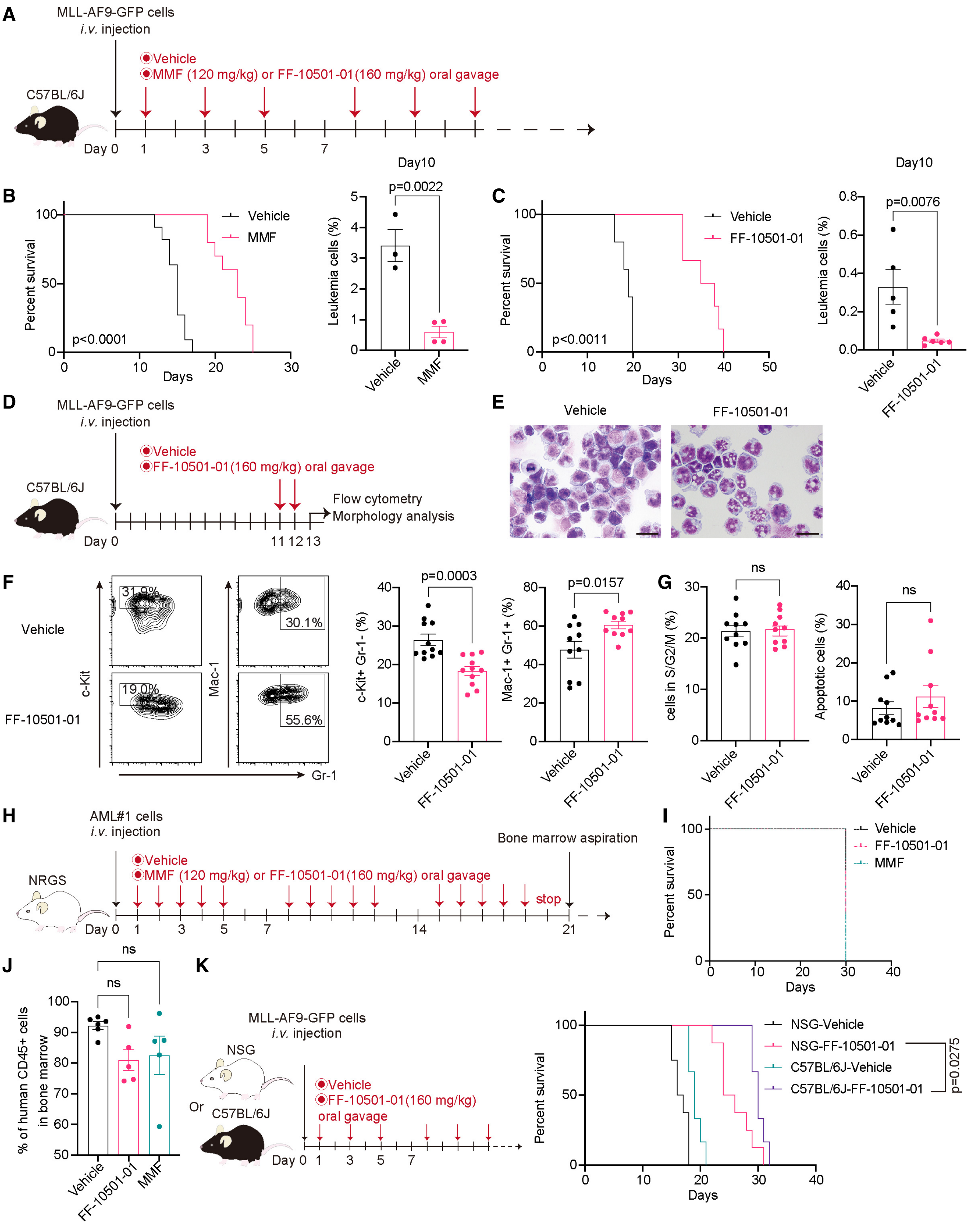
A
MLL-AF9-GFP cells
i.v. injection
Vehicle
MMF (120 mg/kg) or FF-10501-01(160 mg/kg) oral gavage
C57BL/6J
Day 0 1 3 5 7
B
Vehicle
MMF
Percent survival
p<0.0001
Days
Day10
p=0.0022
Leukemia cells (%)
C
Vehicle
FF-10501-01
p<0.0011
p=0.0076
D
MLL-AF9-GFP cells
i.v. injection
Vehicle
FF-10501-01(160 mg/kg) oral gavage
Flow cytometry
Morphology analysis
11 12 13
E
Vehicle
FF-10501-01
F
c-Kit
Mac-1
Gr-1
31.9%
30.1%
19.0%
55.6%
p=0.0003
c-Kit+ Gr-1- (%)
p=0.0157
Mac-1+ Gr-1+ (%)
G
ns
cells in S/G2/M (%)
Apoptotic cells (%)
H
AML#1 cells
i.v. injection
NRGS
Bone marrow aspiration
stop
14 21
I
MMF
J
% of human CD45+ cells in bone marrow
K
NSG
Or
C57BL/6J
NSG-Vehicle
NSG-FF-10501-01
C57BL/6J-Vehicle
C57BL/6J-FF-10501-01
p=0.0275

Figure 2.

of these mice (Fig 2I and J; Appendix Fig S5A and B). These data suggest that the lack of an intact immune system in NRGS mice may limit the therapeutic effect of IMPDH inhibitors in this PDX model. Indeed, we found that FF-10501-01 treatment provided more survival benefits in immunocompetent C57BL/6J mice than immunodeficient NOD/SCID-IL2R$\gamma^{-/-}$ (NSG) mice even in the mouse MLL-AF9 model (Fig 2K), indicating that systemic immune responses contribute to the *in vivo* effect of IMPDH inhibition.

Because IMPDH inhibitors are known to act as immunosuppressants, we next assessed whether our dosing schedule for FF-10501-01 affects the numbers of immune cells in leukemic mice. We treated the MLL-AF9-bearing mice with FF-10501-01 every other day from Day 1 to Day 9 and then examined the numbers and frequency of immune cells in bone marrow and spleen at Day 10. In agreement with earlier results, treatment with FF-10501-01 substantially inhibited the growth of MLL-AF9 cells. Although FF-10501-01 treatment modestly decreased the total numbers of normal hematopoietic cells (Appendix Fig S6A and B), it did not reduce the frequency of B and T lymphocytes, NK cells, macrophages, and neutrophils in the bone marrow and spleen (Appendix Fig S6C and D). Thus, our alternate-day treatment with IMPDH inhibitors did not eradicate immune cells in mice, and the remaining immune cells are likely to enhance the antileukemia effect of IMPDH inhibitors *in vivo*.

Taken together, these results suggest that IMPDH inhibitors suppress the *in vivo* development of MLL-AF9-driven AML with the assistance of immune cells.

p53-p21 activation is dispensable for the antileukemia effect of IMPDH inhibitors

Several studies have proposed that activation of the p53-p21 pathway contributes to the antitumor effect of IMPDH inhibitors (Messina *et al*, 2004; Sun *et al*, 2008). Indeed, we observed consistent upregulation of p53 in CB cells, RUNX1-ETO-expressing CB cells (preleukemic cells; Goyama *et al*, 2016), and two MLL-AF9-expressing CB cells treated with MPA. The expression of p21 was also upregulated in most of these cells, except for an MLL-AF9 leukemia clone, CB-MLL-AF9#1 (Fig 3A). The p53-p21 upregulation was reversed by simultaneous addition of guanosine, indicating that this was an on-target effect of MPA. To examine the role of p21 in the regulation of MPA's efficacy, we depleted p21 in CB-MLL-AF9#2 using a p21-targeting shRNA (10; Fig 3B). MPA inhibited the growth of p21-depleted MLL-AF9 cells as efficiently as control cells (Fig 3C). These data, together with the fact that CB-MLL-AF9#1 lacking p21 expression is sensitive to MPA, indicate that p21 is dispensable for the growth-inhibitory effect of IMPDH inhibition.

We next examined whether p53 activation is necessary for the antileukemia effect of MPA. We transduced vector or a dominant-negative p53 fragment (p53DD) into MLL-AF9-expressing CB cells (Fig 3D) and assessed the effect of MPA on these cells *in vitro*. MLL-AF9 cells transduced with p53DD were equally sensitive to MPA relative to control cells (Fig 3E). Finally, we assessed the sensitivity of p53-deficient mouse MLL-AF9 leukemia cells to IMPDH inhibition *in vivo*. We generated p53-deficient leukemia cells by expressing MLL-AF9 into bone marrow progenitors derived from p53 knockout mice (Tamura *et al*, 2019). The p53-deficient MLL-AF9 cells were transplanted into recipient mice, and the mice were treated with vehicle or IMPDH inhibitors. The p53-MDM2 interaction inhibitor DS-5272 (Hayashi *et al*, 2019) was also used as a control (Fig 3G). Consistent with the *in vitro* data, treatment with FF-10501-01 induced p53 upregulation in MLL-AF9 cells *in vivo*, which was not observed in p53-deficient MLL-AF9 cells (Fig 3F). Again, we found that p53-deficient MLL-AF9 cells were still sensitive to FF-10501-01 treatment *in vivo* while resistant to DS-5272 (Fig 3H). Taken together, we concluded that activation of the p53-p21 pathway is not essential for the antileukemia efficacy of IMPDH inhibitors.

IMPDH inhibition induces inflammation and alters metabolic homeostasis *in vivo*

To assess the molecular changes induced by IMPDH inhibition in MLL-AF9 cells, we first examined expression profiles of vehicle- or FF-10501-01-treated MLL-AF9 cells after two consecutive days of treatment *in vivo* (Fig 4A). A clear separation of FF-10501-01-treated cells and controls was observed (Fig 4B and C). Gene set enrichment analysis (GSEA; Mootha *et al*, 2003; Subramanian

Figure 3. The therapeutic effect of IMPDH inhibition on AML is p53-p21 independent.

A Immunoblotting of the cord blood cells and those expressing RUNX1-ETO or MLL-AF9. The cells were incubated with/without 1 μM MPA (M) and 100 μM Guanosine (G) for 24 h.

B CB-MLL-AF9#2 cells were transduced with a Non-targeting (NT) control and an shRNA targeting p21 (sh-p21). Cells were incubated with/without 1 μM MPA (M) and 100 μM guanosine (G) for 24 h. Total cell lysates were analyzed by western blotting using the antibodies for p21 and Tubulin.

C CB-MLL-AF9#2 cells transduced with NT or sh-p21 shRNAs were incubated with MPA at the indicated concentration for 72 h. Cell viability assays were performed using WST-1 in three technical replicates.

D CB-MLL-AF9#2 cells were transduced by vector control or p53DD (dominant-negative) and were incubated with/without 1 μM MPA (M) and 100 μM guanosine (G) for 24 h. Total cell lysates were analyzed by western blotting using the antibodies for p53 and Tubulin. Note the elevated levels of endogenous p53 protein (upper arrow) in p53DD (lower arrow)-expressing cells.

E Cells were incubated with MPA at the indicated concentration with/without 100 μM guanosine for 72 h then measured with WST-1 in three technical replicates.

F Mouse bone marrow c-Kit^{+} cells derived from Trp53$^{-/-}$ mice were transduced with MLL-AF9-GFP and were transplanted into mice to generate p53-deficient (p53$^{-/-}$) leukemia cells. GFP^{+} MLL-AF9 leukemia cells were collected from bone marrows of vehicle- or FF-10501-01-treated wild-type and p53$^{-/-}$ leukemic mice. Expression levels of p53 and Tubulin were assessed by western blotting 24 h after the treatment.

G Experimental scheme used in (H). The p53-deficient MLL-AF9-bearing mice were treated with vehicle, FF-10501-01 or DS-5272 every other day from day 1.

H Kaplan–Meier survival curves of p53$^{-/-}$ MLL-AF9 leukemia mice treated with vehicle or FF-10501-01 or DS-5272 (n = 6 per group). Statistical significance was evaluated by the log-rank test (left). Frequency of GFP^{+} leukemia cells in peripheral blood at day 19 (right, n = 6 per group). A two-tailed unpaired t-test was used for the comparison.

Data information: All data are shown as mean ± SEM.
Source data are available online for this figure.

 ©2022 The Authors

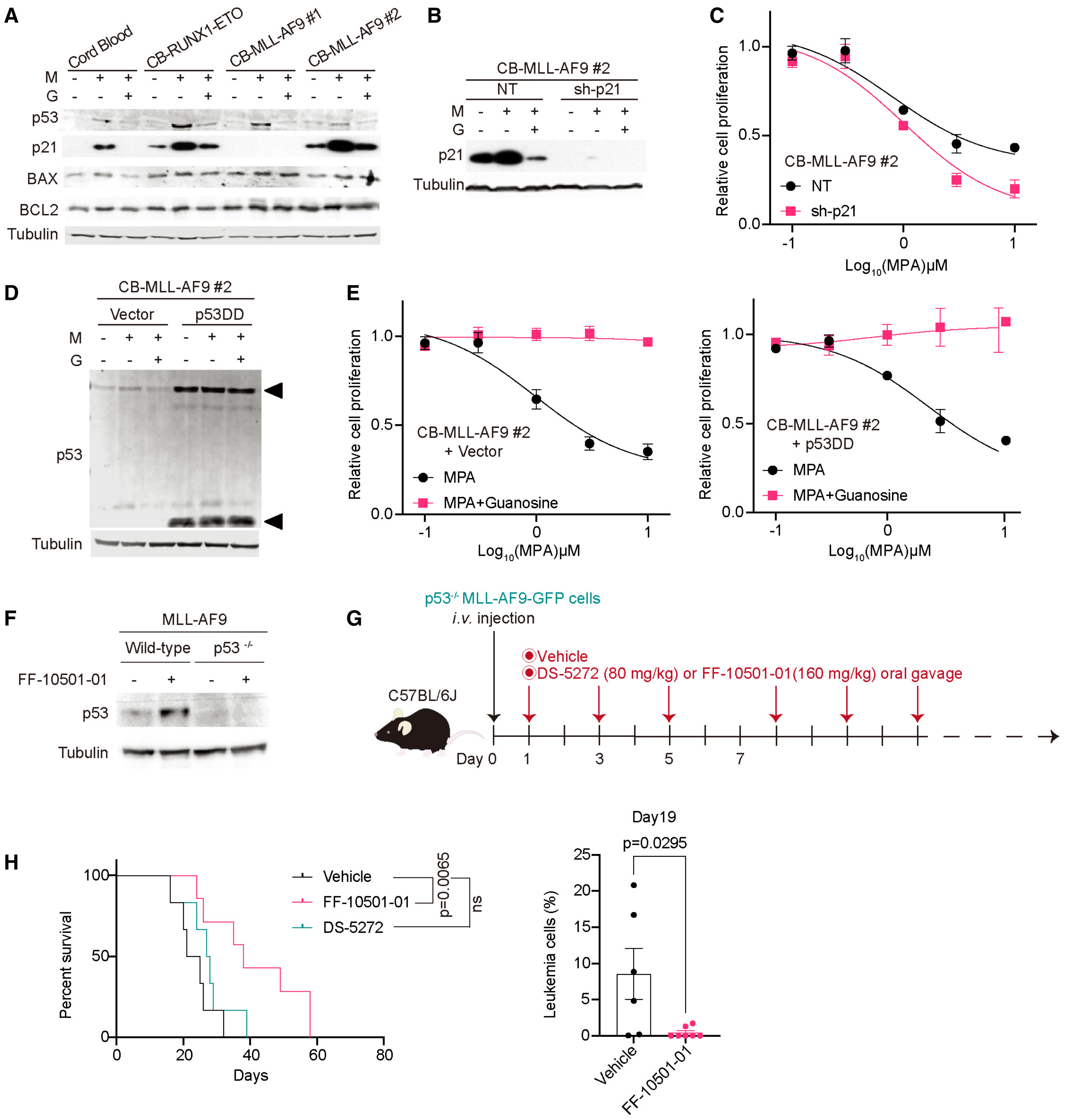

Figure 3.

et al, 2005) revealed upregulation of inflammation- and interferon-associated genes, suggesting that IMPDH inhibition triggers an immune-inflammatory response in MLL-AF9 cells (Fig 4D). IMPDH inhibition also induced downregulation of mTORC1 signaling (Fig 4E), which is consistent with a previous report (Emmanuel *et al*, 2017). Among the mTORC1 pathway molecules, we found that *SLC7A5* and *SLC3A2* were downregulated in FF-10501-01-treated MLL-AF9 cells (Fig 4F). *SLC7A5* and *SLC3A2* encode CD98 and Lat1, respectively, and they form a heterodimeric membrane complex that is involved in the uptake of essential amino acids and adhesive signals (Feral *et al*, 2005; Hafliger & Charles, 2019). We confirmed reduced surface expression of CD98/Lat1 in mouse MLL-AF9 cells *in vivo* (Fig 4G), and the reduction was also confirmed in human MOLM13 cells and MV4;11 cells treated with

©2022 The Authors

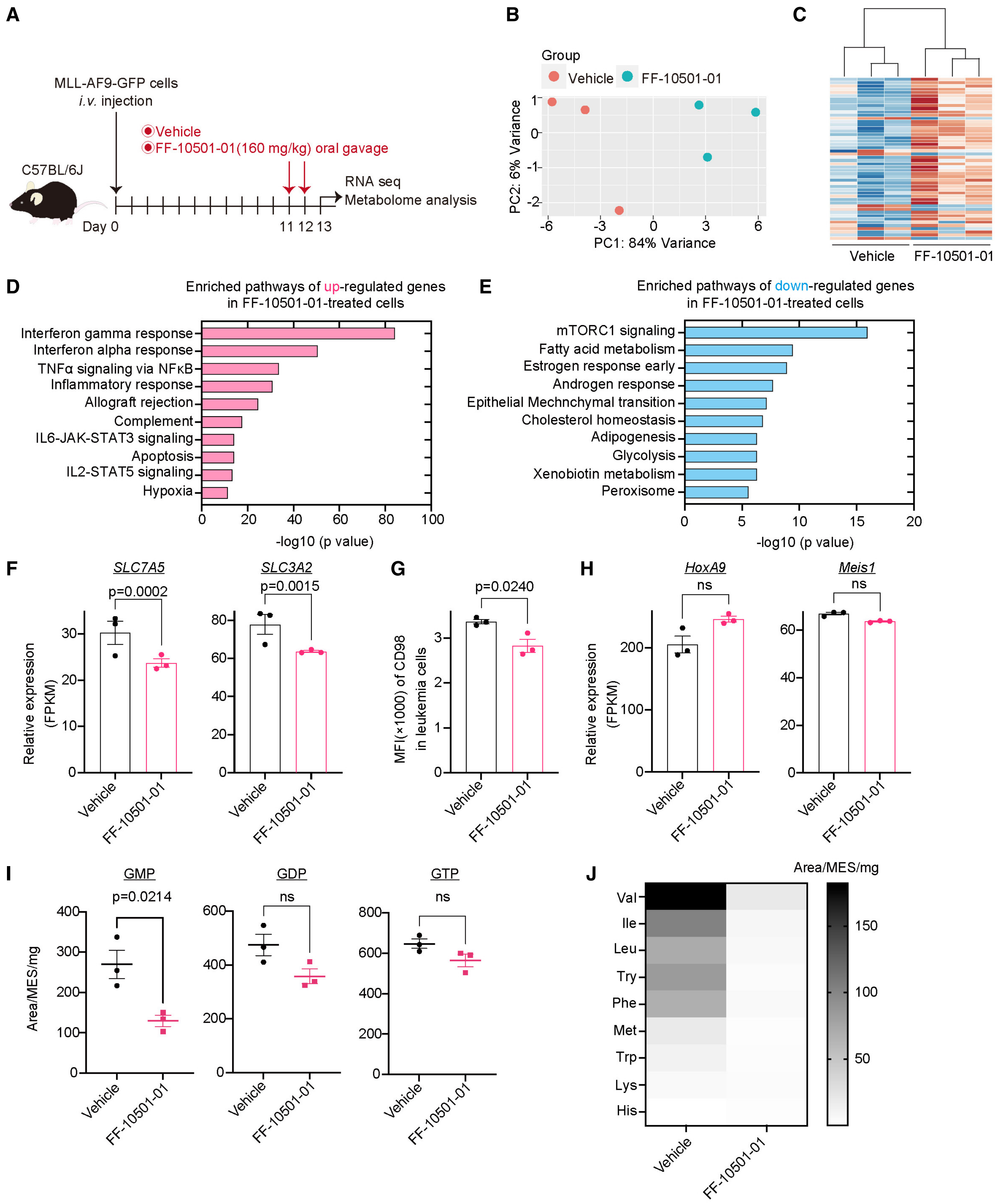

Figure 4.

©2022 The Authors

Figure 4. The effect of IMPDH inhibition on gene expression and metabolic profiles.

A Experimental scheme used in (B-J). C57BL/6J mice were transplanted with MLL-AF9-GFP cells and were treated with vehicle or FF-10501-01 on day 11 and 12. GFP^{+} MLL-AF9 leukemia cells collected from mice were used for RNA-seq and metabolome analysis at day 13.

B, C Principal Component Analysis (B) and hierarchical clustering (C) of RNA-Seq data. $n = 3$ per group.

D, E GSEA for up- (D) and down- (E) regulated genes in FF-10501-01-treated MLL-AF9 cells using the Hallmark collections of the GSEA MSigDB (http://software.broadinstitute.org/gsea/msigdb). The x-axis shows the *P*-value (−log10).

F mRNA expression (FPKM) of *SLC7A5* and *SLC3A2* in vehicle- or FF-10501-01-treated leukemia cells. The *P*-value was calculated by Cuffdiff. $n = 3$ (biological replicates) for each group.

G MFI of CD98 on the vehicle- or FF-10501-01-treated leukemia cells in mice. A two-tailed unpaired *t*-test was used for the comparison. $n = 3$ (biological replicates) for each group.

H mRNA expression (FPKM) of *HoxA9* and *Meis1* in vehicle- or FF-10501-01-treated leukemic cells. *P*-value was calculated by Cuffdiff. $n = 3$ (biological replicates) for each group.

I, J Vehicle- or FF-10501-01-treated leukemic cells were used for metabolome analyses. $n = 3$ (biological replicates) per group. Levels of guanine nucleotides (GMP, GDP, and GTP) (I) and amino acid contents (J) are shown. Data are presented as mean ± SEM (I) or as a viridis heatmap (Plot is SEM) (J). A two-tailed unpaired *t*-test was used for the comparison.

Data information: All data are shown as mean ± SEM.

FF-10501-01 *in vitro* (Fig EV4). Given the important role of CD98 in AML progression (Bajaj *et al*, 2016), the CD98/Lat1 downregulation may contribute to the therapeutic effect of IMPDH inhibitors on AML. By contrast, FF-10501-01 treatment did not affect the expression of MLL-AF9 target genes, such as *HoxA9* and *Meis1* (Fig 4H), indicating the little effect of IMPDH inhibition on the aberrant epigenetic programs induced by MLL-AF9.

We then performed metabolomic analyses using MLL-AF9 cells collected from leukemic mice treated with vehicle or FF-10501-01. As expected, FF-10501-01 treatment tended to reduce intracellular levels of guanine nucleotides in MLL-AF9 cells, although the levels of GDP/GTP were not significantly downregulated probably due to the *in vivo* compensatory mechanisms (Fig 4I). Interestingly, levels of other nucleoside monophosphate (AMP, CMP, and UMP) were also downregulated upon FF-10501-01 treatment, indicating the presence of crosstalk between each nucleotide (Appendix Fig S7). We also found reduced levels of several essential amino acids (EAAs), including valine, isoleucine, leucine, tyrosine, and phenylalanine in FF-10501-01-treated MLL-AF9 cells (Fig 4J). Given that Lat1 preferentially transports these amino acids, the Lat1 downregulation upon IMPDH inhibition may be responsible for the reduction in EAAs. In addition, FF-10501-01 treatment of MLL-AF9 cells resulted in a wide range of alterations in the levels of bases and amino acids (Appendix Fig S8). Thus, IMPDH inhibition *in vivo* provokes inflammatory responses and metabolic alterations in MLL-AF9 AML cells.

IMPDH inhibition induces overactivation of TLR signaling in AML cells

Previous reports have shown that IMPDH suppresses TLR2-mediated NF-κB activation (Toubiana *et al*, 2011) and TLR-7-mediated antiviral activity (Lee *et al*, 2006). Therefore, we speculated that activation of TLR signaling could underlie the increased inflammation in MLL-AF9 cells treated with IMPDH inhibitors. To test this hypothesis, we first assessed the effect of MPA on TLR signaling pathways using a mouse pro-B cell line Ba/F3 that expresses individual TLRs and the NF-κB -GFP reporter (Matsumoto *et al*, 2008; Shibata *et al*, 2012; Sato *et al*, 2017; Fig 5A). The addition of MPA increased TLR2-, TLR4-, TLR5-, and TLR7/8-mediated activation of NF-κB in a dose-dependent manner (Fig 5B), indicating the inhibitory role of IMPDH in the TLR-NF-κB pathway. Next, we assessed the role of IMPDH in the regulation of TRAF6, an E3 ubiquitin ligase that plays a pivotal role in linking TLR signaling to NF-κB. We introduced FLAG-tagged TRAF6 and HA-tagged K63-ubiquitin into 293 T cells. After 24 h, these cells were treated with MPA with/without guanosine. Cell lysates were subjected to immunoprecipitation with FLAG antibody for TRAF6, followed by immunoblotting with anti-HA to detect ubiquitinated TRAF6. We found that MPA treatment increased TRAF6 autoubiquitination (an active marker of TRAF6), which was reversed by the supplementation of guanosine (Fig 5C). Thus, IMPDH inhibition induces activation of the TLR-TRAF6-NF-κB signaling pathway.

Figure 5. IMPDH inhibition leads to overactivation of the TLR-TRAF6-NF-κB pathway.

A Experimental scheme used in (B). Ba/F3 cells were transduced with NF-κB-GFP reporter and were then transduced with individual mouse TLR4, TLR5, or TLR7 to establish BaκB cells expressing each TLR. TLR2 is expressed in parent Ba/F3 cells.

B The TLR-expressing BaκB cells were stimulated with the corresponding TLR ligands (100 ng/ml Pam3CSK4 for TLR2, 100 ng/ml LPS for TLR4, 10 ng/ml Flagellin for TLR5, 100 ng/ml R848 for TLR7) with titrating doses of MPA (1–10 μM). GFP expression was assessed 24 h after the stimulation.

C 293 T cells were transfected with FLAG-tagged TRAF6 and HA-tagged K63-ubiquitin. After 24 h, these cells were treated with 3 or 10 μM MPA (M-3 μM or M-10 μM) with/without 100 μM guanosine (G). IRAK1/4 inhibitor (IRAK1/4 i) was also used as a control. Whole-cell extracts were immunoprecipitated with anti-FLAG antibody, and ubiquitinated TRAF6 was detected with anti-HA antibody.

D, E Murine bone marrow-derived macrophages (BMDMs) were treated with vehicle, 10 μM MPA alone (D) or co-treated with vehicle, 10 μM MPA (M), 100 ng/ml Pam3CSK4 and 100 μM Guanosine (G) for 4 h (E), as indicated. $N = 3$ (technical replicates) per group. mRNA levels of *TNF-α* and *IL-1β* were measured by qPCR. Two-tailed unpaired *t*-tests were used for the comparison in (D). Ordinary one-way ANOVA was used for the comparison in (E).

F, G (F) THP1 cells and (G) MLL-AF9-expressing CB cells (CB-MLL-AF9#1) were pretreated with 10 μM MPA for 2 h and then treated with 1,000 ng/ml Pam3CSK4 for 0, 30, 60, and 120 min. Cells were lysed and subjected to SDS/PAGE. Levels of total IκBα, phosphate-p38, total p38, and GAPDH were evaluated by western blotting.

Data information: All data are shown as mean ± SEM.
Source data are available online for this figure.

©2022 The Authors

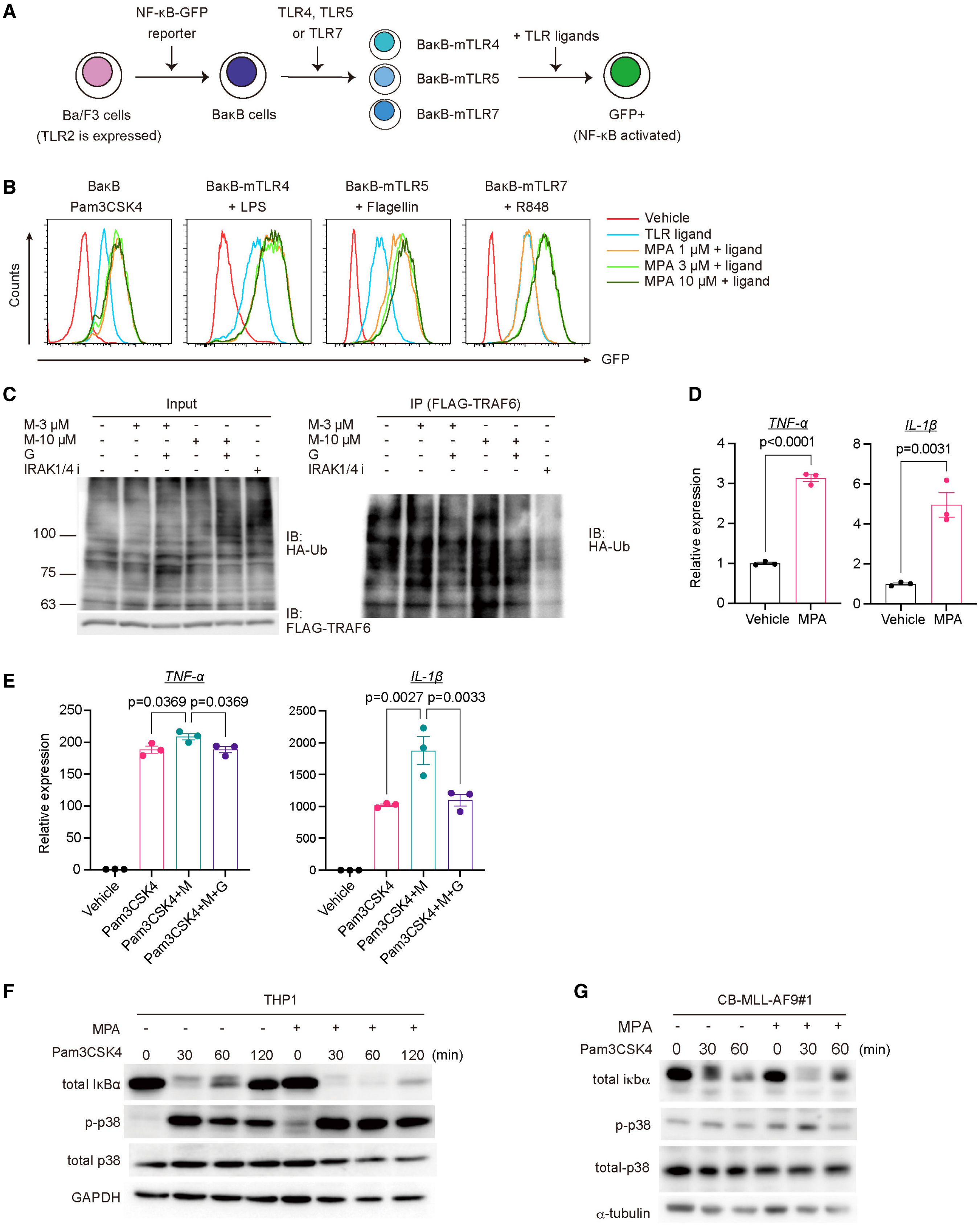

Figure 5.

©2022 The Authors

We then examined the effect of MPA on induction of pro-inflammatory cytokines. Mouse bone marrow-derived macrophages (BMDMs) were treated with MPA, guanosine, and the TLR1/2 agonist Pam3CSK4 for 4 h, and levels of *TNF-α* and *IL-1β* were assessed. Mycophenolic acid treatment modestly increased *TNF-α* and *IL-1β* expression (Fig 5D) and enhanced Pam3CSK4-induced upregulation of these pro-inflammatory cytokines in BMDMs (Fig 5E). The collaborative effect of MPA and Pam3CSK4 was abrogated with the supplementation of guanosine, indicating that the reduction in guanine nucleotides triggered the inflammatory responses (Fig 5E). We also found that MPA treatment increased IκBα degradation and p38 phosphorylation induced by Pam3CSK4 in THP1 cells and CB cells expressing MLL-AF9 (Fig 5F and G). Collectively, these data suggest that IMPDH inhibition in fact promotes TLR signaling, which induces excessive inflammation in AML cells.

Vcam1 regulates cell–cell interaction and suppresses proliferation in MLL-AF9 cells

To further assess the molecular alterations induced by IMPDH inhibition, we next performed single-cell mass cytometry analyses using MLL-AF9 cells collected from mice treated with vehicle or FF-10501-01(Fig 6A). Four populations were identified according to Mac-1, Gr-1, and c-Kit expression on the Spanning-tree Progression Analysis of Density-normalized Events (SPADE) tree (Fig 6B; Appendix Fig S9A and B). We found that FF-10501-01 induced Vcam1 upregulation in Mac-1$^+$ fraction, phospho-STAT1 upregulation in Mac-1$^+$c-Kit$^+$ fraction, CXCR4 downregulation in c-Kit$^+$ fraction and HIF-1α upregulation in Gr-1$^+$ fraction in MLL-AF9 cells (Fig 6C; Appendix Fig S9C). Flow cytometry analysis also confirmed the Vcam1 upregulation in FF-10501-01-treated leukemia cells (Fig 6D). Guanosine supplementation reversed the FF-10501-01-induced Vcam1 upregulation only partially (Appendix Fig S10A), indicating that Vcam1 upregulation in MLL-AF9 cells was induced by both direct and indirect (probably related to enhanced inflammation and myeloid differentiation) effects of IMPDH inhibition. Because Vcam1 is known to be activated by pro-inflammatory cytokines (Kong *et al*, 2018), the overactive TLR signaling in FF-10501-01-treated MLL-AF9 cells could also contribute to Vcam1 upregulation. To assess the role of Vcam1 in the development of MLL-AF9 leukemia, we then transduced Cas9 together with a vector or two independent Vcam1-targeting single guide RNAs (sgRNAs) into mouse MLL-AF9 cells. Vcam1-targeting sgRNAs induced nearly complete depletion of Vcam1 on the cell surface of MLL-AF9 cells. Interestingly, Vcam1-depleted MLL-AF9 cells lost cell–cell contacts and stopped forming cell aggregates in suspension cultures (Fig 6E). Conversely, Vcam1 overexpression in MLL-AF9 cells using SunTag system (Tanenbaum *et al*, 2014) markedly increased the formation of cell aggregation in culture (Fig 6G). We also found that *Vcam1* depletion promoted cell cycle progression (Fig 6F), whereas Vcam1 overexpression inhibited it in MLL-AF9 cells (Fig 6H). Depletion of Vcam1 showed little effects on FF-10501-01-induced myeloid differentiation in MLL-AF9 cells (Appendix Fig S10B).

To determine the role of Vcam1 in AML progression *in vivo*, we next transduced tRFP657-co-expressing non-targeting or Vcam1-targeting sgRNAs into mouse MLL-AF9/Cas9 cells and transplanted these cells into recipient mice (Fig 6I). Vcam1 depletion promoted the leukemic progression of MLL-AF9 cells, as evidenced by the increase of Vcam1-depleted (tRFP657$^+$) cells in the bone marrow of the mice (Fig 6J). Taken together, these data suggest that Vcam1 promotes excessive cell–cell interaction of AML cells, which prevents their efficient proliferation both *in vitro* and *in vivo*.

Co-treatment with IMPDH inhibitors and TLR1/2 agonist shows strong antileukemia effects

Because the TLR1/TLR2 agonist Pam3CSK4 was shown to inhibit MLL-AF9-driven leukemogenesis (Eriksson *et al*, 2017), we next assessed the combined therapeutic effect of MPA and Pam3CSK4 on human MLL-AF9-expressing CB cells and the *in vivo* mouse AML model driven by MLL-AF9. Consistent with earlier results and the previous report (Eriksson *et al*, 2017), both MPA and Pam3CSK4 induced upregulation of myeloid markers CD11b and CD14 in MLL-

Figure 6. Vcam1 regulates cell–cell interaction and inhibits proliferation in MLL-AF9 cells.

A Experimental scheme used in (B and C). CD45.2$^+$ MLL-AF9 cells were collected from CD45.1$^+$ recipient mice treated with the vehicle- (n = 4) or FF-10501-01 (n = 4) and were subjected to mass cytometry analysis.

B CD45.2$^+$ fraction was used for SPADE analysis in Cytobank (https://premium.cytobank.org/cytobank/login). The SPADE trees show each marker's median expression (From the red to the blue color indicated high to low expression). The size of the node is proportional to the size of the cell population (Min is 20, Max is 33627.3 cell). Several distinct cell populations (a, b, c, d) are highlighted according to the expression of Mac-1, c-Kit, and Gr-1.

C Vcam1 expression in vehicle or FF-10501-01-treated cells is shown.

D Frequencies of Vcam1$^+$ cells in MLL-AF9 cells collected from the vehicle (n = 6) or FF-10501-01(n = 6) treated mice were assessed by FCM. Two-tailed unpaired t-test was used for the comparison.

E Left: levels of Vcam1 expression in MLL-AF9-Cas9 cells transduced with vector or two independent Vcam1-targeting sgRNAs (KO1 and KO2). Right: the appearance of cells under microscope's bright field in suspension cultures. (Scale bar: 125 μm).

F Cell cycle status of control and Vcam1-depleted MLL-AF9 cells. Left: representative histograms. Right: bar graphs showing the ratio of G0/G1- and S/G2/M-phase cells are shown.

G Left: levels of Vcam1 expression in MLL-AF9-dCas9-SunTag cells transduced with non-target or sgRNAs targeting Vcam1 promoter regions (ac1, ac2, ac3). Right: the appearance of cells under visual field in suspension cultures in the 12 well plates.

H Cell cycle status of control and Vcam1- overexpressed MLL-AF9 cells. Left: representative histograms. Right: bar graphs showing the ratio of G0/G1- and S/G2/M-phase cells are shown.

I Experimental scheme used in (J). MLL-AF9-Cas9 cells were transduced with non-target and Vcam1-targeting sgRNAs (tRFP657$^+$) following transplantation into recipient mice.

J Relative ratios of the change of tRFP657+ (sgRNA-transduced) fraction in GFP$^+$ MLL-AF9 leukemia cells before and after transplantation are shown. Ordinary one-way ANOVA was used for the comparison. n = 8 for Non-target, n = 9 for vcam1-KO1, n = 8 for vcam1-KO2. Biological replicates.

Data information: All data are shown as mean ± SEM.

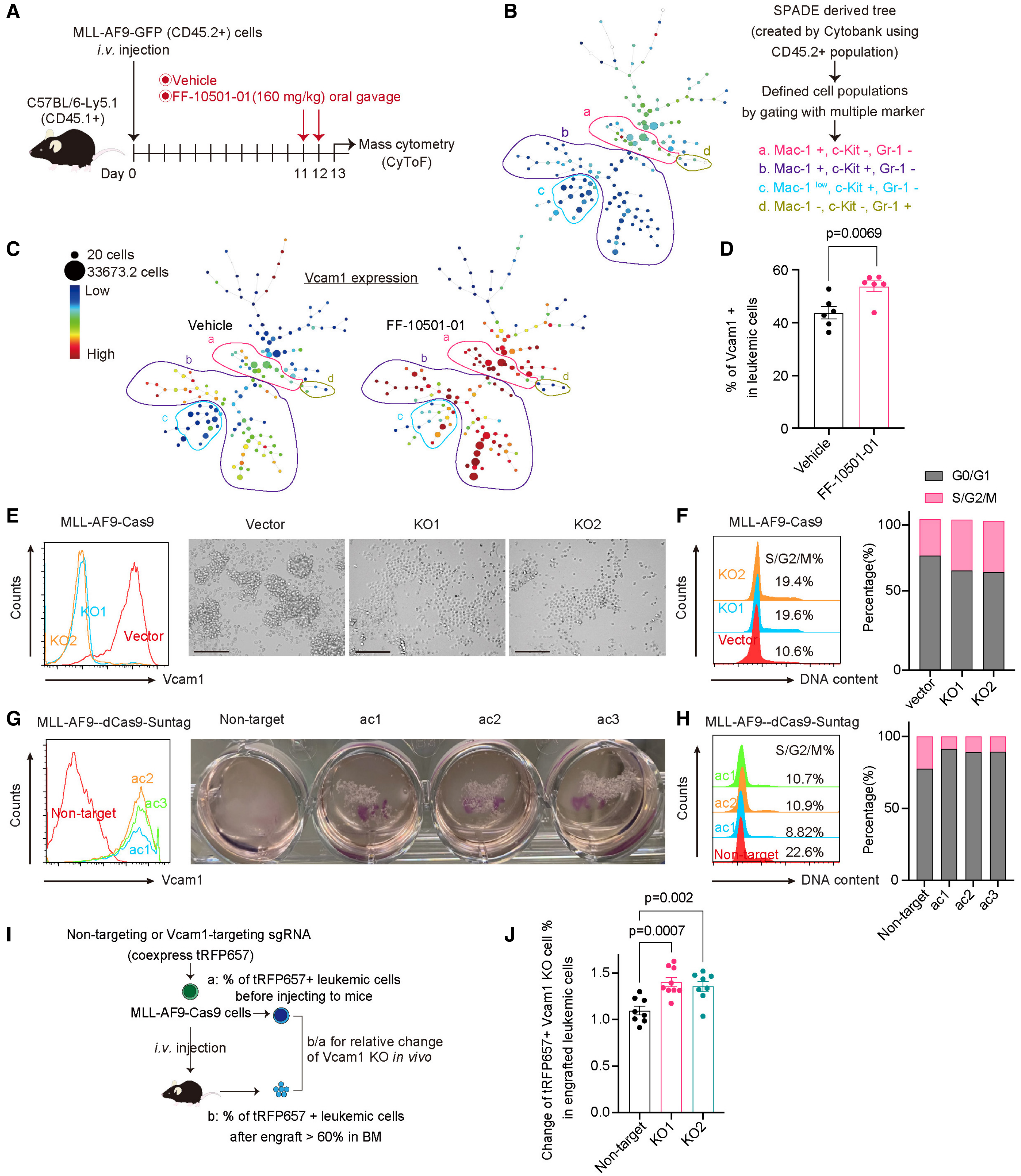

Figure 6.

AF9 cells *in vitro*, and cotreatment with these drugs strongly promoted differentiation of MLL-AF9 cells toward macrophage (Fig 7A and B). Furthermore, cotreatment with Pam3CSK4 and MPA-induced Vcam1 upregulation in MLL-AF9 cells, which probably contributes to their antileukemia effect (Fig 7C). We then assessed the *in vivo* effects of FF-10501-01 (160 mg/kg) and Pam3CSK4

(8 μg/mouse) on MLL-AF9-induced leukemogenesis (Fig 7D). Although Pam3CSK4 alone did not inhibit the leukemic progression of MLL-AF9 cells, combined treatment of FF-10501-01 and Pam3CSK4 significantly prolonged the survival of leukemia mice than treatment with either FF-10501-01 or Pam3CSK4 alone (Fig 7E and F). These findings suggest that co-treatment with IMPDH inhibitors and TLR1/2 agonist could be a promising therapeutic strategy for MLL-fusion AMLs.

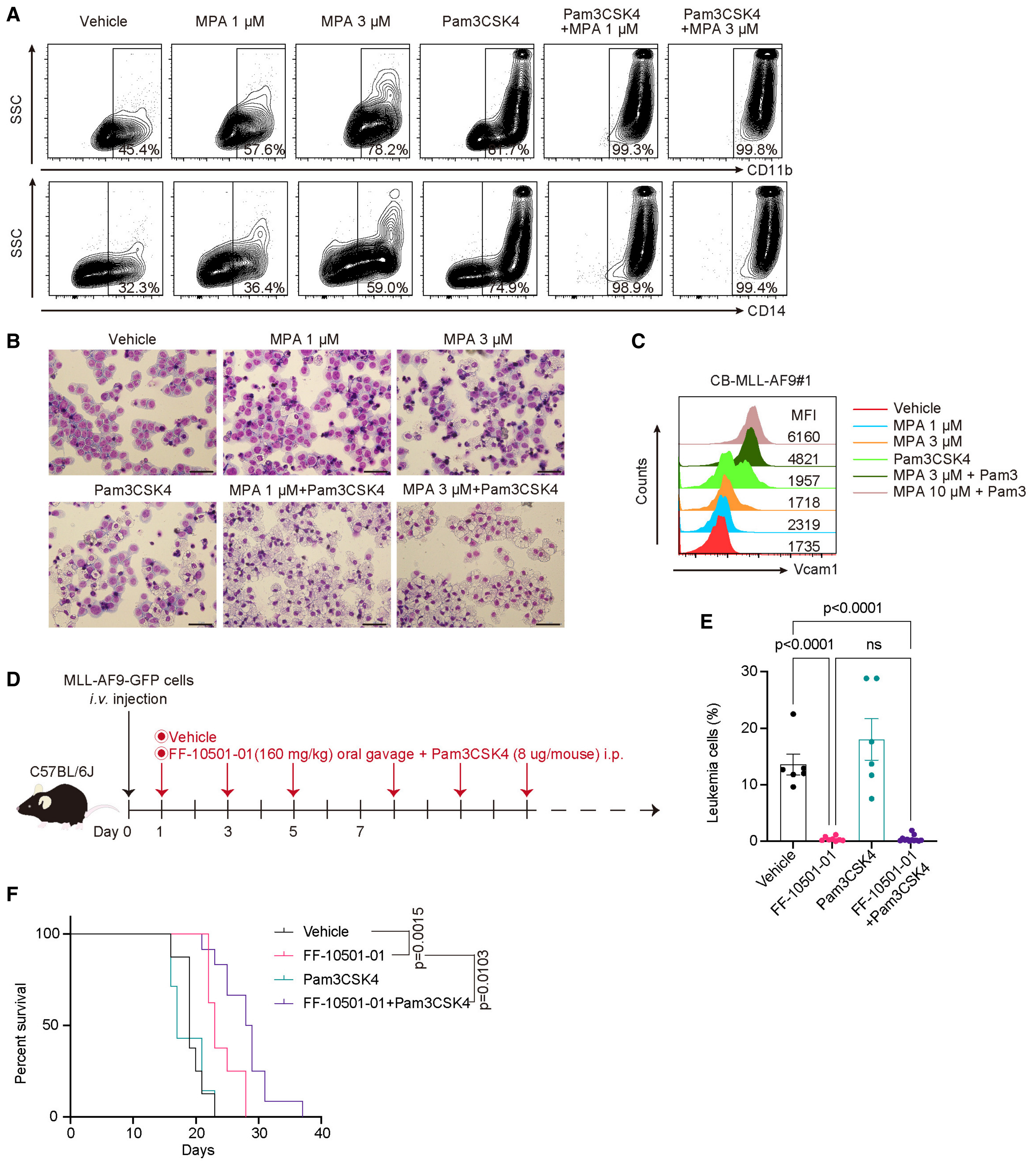

Figure 7.

Figure 7. Co-treatment with MPA and Pam3CDK4 induces differentiation of AML cells.

A–C MLL-AF9-expressing cord blood cells (CB-MLL-AF9#1) were treated with vehicle, 100 ng/ml Pam3CSK4, and 1 or 3 μM MPA for 72 h. (A) Levels of CD11b and CD14, (B) Wright-Giemsa staining of (A) (Scale bar: 50 μm), and (C) Vcam1 expression in the CB-MLL-AF9#1 were assessed. The numbers shown in (A) indicate the frequency of $CD11b^+$ or $CD14^+$ cells. The numbers shown in (C) are Median Fluorescence Intensity (MFI) of Vcam1.

D Experimental scheme used in (E and F). C57BL/6J were transplanted with 1×10^5 MLL-AF9 cells and were then treated with vehicle, FF-10501-01(160 mg/kg, oral), Pam3CSK4 (8 μg/mouse, i.p.) or FF-10501-01 + Pam3CSK4 from day 3. Drugs were given to mice every other day.

E Frequencies of GFP^+ leukemic cells in peripheral blood at day 16 or day 20 are shown. Vehicle: $n = 7$ mice/group; FF-10501-01 only: $n = 8$; Pam3CSK4 only: $n = 7$; FF-10501-01 + PamCSK4: $n = 12$. Data are shown as mean ± SEM from 2 independent experiments. Ordinary one-way ANOVA was used for the comparison.

F Kaplan–Meier survival curves of MLL-AF9 leukemia mice treated with vehicle ($n = 8$), FF-10501-01 ($n = 8$), Pam3CSK4 ($n = 7$) or FF-10501-01 + Pam3CSK4 ($n = 12$). Statistical significance was calculated by the log-rank test.

Data information: All data are shown as mean ± SEM.

Discussion

Although IMPDH has been considered as a potential anticancer drug target, the therapeutic effect of IMPDH inhibitors has not been proven in the clinical setting. It is therefore important to determine the types of tumors that are susceptible to IMPDH inhibitors and to establish optimal dose and treatment schedule to maximize their effects. In this study, we identified MLL-fusion leukemias as sensitive tumors to IMPDH inhibition. Furthermore, we showed that alternate-day administration of MPA and FF-10501-01 to mice effectively suppressed MLL-AF9-driven leukemogenesis without having a devastating effect on immune function. The clinical activity of IMPDH inhibitors to MLL-fusion leukemias warrants further investigation in clinical trials.

Mechanisms underlying the anticancer activity of IMPDH inhibitors have been investigated for many years. Although it has been shown that IMPDH inhibition activates the p53–p21 pathway (Messina *et al*, 2004; Sun *et al*, 2008), our current and previous (Kofuji *et al*, 2019) data indicate that this pathway is not essential for the anticancer effect of IMPDH inhibitors. Instead, we found that IMPDH inhibitors provoke overactivation of TLR-TRAF6-NF-κB signaling, which induces differentiation of AML cells. Because the MPA-induced activation of TLR-TRAF6-NF-κB signaling was reversed by guanosine supplementation, the reduced level of guanine nucleotides is likely to be triggers for the activation of this pathway. Given that most MLL-fusion AMLs have monocytic and macrophage-like characteristics and the TLR signaling plays key roles in monocytes/macrophages, the active TLR signaling in MLL-fusion AMLs could explain why they are sensitive to IMPDH inhibitors. We also found the strong antileukemia effects of the combined treatment with IMPDH inhibitors and TLR1/2 agonist on MLL-AF9-driven AML. Taken together, we propose that tumors with active TLR signaling, not limited to MLL-fusion AMLs, would respond well to IMPDH inhibitors. Furthermore, given the importance of TLR signaling in innate immune cells, our findings raise the possibility that IMPDH may have an important role in regulating the balance between innate and adaptive immunity. These hypotheses need to be verified in future research.

VCAM1 is a key cell adhesion molecule involved in inflammation (Kong *et al*, 2018). Although VCAM1 expression is low in most primary AML cells and AML cell lines, IMPDH inhibition induced Vcam1 upregulation in MLL-AF9-AML cells. Moreover, our functional analyses revealed the critical role of Vcam1 to regulate cell–cell interaction among AML cells, which prevents their leukemic proliferation. These data suggest that Vcam1 acts as a tumor suppressor and contributes to the antileukemia effect of IMPDH inhibitors on MLL-fusion AML. In contrast to our findings, Pinho *et al* (2022) recently showed that VCAM1 confers innate immune tolerance on leukemia stem cells, thereby promoting leukemogenesis driven by MLL-AF9 (Pinho *et al*, 2022). The seemingly contradictory observations could be explained by the differences in the timing to induce Vcam1 depletion and the context of major histocompatibility complex (MHC) class-I presentation. We induced Vcam1 depletion in established MLL-AF9 cells and used syngeneic mice as recipients, while Pinho *et al* (2022) expressed MLL-AF9 in bone marrow cells derived from Vcam1-deficient mice and used MHC-mismatched mice as recipients. Thus, it appears that Vcam1 has diverse and context-dependent roles in the initiation, progression, and maintenance of AML, which remains to be clarified in future studies.

The antileukemia effect of IMPDH inhibitors could be augmented by the combination with other drugs. We previously showed that azacitidine-resistant leukemia cells that acquire resistance to a hypomethylating agent Azacitidine were still sensitive to IMPDH inhibitors (Murase *et al*, 2016), indicating that they could be combined with hypomethylating agents to treat myeloid neoplasms. However, cotreatment with MPA and decitabine did not show synergistic effects on human CB cells expressing MLL-AF9. Instead, we found that MPA synergized with a BCL2 inhibitor (Venetoclax) to inhibit the growth of MLL-AF9 cells (Appendix Fig S3). Thus, combined treatment with IMPDH and BCL2 inhibitors could be promising frontline therapies for MLL-fusion leukemia.

In summary, we show the therapeutic potential of IMPDH inhibitors for MLL-fusion leukemia. Alternate-day administration of IMPDH inhibitors to mice suppresses the development of MLL-AF9-driven AML *in vivo* without deteriorating the immune system. The *in vitro* and *in vivo* efficacy strongly support future research to maximize the impact of IMPDH inhibitors on MLL-fusion AML and potentially other hematopoietic neoplasms.

Materials and Methods

Human cord blood cells and patient samples

Umbilical cord blood units were obtained from the Translational Trials Development Support Laboratory of Cincinnati Children's Hospital Research Foundation or the Japanese Red Cross Kanto-Koshinetsu Cord Blood Bank (Tokyo, Japan). Proper informed consent was obtained, and all experiments were performed

 ©2022 The Authors

according to an institutional review board-approved protocol (approval number: 27-34-1225), in accordance with the Declaration of Helsinki and The Belmont Report. Residual diagnostic specimens from AML or ALL patients at Cincinnati Children's Hospital Medical Center (CCHMC) were treated with OKT3 antibody and engrafted into NSG or NSGS mice (Wunderlich *et al*, 2014). The engrafted human AML and ALL cells were then collected from the bone marrow of the recipient mice and used for the experiments.

Mice and animal procedures

C57BL/6JJmsSlc mice were purchased from Japan SLC, Inc. and used for experiments at the age of 8–12 weeks. Ly5.1 mice were maintained in IRCMS, Kumamoto University. NOD.Cg-Rag1^{tm1Mom} Il2rg^{tm1Wjl}/SzJ (NRG), NOD.Cg-Rag1tm1MomIl2rgtm1WjlTg (CMV-IL3, CSF2, KITLG)1Eav/J (NRGS) and NOD.Cg-Prkdcscid Il2rgtm WjlTg (CMV-IL3, CSF2, KITLG)1Eav/MloySzJ (NSGS) mice were bred and maintained in the pathogen-free facility of CCHMC. The p53$^{-/-}$ mice, in which 5′ part of exon 2 including translation initiation site of *Trp53* gene was replaced with Neomycin resistance gene, were provided from the RIKEN BioResource Center (Ibaragi, Japan; Tsukada *et al*, 1993). All animal experiments were performed in accordance with approved protocols from the Laboratory Animal Research Center of The Institute of Medical Science at the University of Tokyo (approval number: PA15-109, PA18-46) and with an approved animal study IACUC protocol at CCHMC.

Plasmids and viral infection

We used pMSCV-MLL-AF9-pgk-EGFP, pMSCV-MLL-AF9-pgk-puro, pMSCV-neo-MLL-ENL, pMSCV-MLL-Af4-PGK-GFP (gifts from Dr. Akihiko Yokoyama) for the expression of MLL-fusion oncogenes. Lentiviral vector MISSION pLKO.1-shRNA-puro constructs targeting human p21 (CDKN1A; TRCN0000287021 [sh-p21] were obtained from Sigma-Aldrich). We also used HA-tagged K63Ub in a pcDNA3.1 vector (a gift from Dr. Daisuke Oikawa) and FLAG-tagged mTraf6 in a pME18S vector (a gift from Drs. Jun-ichiro Inoue and Mizuki Yamamoto). T7-p53DD-pcDNA3 (Addgene #25989) was obtained from Addgene, and we cloned it to pMYs-IRES-NGFR vector (Tamura *et al*, 2019). Retroviruses were produced by transient transfection of Plat-E packaging cells (Morita *et al*, 2000) with retroviral constructs using the calcium-phosphate method. Lentiviruses were produced by transient transfection of 293 T cells with viral plasmids along with gag-, pol-, and env-expressing plasmids (pMD2.G and psPAX) using the calcium-phosphate method (Goyama *et al*, 2016). pMD2.G (Addgene plasmid #12259; http://n2t.net/addgene:12259; RRID: Addgene_12259) and psPAX2 (Addgene plasmid #12260; http://n2t.net/addgene:12260; RRID: Addgene_12260) were gifts from Didier Trono. Retrovirus transduction to the leukemia cells was performed using Retronectin (Takara Bio Inc, Otsu, Shiga, Japan).

Cell culture

Human CB CD34$^+$ cells were isolated using an EasySep CD34 selection kit (STEMCELL Technologies) or a CD34 MicroBead Kit (Miltenyi Biotec). The CB CD34$^+$ cells, CB cells transduced with MLL-AF9, MLL-ENL, or RUNX1-ETO, and primary human AML cells were cultured in Iscove's modified Dulbecco's media (IMDM) containing 20% BIT9500 (STEMCELL Technologies) or StemSpan SFEM II medium (#ST-09655, STEMCELL Technologies) containing 1% penicillin–streptomycin together with 10 ng/ml rhSCF (#255-SC, R&D Systems), 10 ng/ml rhTPO (#288-TP, R&D Systems), 10 ng/ml rmFlt-3 ligand (#427-FL, R&D Systems), 10 ng/ml rhIL-3 (#203-IL, R&D Systems) and 10 ng/ml rhIL-6 (#206-IL, R&D Systems; Goyama *et al*, 2016). CB cells transduced with MLL-Af4 and primary human ALL cells were cultured with murine stromal cell line MS-5 in MEMα medium (#12561–056, gibco) containing 20% FBS, 1% penicillin–streptomycin and 10 ng/ml human rhSCF, 10 ng/ml rmFlt-3 ligand, 10 ng/ml rhIL-7(#200–07, PeproTech). Human leukemia cell lines (MOLM13, NOMO1, THP1, MV4;11, HL60, Kasumi-1, OCI-AML3 and U937) were cultured in Roswell Park Memorial Institute (RMPI)-1640 medium (#189–02025, FUJIFILM Wako) with 10% fetal bovine serum (FBS; #FB-1365/500, Biosera), 1% penicillin–streptomycin. 293 T cells were cultured in Dulbecco's Modified Eagle Medium (DMEM) medium (#044–29765, Wako) with 10% FBS and 1% penicillin–streptomycin. The detailed information of primary patient samples was provided in Appendix Table S1.

In vitro drug sensitivity assay

To test the sensitivity of AML cells to IMPDH inhibitors, cells were cultured in a 96-well flat-bottom plate at a density of 2–5 × 10^4/well in 100 μl of culture medium. Then, the cells were cultured with titrating doses of MPA (MP Biomedicals, Cat#194172; CAS:24280–93-1) or FF-10501-01 (FUJIFILM Corporation) together with or without 100 μM Guanosine (#G6264-6G, Sigma-Aldrich or ACROS ORGANICS, Cat#411130250; CAS:118–00-3). After 72 h, we added 10 μl of the WST-8 (#07553–44, Nacalai Tesque) or WST-1 (#11644807001, Roche) to each well and incubated the cells for 3–4 h. The absorbance of each sample was measured at a wavelength of 450 nm by a microplate reader. The combined effect of MPA + Decitabine and MPA + Venetoclax was evaluated with the similar *in vitro* assays. Combination index (CI) was calculated from the CI equation algorithms (Chou, 2006) using CompuSyn software.

Mouse transplantation assay and *in vivo* drug treatment

Mouse bone marrow c-Kit+ cells derived from wild-type or Trp53 (−/−) mice were transduced with MLL-AF9 (co-expressinf GFP) and were transplanted intravenously into sublethally irradiated (525 cGy) recipient mice. The MLL-AF9-expressing leukemia cells were then harvested from spleens of moribund mice and were serially transplanted into recipient mice. The serial transplantation was subsequently repeated several times to generate MLL-AF9 cells with strong leukemogenicity. These wild-type and Trp53(−/−) MLL-AF9 cells were injected intravenously (1 × 10^6 cells/mouse) into nonirradiated recipient mice. For drug studies, MMF (CAS RN: 128794–94-5, Product Number: M2387 from Tokyo chemical industry CO., Ltd), FF-10501-01 and DS-5272 (Daiichi Sankyo) were dissolved in 0.5 w/v% Methyl Cellulose 400 Solution (#133-17815, Wako, Japan). Pam3CSK4 (Catalog Code: tlrl-pms, InvivoGen) was dissolved in PBS. The MLL-AF9-bearing mice were orally administered with vehicle or the IMPDH inhibitors (120 mg/kg MMF and 160 mg/kg FF-10501-01) on an every 2-day schedule from Day 1 or Day 12 (FF-10501-01 and Pam3CSK4 combined treatments were

started from Day 3) after the transplantation. In some experiments, the MLL-AF9-bearing mice were also intraperitoneally injected with 8 μg Pam3CSK4, or orally administered with 80 mg/kg of DS-5272. For the RNA-seq, flow cytometry analysis, single-cell mass cytometry, and metabolome analysis, the MLL-AF9-bearing mice were treated with 120 mg/kg MMF, 160 mg/kg FF-10501-01, or vehicle in two consecutive days. Bone marrow cells were then harvested from the mice 48 h after the first administration of the drugs.

Vcam1 depletion and overexpression using CRISPR/Cas9 and SunTag systems

To generate sgRNA expression vectors targeting Vcam1 (Vcam1-knockout-1 and 2) or nontargeting (NT) control, annealed oligonucleotides were cloned into the lentiGuide-Puro vector (Addgene #52963; Sanjana *et al*, 2014) or pLKO5.sgRNA.EFS.tRFP657 vector (Addgene #57824; Heck *et al*, 2014). MLL-AF9 cells were first infected with lentiCas9-Blast (Addgene #52962; Sanjana *et al*, 2014) and were then selected for stable expression of Cas9 in Methocult™ medium (#M3234, STEMCELL Technologies) with 20 ng/ml rmSCF (455-MC, R&D Systems), 10 ng/ml rmIL-3 (403-ML, R&D Systems), 10 ng/ml rmIL-6 (406-ML, R&D Systems), 10 ng/ml rmGM-CSF (415-ML, R&D Systems) together with blasticidin (10 μg/ml). The MLL-AF9/Cas9 cells were infected with the lentivirus carrying guide RNA sequence for 24 h and selected with puromycin (1 μg/ml) in the same cytokine-containing Methocult™ medium or directly transplanted into recipient mice.

Vcam1 overexpression was achieved using SunTag system (Tanenbaum *et al*, 2014). Plasmids encoding the Suntag components are pHRdSV40-dCas9-10 × GCN4_v4-P2A-BFP (Addgene #60903) and pHRdSV40-scFv-GCN4-sfGFP-VP64-GB1-NLS (Addgene #60904). We cloned them into pMYs-IRES-blasticidin and pMYs-IRES-tdTomato retroviral vector, respectively. MLL-AF9 cells were infected with the retrovirus carrying the SunTag components for 48 h on Retronectin (Takara Bio Inc, Otsu, Shiga, Japan). The SunTag-transduced MLL-AF9 cells were selected in the cytokine-containing Methocult™ medium (see above) with 10 μg/ml blasticidin, and the tdTomato-positive cells were sorted by FACS Aria (Becton Dickinson, SORPAria). The MLL-AF9/SunTag cells were then infected with the lentivirus carrying guide RNA sequence targeting Vcam1 promoter regions (Vcam1-SunTag-1 and 2) for 24 h and selected with puromycin (1 μg/ml).

Sequences for the sgRNAs are provided as follows: NT: 5′-cgc ttccgcggcccgttcaa-3′, Vcam1-knockout-1: 5′-caccggctggaacgaagtatcca cg-3′, Vcam1-knockout-2: 5′-caccggcccactaaacgcgaaggtg-3′, Vcam1-SunTag-1: 5′-gcagctgaagggggttaacgt-3′, Vcam1-SunTag-2: 5′-cagctgaag gggttaacgtg-3′. IMPDH2-knockout-1: 5′-gagaaaatcaatgtccctgg-3′, IMPDH2-knockout-2: 5′-caccgattcaggtgtacagttgtgg-3′. IMPDH1-knockout-1: 5′-gagtt ggagccacctgaacg-3′, IMPDH1-knockout-2: 5′-tcatcgcaatcattgacgat-3′.

Western blotting and immunofluorescence

Human cord blood (CB) cells, MLL-AF9- or RUNX1-ETO-expressing CB cells, and MOLM13 cells were lysed in SDS sample buffer (125 mM Tris–HCl pH 6.8, 4% sodium dodecyl sulfate (SDS), 20% glycerol, 0.01% bromophenol blue, 10% 2-mercaptoethanol). Whole-cell lysates were subjected to SDS-polyacrylamide gel electrophoresis (SDS/PAGE) and transferred to a polyvinylidene fluoride membrane (Bio-Rad). Mouse MLL-AF9 cells were lysed and subjected to SDS/PAGE as described above. The blot was incubated with antibodies described in Appendix Table S2. Signals were detected with SuperSignalWest Pico Chemiluminescent Substrate (Pierce, Rockford, IL, USA), or with ECL Western Blotting Substrate (Promega, Madison, WI, USA) and visualized with image quant LAS 4000 (Fujifilm Life Science, Roche Diagnostics) or Amersham Imager 600 (GE Healthcare).

Transfection and immunoprecipitation

Two hundred and ninety three T cells were transiently transfected with 3 μg of vector, FLAG-tagged TRAF6, and HA-tagged ubiquitin mixed with 30 μl polyethyleneimine (PEI). The cells were cultured for 48 h after the transfection and lysed in Cell Lysis Buffer (Cell Signaling Technology, Danvers, MA; #9803). For immunoprecipitation, the cell lysates were incubated with an anti-FLAG (SIGMA, #F3165) antibody for 30 min at 4°C. Then, the samples were incubated with Dynabeads protein-G (Themo Fisher Scientific, USA) for 30 min at 4°C. The precipitates were washed three times with the cell lysis buffer containing 1 mM phenylmethanesulfonyl fluoride, subjected to SDS/PAGE, and were analyzed by Western blotting with anti-HA (Roche Applied Sciences, 3F10, #11867423001) antibody.

Quantitative PCR

Mouse BMDMs were treated with MPA (10 μM), guanosine (100 μM), and Pam3CSK4 (100 ng/ml) for 4 h, and total RNA was extracted from them using the RNeasy Mini kit (QIAGEN), and reverse-transcribed using the High-Capacity cDNA Reverse Transcription Kit (Applied Biosystems, Foster City, CA, USA) with the deoxyribonuclease I (Invitrogen - Thermo Fisher Scientific - MA, USA). Quantitative PCR (qPCR) was performed using SYBR Premix EX Taq (Takara Bio) and Rotor-Gene Q (Qiagen, Venlo, The Netherlands). The delta–delta Ct method was used to calculate the relative gene expression values in qPCR. Sequences of the primers used for quantitative PCR in this study, from 5′ to 3′ are as follows: Tnf-α Forward 5′- TCTTCTCATTCCTGCTTGTGG-3′; Tnf-α Reverse 5′- GG TCTGGGCCATAGAACTGA-3′; IL-1β Forward 5′- CTGGTGTGTGA CGTTCCCATTA-3′; IL-1β Reverse 5′- CCGACAGCACGAGGCTTT-3′; Gapdh Forward 5′- TTGATGGCAACAATCTCCAC-3′; Gapdh Reverse 5′ CGTCCCGTAGACAAAATGGT-3′.

NF-κB reporter assay using toll-like receptor expressing Ba/F3 cells

Ba/F3 cells were first transduced with a reporter construct NF-κB-GFP and were then transduced with TLR4, TLR5, or TLR7 (Matsumoto *et al*, 2008; Shibata *et al*, 2012; Sato *et al*, 2017). These BaκB cells were cultured in RPMI medium containing 10% FBS, 1% penicillin–streptomycin and 1 ng/ml murine IL-3 (#403-ML, R&D Systems). To evaluate the effect of MPA and TLR ligands, titrating doses of MPA (1–10 μM) were added together with TLR ligands (TLR1/2 ligand: 100 ng/ml of Pam3CSK4 [#ab142085, Abcam]), TLR4 ligand: 100 ng/ml Lipid A (E. coli; #24005-s, PEPTIDE INSTITUTE); TLR5 ligand: 10 ng/ml Flagellin (#FLA-ST, InvivoGen); TLR7/8 ligand: 100 ng/ml of R848 (#tlrl-r848, InvivoGen) to the

©2022 The Authors

culture. After 24 h of incubation, GFP expression in BaκB cells was measured by a BD FACSVerse analyzer.

Morphological analysis

Cytospin preparations were stained with Wright-Giemsa. Images were obtained with a BX51 microscope and a DP12 camera (Olympus).

Flow cytometry analysis

Mouse bone marrow cells were isolated from femurs and tibias using bone-crushing technique. After filtered into Falcon Cell Strainer (mesh size 40 μm, Cat# 087711), red cells were removed using 1x red blood cell (RBC) lysis Buffer. Cells were stained by antibodies (Appendix Table S3) in PBS containing 2% FBS (staining medium) for 30 min on ice. Cells were then washed twice in a staining medium and analyzed by FACS Verse or sorted by FACS Aria (BD Biosciences, San Jose, CA, USA). Cell cycle (#V35003, Vybrant DyeCycle Violet stain; Invitrogen) and apoptosis (Annexin V-APC kit; BD Biosciences) analyses were performed according to the manufacturer's recommendations.

Single-cell mass cytometry analysis

A summary of all mass cytometry antibodies, reporter isotopes, and concentrations used for analysis are provided in Appendix Table S4. Primary conjugates of mass cytometry antibodies were purchased preconjugated from Fluidigm or prepared using the MaxPAR antibody conjugation kit (Fluidigm PRD002 Version7) according to the manufacturer's recommended protocol. For sample preparation, MLL-AF9 cells were isolated from femurs and tibias of mice treated with vehicle (0.5 w/v% Methyl Cellulose 400 Solution) or FF-10501-01 24 h before. After filtration with Falcon Cell Strainer (mesh size 40 μm, Cat# 087711), red cells were removed using 1x Ammonium-Chloride-Potassium (ACK; Thermo Fisher, #A1049201) buffer. These cells were counted and resuspended in PBS (1×10^7/ml), stained with live/dead cell indicator Cell-IDTM Cisplatin (Fluidigm, Cat# 201198) at a final concentration of 5 μM, and were then fixed with 1 × Maxpar FixI Buffer (Fluidigm, Cat# 201065). 1×10^6 cells/sample were aliquoted and were stained with the 26 antibodies (Appendix Table S4) according to the Maxpar Phospho-protein Staining (Fluidigm, PRD016 Version3) protocol. At the end of cell staining, cells were labeled with Cell-IDTM Intercalator–Ir in Maxpar Cell Staining Buffer (Fluidigm Cat#201068) at 4°C overnight for DNA intercalation. Stained samples were washed twice with Maxpar Cell Staining Buffer (Fluidigm Cat#201068) and once with Maxpar Water (Fluidigm, Cat# 201069). Then, cells (2.5×10^5 cells/ml) in Maxpar Water (Fluidigm, Cat# 201069) were added with EQTM Four Element Calibration Beads (Fluidigm, Cat#201087) diluted to 1/10. The samples were filtered with 40 μm Cell Strainer Snap Cap Falcon™ Test Tube (Cat#0877123) immediately prior to sample acquisition on HeliosTM mass cytometer (Fluidigm, Cat#107002). Approximately 100 K events per sample were acquired for each sample. FCS files were normalized to the EQ Four Element Calibration Beads using Helios software (Version 6.5.358) and were analyzed by SPADE. Gating and extraction of median expression levels were performed using Cytobank (https://premium.cytobank.org) under a condition of Target Number of Nodes is 150. The file was downsampled to an absolute number of 5,000. The $CD45.2^+$ population and eight clustering channels (141Pr_Gr1, 143rd_CD41, 145_Nd_CD4, 160Gd_B220, 168Er_CD8a, 170Er_CD49d, 172Yb_Mac-1, 173Yb_ckit) were selected to create categorization for the SPADE.

Metabolome analysis

Mouse MLL-AF9 cells (1×10^6 cells/mouse) were intravenously injected into C57BL/6J recipient mice (total $n = 6$). MLL-AF9 mice were treated with vehicle or FF-10501-01 (160 mg/kg) 10 days after transplantation. Bone marrow cells were harvested from the mice 24 h after the treatment, and 5×10^5 GFP^+ cells were sorted for metabolome analysis. After metabolite extraction from sorted cells, metabolome analysis was performed as described previously (Kunisawa *et al*, 2015). Briefly, frozen sorted cell fractions together with internal standard (IS) compound 2-morpholinoethanesulfonic acid was suspended in ice-cold methanol (500 μl) followed by the addition of an equal volume of chloroform and 0.4 times the volume of ultrapure water (LC/MS grade, Wako). The suspension was then centrifuged at 15,000 *g* for 15 min at 4°C. After centrifugation, the aqueous phase was ultra-filtered using an ultrafiltration tube (Ultrafree MC-PLHCC, Human Metabolome Technologies). The filtrate was concentrated with a vacuum concentrator (SpeedVac, Thermo). The concentrated filtrate was dissolved in 25 μl of ultrapure water and used for ion chromatography (IC)-MS analyses as described below. For metabolome analysis of anion metabolites were measured using an orbitrap-type MS (Q-Exactive focus, Thermo Fisher Scientific, San Jose, CA), connected to a high-performance IC system (ICS-5000+, Thermo Fisher Scientific) that enables us to perform highly selective and sensitive metabolite quantification owing to the IC-separation and Fourier Transfer MS principle. The IC was equipped with an anion electrolytic suppressor (Thermo Scientific Dionex AERS 500) to convert the potassium hydroxide gradient into pure water before the sample enters the mass spectrometer. The separation was performed using a Thermo Scientific Dionex IonPac AS11-HC, 4-μm particle size column. IC flow rate was 0.25 ml/min supplemented post-column with 0.18 ml/min makeup flow of MeOH. The potassium hydroxide gradient conditions for IC separation are as follows: from 1 mM to 100 mM (0–40 min), 100 mM (40–50 min), and 1 mM (50.1–60 min), at a column temperature of 30°C. The Q Exactive focus mass spectrometer was operated under an ESI negative mode for all detections. Full mass scan (m/z 70–900) was used at a resolution of 70,000. The automatic gain control target was set at 3×10^6 ions, and maximum ion injection time (IT) was 100 ms. Source ionization parameters were optimized with the spray voltage at 3 kV and other parameters were as follows: transfer temperature at 320°C, S-Lens level at 50, heater temperature at 300°C, Sheath gas at 36, and Aux gas at 10.

RNA-Seq analysis

Mouse MLL-AF9 cells (1×10^6 cells/mouse) were intravenously injected into C57BL/6J recipient mice (total $n = 6$). MLL-AF9 mice were treated with vehicle or FF-10501-01 (160 mg/kg) 10 days after transplantation. Bone marrow cells were harvested from the mice 24 h after the treatment, and 5×10^5 GFP^+ cells were sorted for RNA-Seq. Total RNA was extracted using RNeasy Mini Kit (Qiagen),

The paper explained

Problem

Acute myeloid leukemia (AML) is an aggressive form of blood cancer that can develop rapidly without treatment. The *MLL* gene on chromosome 11q23 is frequently disrupted in certain subtypes of AML, resulting in the fusion of *MLL* to a number of different partner genes. The MLL-fusions have strong leukemogenic potential to drive AML development. Despite recent advances in therapeutic approaches, patients with MLL-fusion AML still have poor outcomes under the current standard of care. Therefore, high demand exists to develop new therapies for MLL-fusion AML.

Results

Inosine monophosphate dehydrogenase (IMPDH) is an enzyme that play an essential role in guanine nucleotide synthesis pathway. IMPDH inhibitors have been prescribed to prevent rejection after organ transplantation. In this study, we found the potent antileukemia effect of two IMPDH inhibitors, MPA and FF-10501-01, on MLL-fusion AMLs. The alternate-day administration of IMPDH inhibitors to mice effectively suppressed the development of AML *in vivo* without showing devastating effects on immune cells. As a mechanism, we found that IMPDH inhibitors induce overactivation of the TLR signaling and upregulation of VCAM1 in AML cells.

Impact

These data provide a rational basis for clinical testing of IMPDH inhibitors against MLL-fusion AMLs. Given the established safety as immunosuppressants, repurposing MPDH inhibitors could provide an affordable, safe, and effective therapy for leukemia patients. Our study also revealed the previously unrecognized crosstalk between intracellular levels of guanine nucleotides and TLR signaling, which will merit future studies.

and the quality and quantity of RNA were checked using Agilent High Sensitivity RNA Screen Tape and Qubit. RNA libraries were prepared using 500 ng total RNA with SureSelect Strand-Specific RNA Preparation Kit (Agilent) according to the manufacturer's protocol. The quality and quantity of these libraries were checked using Agilent TapeStation D1000 and KAPA Library Quantification Kits [KAPA BioSystems] / Real-time PCR Systems Step One Plus [Applied Biosystems]. These libraries were sequenced on the Illumina HiSeq2500 System with 2 × 100 nucleotide paired end reads according to the manufacturer's protocol. Derived reads were processed using cutadapt (1.8.1) and fastx-toolkit (0.0.13) to remove Illumina adaptor sequence and to trim low-quality bases. Quality of reads were assessed using FastQC. Processed reads were aligned to GRCm38 reference transcripts using TopHat(2.1.1)-Cufflinks(2.2.1) pipeline (Trapnell *et al*, 2010, 2014; Kim *et al*, 2013) to derive gene FPKM values. For clustering analysis, normalized read counts were further transformed by the variance-stabilizing transformation method in DESeq2. They were then subjected to hierarchical clustering analysis with Ward's method.

Statistical analyses

GraphPad Prism 9 was used for statistical analyses. Unpaired Student's *t*-test (two-tailed) and Ordinary one-way ANOVA were used for pairwise comparisons of significance. The log-rank (Mantel-Cox) was used for the survival curves comparison. The differentially expressed gene of RNA-seq was analyzed by Cuffdiff. A *P*-value > 0.05 was considered as not significant (ns). Animal experiments were neither blinded nor randomized. The type of replication (biological or technical) is indicated in figure legends. Sample size was decided based on our previous experience in the field, not predetermined by a statistical method. All data are shown as mean ± SEM.

Data availability

All data needed to evaluate the conclusions are present in the paper and/or the Appendix files.

The datasets produced in this study are available in the following databases:

- Mass Cytometry data: FCM files (DOI: 10.17632/984b9rh5h4.1; https://data.mendeley.com/datasets/984b9rh5h4/1)
- RNA-seq data: GSE214474 (https://www.ncbi.nlm.nih.gov/geo/query/acc.cgi?acc=GSE214474)

Expanded View for this article is available online.

Acknowledgments

This work was supported by a Grant-in-Aid for Challenging Research (Exploratory; 19K22554, SG), a Grant-in-Aid for Scientific Research (B; 19H03685 and 22H03100, SG), a grant from Kobayashi Foundation for Cancer Research (SG), a grant from Pharmacological Research Group (SG), a grant from Princess Takamatsu Cancer Research Fund (SG), NIH grants (R01NS089815 and R01CA255331, A.T.S.) and St. Baldrick's Foundation Scholar Award (B.M.). During the staying at CCHMC, X.L is financially supported by the Global Leader Program for Social Design and Management (GSDM) and CBDI (Cancer and Blood Diseases Institute) of CCHMC. This work was also partially supported by FUJIFILM Corporation. We sincerely thank Nicole Manning and Christina Sexton at CCHMC for their expert technical assistance. We thank Dr. Koichi Matsuda for providing p53 knock-out mice. We thank Dr. Xiaomin Feng for the technical assistance to use the single-cell mass cytometry system at IRCMS. We also thank Drs. Akihiko Yokoyama, Daisuke Oikawa, Jun-ichiro Inoue, and Mizuki Yamamoto for providing plasmids. We sincerely thank the Flow Cytometry Core, the Mouse Core at IMSUT, CCHMC and IRCMS for their help.

Author contributions

Xiaoxiao Liu: Conceptualization; resources; data curation; software; formal analysis; validation; investigation; visualization; methodology; writing – original draft; project administration; writing—review and editing. **Naru Sato:** Resources; data curation; supervision; methodology; writing—review and editing. **Tomohiro Yabushita:** Conceptualization; resources; software; supervision; methodology; writing—review and editing. **Jingmei Li:** Data curation; validation; writing—review and editing. **Yuhan Jia:** Data curation; validation; writing—review and editing. **Moe Tamura:** Data curation; methodology; writing—review and editing. **Shuhei Asada:** Supervision; writing—review and editing. **Takeshi Fujino:** Resources; supervision; methodology; writing—review and editing. **Tsuyoshi Fukushima:** Conceptualization; supervision; writing—review and editing. **Taishi Yonezawa:** Validation; methodology; writing—review and editing. **Yosuke Tanaka:** Supervision; funding acquisition; writing—review and editing. **Tomofusa Fukuyama:** Supervision; funding acquisition; writing—review and

©2022 The Authors

editing. **Akiho Tsuchiya:** Data curation; validation; methodology; writing—review and editing. **Shiori Shikata:** Data curation; methodology; writing—review and editing. **Hiroyuki Iwamura:** Supervision; validation; writing—review and editing. **Chieko Kinouchi:** Supervision; validation; writing—review and editing. **Kensuke Komatsu:** Supervision; validation; writing—review and editing. **Satoshi Yamasaki:** Resources; software; formal analysis; visualization; methodology; writing—review and editing. **Tatsuhiro Shibata:** Resources; software; supervision; validation; visualization; writing—review and editing. **Atsuo Sasaki:** Conceptualization; supervision; funding acquisition; methodology; writing—review and editing. **Janet Schibler:** Data curation; formal analysis; validation. **Mark Wunderlich:** Resources; data curation; software; formal analysis; supervision; validation; methodology; writing—review and editing. **Eric O'Brien:** Resources; supervision; validation; methodology; writing—review and editing. **Benjamin Mizukawa:** Resources; supervision; funding acquisition; validation; writing—review and editing. **James C Mulloy:** Resources; supervision; funding acquisition. **Yuki Sugiura:** Resources; software; formal analysis; visualization; methodology. **Hitoshi Takizawa:** Resources; software; supervision; methodology; writing—review and editing. **Takuma Shibata:** Conceptualization; resources; supervision; validation; methodology; writing—review and editing. **Kensuke KM Miyake:** Conceptualization; resources; supervision; methodology; writing—review and editing. **Toshio Kitamura:** Supervision; funding acquisition; investigation; project administration; writing—review and editing. **Susumu Goyama:** Conceptualization; resources; data curation; software; formal analysis; supervision; funding acquisition; validation; investigation; visualization; methodology; writing—original draft; project administration; writing—review and editing.

Disclosure and competing interests statement

H.I., C.K. and K.K. belong to FUJIFILM Corporation. S.G. has research support from FUJIFILM Corporation. Other authors declare that they have no competing interests.

References

Asada S, Goyama S, Inoue D, Shikata S, Takeda R, Fukushima T, Yonezawa T, Fujino T, Hayashi Y, Kawabata KC *et al* (2018) Mutant ASXL1 cooperates with BAP1 to promote myeloid leukaemogenesis. *Nat Commun* 9: 2733

Bajaj J, Konuma T, Lytle NK, Kwon HY, Ablack JN, Cantor JM, Rizzieri D, Chuah C, Oehler VG, Broome EH *et al* (2016) CD98-mediated adhesive signaling enables the establishment and propagation of acute myelogenous leukemia. *Cancer Cell* 30: 792 – 805

Barve A, Casson L, Krem M, Wunderlich M, Mulloy JC, Beverly LJ (2018) Comparative utility of NRG and NRGS mice for the study of normal hematopoiesis, leukemogenesis, and therapeutic response. *Exp Hematol* 67: 18 – 31

Chou TC (2006) Theoretical basis, experimental design, and computerized simulation of synergism and antagonism in drug combination studies. *Pharmacol Rev* 58: 621 – 681

Collart FR, Chubb CB, Mirkin BL, Huberman E (1992) Increased INOSINE-5'-phosphate dehydrogenase gene-expression IN solid tumor-tissues and tumor-cell lines. *Cancer Res* 52: 5826 – 5828

Emmanuel N, Ragunathan S, Shan Q, Wang F, Giannakou A, Huser N, Jin GX, Myers J, Abraham RT, Unsal-Kacmaz K (2017) Purine nucleotide availability regulates mTORC1 activity through the Rheb GTPase. *Cell Rep* 19: 2665 – 2680

Eriksson M, Pena-Martinez P, Ramakrishnan R, Chapellier M, Hogberg C, Glowacki G, Orsmark-Pietras C, Velasco-Hernandez T, Lazarevic VL, Juliusson G *et al* (2017) Agonistic targeting of TLR1/TLR2 induces p38 MAPK-dependent apoptosis and NF kappa B-dependent differentiation of AML cells. *Blood Adv* 1: 2046 – 2057

Feral CC, Nishiya N, Fenczik CA, Stuhlmann H, Slepak M, Ginsberg MH (2005) CD98hc (SLC3A2) mediates integrin signaling. *Proc Natl Acad Sci USA* 102: 355 – 360

Garcia-Manero G, Pemmaraju N, Alvarado Y, Naqvi K, Ravandi F, Jabbour E, De Lumpa R, Kantarjian H, Advani A, Mukherjee S *et al* (2020) Results of a phase 1/2a dose-escalation study of FF-10501-01, an IMPDH inhibitor, in patients with acute myeloid leukemia or myelodysplastic syndromes. *Leuk Lymphoma* 61: 1943 – 1953

Goyama S, Mulloy JC (2013) NF-kappa B: a coordinator for epigenetic regulation by M LL. *Cancer Cell* 24: 401 – 402

Goyama S, Schibler J, Cunningham L, Zhang Y, Rao Y, Nishimoto N, Nakagawa M, Olsson A, Wunderlich M, Link KA *et al* (2013) Transcription factor RUNX1 promotes survival of acute myeloid leukemia cells. *J Clin Invest* 123: 3876 – 3888

Goyama S, Schibler J, Gasilina A, Shrestha M, Lin S, Link KA, Chen J, Whitman SP, Bloomfield CD, Nicolet D *et al* (2016) UBASH3B/Sts-1-CBL axis regulates myeloid proliferation in human preleukemia induced by AML1-ETO. *Leukemia* 30: 728 – 739

Hafliger P, Charles RP (2019) The L-type amino acid transporter LAT1An emerging target in cancer. *Int J Mol Sci* 20: 2428

Harris WJ, Huang X, Lynch JT, Spencer GJ, Hitchin JR, Li YY, Ciceri F, Blaser JG, Greystoke BF, Jordan AM *et al* (2012) The histone demethylase KDM1A sustains the oncogenic potential of MLL-AF9 leukemia stem cells. *Cancer Cell* 21: 473 – 487

Hayashi Y, Goyama S, Liu X, Tamura M, Asada S, Tanaka Y, Fukuyama T, Wunderlich M, O'Brien E, Mizukawa B *et al* (2019) Antitumor immunity augments the therapeutic effects of p53 activation on acute myeloid leukemia. *Nat Commun* 10: 4869

Heck D, Kowalczyk MS, Yudovich D, Belizaire R, Puram RV, McConkey ME, Thielke A, Aster JC, Regev A, Ebert BL (2014) Generation of mouse models of myeloid malignancy with combinatorial genetic lesions using CRISPR-Cas9 genome editing. *Nat Biotechnol* 32: 941 – 946

Hedstrom L (2009) IMP dehydrogenase: structure, mechanism, and inhibition. *Chem Rev* 109: 2903 – 2928

Huang F, Ni M, Chalishazar MD, Huffman KE, Kim J, Cai L, Shi XL, Cai F, Zacharias LG, Ireland AS *et al* (2018) Inosine monophosphate dehydrogenase dependence in a subset of small cell lung cancers. *Cell Metab* 28: 369 – 382.e5

Ignatz-Hoover JJ, Wang H, Moreton SA, Chakrabarti A, Agarwal MK, Sun K, Gupta K, Wald DN (2015) The role of TLR8 signaling in acute myeloid leukemia differentiation. *Leukemia* 29: 918 – 926

Kim D, Pertea G, Trapnell C, Pimentel H, Kelley R, Salzberg SL (2013) TopHat2: accurate alignment of transcriptomes in the presence of insertions, deletions and gene fusions. *Genome Biol* 14: R36

Kofuji S, Hirayama A, Eberhardt AO, Kawaguchi R, Sugiura Y, Sampetrean O, Ikeda Y, Warren M, Sakamoto N, Kitahara S *et al* (2019) IMP dehydrogenase-2 drives aberrant nucleolar activity and promotes tumorigenesis in glioblastoma. *Nat Cell Biol* 21: 1003 – 1014

Kong DH, Kim YK, Kim MR, Jang JH, Lee S (2018) Emerging roles of vascular cell adhesion Molecule-1 (VCAM-1) in immunological disorders and cancer. *Int J Mol Sci* 19: 1057

Kunisawa J, Sugiura Y, Wake T, Nagatake T, Suzuki H, Nagasawa R, Shikata S, Honda K, Hashimoto E, Suzuki Y *et al* (2015) Mode of bioenergetic

©2022 The Authors

metabolism during B cell differentiation in the intestine determines the distinct requirement for vitamin B-1. *Cell Rep* 13: 122–131

Kuo HP, Wang Z, Lee DF, Iwasaki M, Duque-Afonso J, Wong SHK, Lin CH, Figueroa ME, Su J, Lemischka IR *et al* (2013) Epigenetic roles of MLL Oncoproteins are dependent on NF-kappa B. *Cancer Cell* 24: 423–437

Lee J, Wu CCN, Lee KJ, Chuang TH, Katakura K, Liu YT, Chan M, Tawatao R, Chung M, Shen C *et al* (2006) Activation of anti-hepatitis C virus responses via toll-like receptor 7. *Proc Natl Acad Sci USA* 103: 1828–1833

Liang KW, Volk AG, Haug JS, Marshall SA, Woodfin AR, Bartom ET, Gilmore JM, Florens L, Washburn MP, Sullivan KD *et al* (2017) Therapeutic targeting of MLL degradation pathways in MLL-rearranged leukemia. *Cell* 168: 59–72.e13

Lin S, Luo RT, Ptasinska A, Kerry J, Assi SA, Wunderlich M, Imamura T, Kaberlein JJ, Rayes A, Althoff MJ *et al* (2016) Instructive role of MLL-fusion proteins revealed by a model of t(4;11) pro-B acute lymphoblastic leukemia. *Cancer Cell* 30: 737–749

Matsumoto F, Saitoh SI, Fukui R, Kobayashi T, Tammura N, Konnio K, Kusumoto Y, Akashi-Takamura S, Miyake K (2008) Cathepsins are required for toll-like receptor 9 responses. *Biochem Biophys Res Commun* 367: 693–699

Messina E, Gazzaniga P, Micheli V, Guaglianone MR, Barbato S, Morrone S, Frati L, Agliano AM, Giacomello A (2004) Guanine nucleotide depletion triggers cell cycle arrest and apoptosis in human neuroblastoma cell lines. *Int J Cancer* 108: 812–817

Mootha VK, Lindgren CM, Eriksson KF, Subramanian A, Sihag S, Lehar J, Puigserver P, Carlsson E, Ridderstrale M, Laurila E *et al* (2003) PGC-1 alpha-responsive genes involved in oxidative phosphorylation are coordinately downregulated in human diabetes. *Nat Genet* 34: 267–273

Morita S, Kojima T, Kitamura T (2000) Plat-E: An efficient and stable system for transient packaging of retroviruses. *Gene Ther* 7: 1063–1066

Murase M, Iwamura H, Komatsu K, Saito M, Maekawa T, Nakamura T, Yokokawa T, Shimada Y (2016) Lack of cross-resistance to FF-10501, an inhibitor of inosine 5'- monophosphate dehydrogenase, in azacitidine-resistant cell lines selected from SKM-1 and MOLM-13 leukemia cell lines. *Pharmacol Res Perspect* 4: e00206

Naffouje R, Grover P, Yu HY, Sendilnathan A, Wolfe K, Majd N, Smith EP, Takeuchi K, Senda T, Kofuji S *et al* (2019) Anti-tumor potential of IMP dehydrogenase inhibitors: A century-long story. *Cancer* 11: 1346

Neff T, Sinha AU, Kluk MJ, Zhu N, Khattab MH, Stein L, Xie HF, Orkin SH, Armstrong SA (2012) Polycomb repressive complex 2 is required for MLL-AF9 leukemia. *Proc Natl Acad Sci USA* 109: 5028–5033

Okada Y, Feng Q, Lin YH, Jiang Q, Li YQ, Coffield VM, Su LS, Xu GL, Zhang Y (2005) hDOT1L links histone methylation to leukemogenesis. *Cell* 121: 167–178

Pinho S, Wei QZ, Maryanovich M, Zhang DC, Balandran JC, Pierce H, Nakahara F, Di Staulo A, Bartholdy BA, Xus JN *et al* (2022) VCAM1 confers innate immune tolerance on haematopoietic and leukaemic stem cells. *Nat Cell Biol* 24: 290–298

Sanjana NE, Shalem O, Zhang F (2014) Improved vectors and genome-wide libraries for CRISPR screening. *Nat Methods* 11: 783–784

Sato R, Shibata T, Tanaka Y, Kato C, Yamaguchi K, Furukawa Y, Shimizu E, Yamaguchi R, Imoto S, Miyano S *et al* (2017) Requirement of glycosylation machinery in TLR responses revealed by CRISPR/Cas9 screening. *Int Immunol* 29: 347–355

Shibata T, Takemura N, Motoi Y, Goto Y, Karuppuchamy T, Izawa K, Li XB, Akashi-Takamura S, Tanimura N, Kunisawa J *et al* (2012) PRAT4A-dependent expression of cell surface TLR5 on neutrophils, classical monocytes and dendritic cells. *Int Immunol* 24: 613–623

Slany RK (2016) The molecular mechanics of mixed lineage leukemia. *Oncogene* 35: 5215–5223

Subramanian A, Tamayo P, Mootha VK, Mukherjee S, Ebert BL, Gillette MA, Paulovich A, Pomeroy SL, Golub TR, Lander ES *et al* (2005) Gene set enrichment analysis: a knowledge-based approach for interpreting genome-wide expression profiles. *Proc Natl Acad Sci USA* 102: 15545–15550

Sun XX, Dai MS, Lu H (2008) Mycophenolic acid activation of p53 requires ribosomal proteins l5 and l11. *J Biol Chem* 283: 12387–12392

Takeda K, Akira S (2005) Toll-like receptors in innate immunity. *Int Immunol* 17: 1–14

Tamura M, Yonezawa T, Liu XX, Asada S, Hayashi Y, Fukuyama T, Tanaka Y, Kitamura T, Goyama S (2019) Opposing effects of acute versus chronic inhibition of p53 on decitabine's efficacy in myeloid neoplasms. *Sci Rep* 9: 8171

Tanenbaum ME, Gilbert LA, Qi LS, Weissman JS, Vale RD (2014) A protein-tagging system for signal amplification in gene expression and fluorescence imaging. *Cell* 159: 635–646

Tien HF, Hsiao CH, Tang JL, Tsay W, Hu CH, Kuo YY, Wang CH, Chen YC, Chen MC, Lin DT *et al* (2000) Characterization of acute myeloid leukemia with MLL rearrangements - no increase in the incidence of coexpression of lymphoid-associated antigens on leukemic blasts. *Leukemia* 14: 1025–1030

Toubiana J, Rossi AL, Grimaldi D, Belaidouni N, Chafey P, Clary G, Courtine E, Pene F, Mira JP, Claessens YE *et al* (2011) IMPDHII protein inhibits toll-like receptor 2-mediated activation of NF-kappa B. *J Biol Chem* 286: 23319–23333

Trapnell C, Williams BA, Pertea G, Mortazavi A, Kwan G, van Baren MJ, Salzberg SL, Wold BJ, Pachter L (2010) Transcript assembly and quantification by RNA-Seq reveals unannotated transcripts and isoform switching during cell differentiation. *Nat Biotechnol* 28: 511–515

Trapnell C, Roberts A, Goff L, Pertea G, Kim D, Kelley DR, Pimentel H, Salzberg SL, Rinn JL, Pachter L (2014) Differential gene and transcript expression analysis of RNA-seq experiments with TopHat and cufflinks (Nature Protocols (2012) vol 7, pg 562, 2012). *Nat Protoc* 9: 2513

Tricot GJ, Jayaram HN, Lapis E, Natsumeda Y, Nichols CR, Kneebone P, Heerema N, Weber G, Hoffman R (1989) Biochemically directed therapy of leukemia with TIAZOFURIN, a selective blocker of inosine 5'-phosphate dehydrogenase-activity. *Cancer Res* 49: 3696–3701

Tsukada T, Tomooka Y, Takai S, Ueda Y, Nishikawa S, Yagi T, Tokunaga T, Takeda N, Suda Y, Abe S *et al* (1993) Enhanced proliferative potential IN culture of cells from P53-deficient mice. *Oncogene* 8: 3313–3322

Tzoneva G, Dieck CL, Oshima K, Ambesi-Impiombato A, Sanchez-Martin M, Madubata CJ, Khiabanian H, Yu JY, Waanders E, Iacobucci I *et al* (2018) Clonal evolution mechanisms in NT5C2 mutant/relapsed acute lymphoblastic leukaemia. *Nature* 553: 511–514

Wang Z, Smith KS, Murphy M, Piloto O, Somervaille TCP, Cleary ML (2008) Glycogen synthase kinase 3 in MLL leukaemia maintenance and targeted therapy. *Nature* 455: 1205–1229

Wang YZ, Krivtsov AV, Sinha AU, North TE, Goessling W, Feng ZH, Zon LI, Armstrong SA (2010) The Wnt/beta-catenin pathway is required for the development of leukemia stem cells in AML. *Science* 327: 1650–1653

Wunderlich M, Brooks RA, Panchal R, Rhyasen GW, Danet-Desnoyers G, Mulloy JC (2014) OKT3 prevents xenogeneic GVHD and allows reliable xenograft initiation from unfractionated human hematopoietic tissues. *Blood* 123: E134–E144

Yang H, Fang ZH, Wei Y, Bohannan ZS, Ganan-Gomez I, Pierola AA, Paradiso LJ, Iwamura H, Garcia-Manero G (2017) Preclinical activity of FF-10501-01,

 ©2022 The Authors

a novel inosine-5'-monophosphate dehydrogenase inhibitor, in acute myeloid leukemia. *Leuk Res* 59: 85–92

Ye M, Zhang H, Yang H, Koche R, Staber PB, Cusan M, Levantini E, Welner RS, Bach CS, Zhang JY *et al* (2015) Hematopoietic differentiation is required for initiation of acute myeloid leukemia. *Cell Stem Cell* 17: 611–623

Yokoyama A (2015) Molecular mechanisms of MLL-associated leukemia. *Int J Hematol* 101: 352–361

Yokoyama A, Cleary ML (2008) Menin critically links MLL proteins with LEDGE on cancer-associated target genes. *Cancer Cell* 14: 36–46

Yokoyama A, Somervaille TCP, Smith KS, Rozenblatt-Rosen O, Meyerson M, Cleary ML (2005) The menin tumor suppressor protein is an essential oncogenic cofactor for MLL-associated leukemogenesis. *Cell* 123: 207–218

Zuber J, Rappaport AR, Luo WJ, Wang E, Chen C, Vaseva AV, Shi JW, Weissmueller S, Fellman C, Taylor MJ *et al* (2011a) An integrated approach to dissecting oncogene addiction implicates a Myb-coordinated self-renewal program as essential for leukemia maintenance. *Genes Dev* 25: 1628–1640

Zuber J, Shi JW, Wang E, Rappaport AR, Herrmann H, Sison EA, Magoon D, Qi J, Blatt K, Wunderlich M *et al* (2011b) RNAi screen identifies Brd4 as a therapeutic target in acute myeloid leukaemia. *Nature* 478: 524–528

License: This is an open access article under the terms of the Creative Commons Attribution License, which permits use, distribution and reproduction in any medium, provided the original work is properly cited.

Expanded View Figures

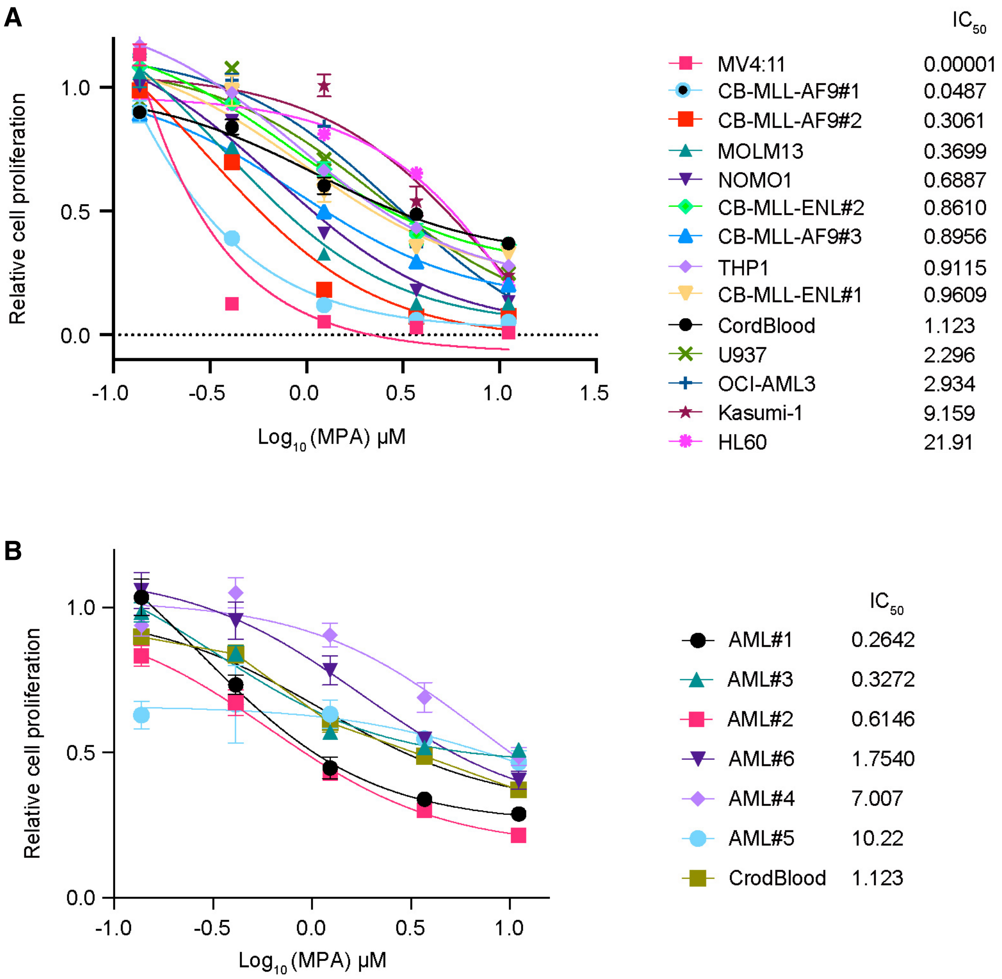

Figure EV1. The effect of IMPDH inhibitors on human myeloid leukemia cells *in vitro*.

A, B Cell viability assays were performed using WST-1. Human CB CD34^{+} cells and human AML cell lines (with MLL-fusions: MV4;11, MOLM13, NOMO1 and THP1, without MLL-fusions: HL60, Kasumi-1, OCI-AML3 and U937), CB cells expressing MLL-AF9 (CB-MA9#1, 2, 3) or MLL-ENL (CB-MLL-ENL#1, 2) (A) and PDX cell lines with or without MLL-fusions (AML#1, 2, 3 or AML#4, 5, 6) (B) were treated with titrating doses of MPA (1–100 µM) for 72 h in three technical replicates. IC50 values for each experiment are summarized in Fig 1B and C. Data are shown as mean ± SEM.

Source data are available online for this figure.

 ©2022 The Authors

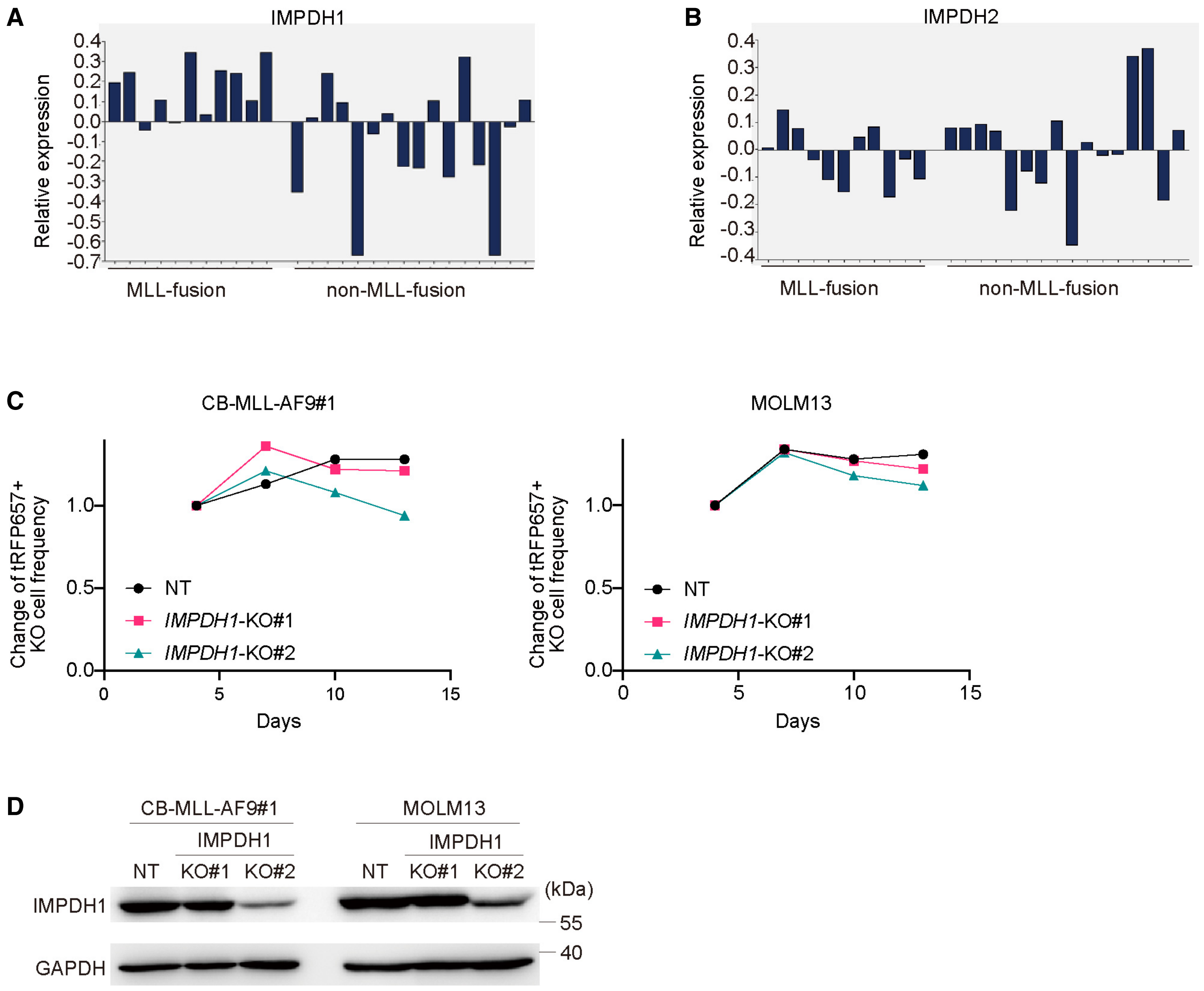

Figure EV2. Dependency of IMPDH1 and IMPDH2 in human AML cells.

A, B Relative mRNA expression of IMPDH1 (A) and IMPDH2 (B) in PDX cells derived from patients with MLL-fusion or non-MLL-fusion AMLs.

C MLL-AF9-expressing CB cells and MOLM13 cells were transduced with Cas9 together with non-targeting (NT) sgRNA or IMPDH1-targeting sgRNAs (IMPDH1-KO1 and KO2) co-expressing tRFP657. Changes in the frequency of $tRFP657^+$ cells (sgRNA-transduced cells) in cell cultures are shown. Results are normalized to the frequency of $tRFP657^+$ cells at day 4, set to 1.

D The $tRFP657^+$ cells were sorted and subjected to western blotting.

Source data are available online for this figure.

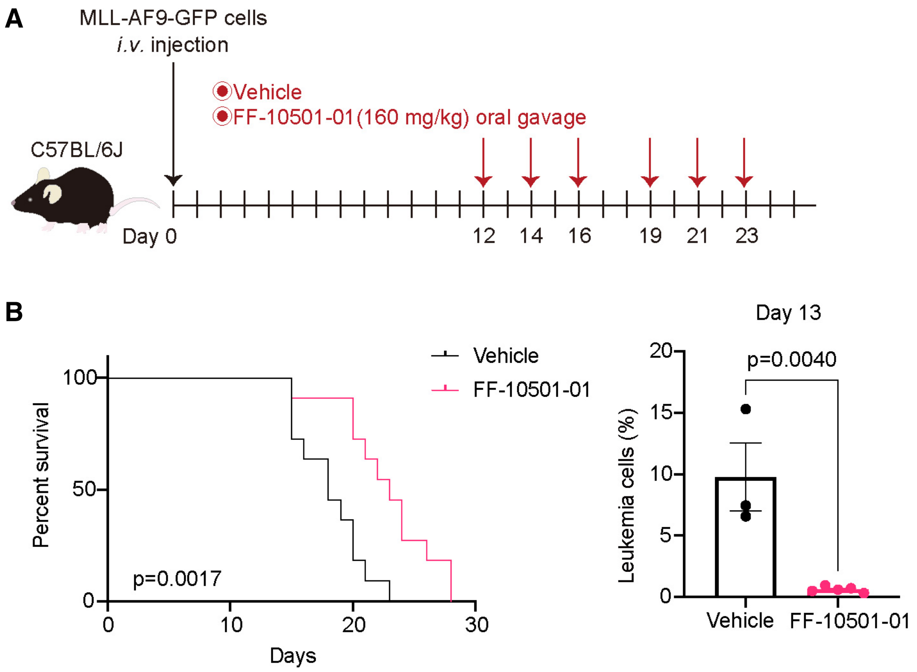

Figure EV3. Therapeutic effect of IMPDH inhibitor is significant on overt leukemia in mice.

A Experimental scheme used in (B). C57BL/6J mice were transplanted with mouse MLL-AF9-GFP cells and were treated with vehicle or FF-10501-01 from day 12 to 23, as indicated. GFP^{+} MLL-AF9 leukemia cells collected from vehicle- or FF-10501-01-treated mice were analyzed on day 13.

B (left) Kaplan–Meier survival curves of MLL-AF9 leukemia mice treated with vehicle or MMF ($n = 11$ per group). Statistical significance was evaluated by the log-rank test. (right) Frequencies of GFP^{+} leukemic cells in peripheral blood at day 13 are also shown (vehicle: $n = 3$, FF-10501-01: $n = 5$). A two-tailed unpaired t-test was used for the comparison. Data are shown as mean ± SEM.

Source data are available online for this figure.

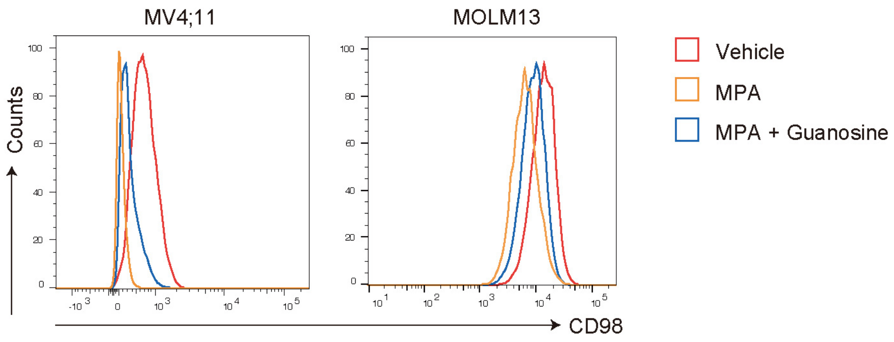

Figure EV4. IMPDH inhibition induces downregulation of CD98 in human AML cell lines *in vitro*.

FCM plots showing the CD98 expression in MV4:11 (left) and MOLM13 cells (right) after treated with/without 1 μM MPA and 100 μM Guanosine for 36 h.

Source data are available online for this figure.

©2022 The Authors
