# Supplementary material for: IMPDH inhibition activates TLR‐VCAM1 pathway and suppresses the development of MLL‐fusion leukemia
Source: EMBO Mol Med. 2022 Dec 1;15(1):e15631. doi: 10.15252/emmm.202115631 (PMC9832838; doi:10.15252/emmm.202115631)
Supplement: Supplementary file 8 [file EMMM-15-e15631-s007.pdf]

EMBO Molecular Medicine

IMPDH inhibition activates TLR-VCAM1 pathway and suppresses the development of MLL-fusion leukemia

Xiaoxiao Liu, Naru Sato, Tomohiro Yabushita, Jingmei Li, Yuhan Jia, Moe Tamura, Shuhei Asada, Takeshi Fujino, Tsuyoshi Fukushima, Taishi Yonezawa, Yosuke Tanaka, Tomofusa Fukuyama, Akiho Tsuchiya, Shiori Shikata, Hiroyuki Iwamura, Chieko Kinouchi, Kensuke Komatsu, Satoshi Yamasaki, Tatsuhiro Shibata, Atsuo Sasaki, Janet Schibler, Mark Wunderlich, Eric O'Brien, Benjamin Mizukawa, James Mulloy, Yuki Sugiura, Hitoshi Takizawa, Takuma Shibata, Kensuke Miyake, Toshio Kitamura, and Susumu Goyama
DOI: 10.15252/emmm.202115631

Corresponding author: Susumu Goyama (goyama@edu.k.u-tokyo.ac.jp)

Review Timeline:

Submission Date:	22nd Dec 21
Editorial Decision:	28th Jan 22
Revision Received:	30th Sep 22
Editorial Decision:	17th Oct 22
Revision Received:	8th Nov 22
Accepted:	8th Nov 22

Editor: Zeljko Durdevic

Transaction Report:

(Note: With the exception of the correction of typographical or spelling errors that could be a source of ambiguity, letters and reports are not edited. Depending on transfer agreements, referee reports obtained elsewhere may or may not be included in this compilation. Referee reports are anonymous unless the Referee chooses to sign their reports.)

28th Jan 2022

Dear Prof. Goyama,

Thank you for the submission of your manuscript to EMBO Molecular Medicine. We have now received feedback from the three reviewers who agreed to evaluate your manuscript. As you will see from the reports below, the referees acknowledge the interest of the study but also raise important and partially overlapping concerns that should be addressed in a major revision.

Further consideration of a revision that addresses reviewers' concerns in full will entail a second round of review. EMBO Molecular Medicine encourages a single round of revision only and therefore, acceptance or rejection of the manuscript will depend on the completeness of your responses included in the next, final version of the manuscript. For this reason, and to save you from any frustrations in the end, I would strongly advise against returning an incomplete revision.

We would welcome the submission of a revised version within six months for further consideration. Please let us know if you require longer to complete the revision.

Please use this link to login to the manuscript system and submit your revision: https://embomolmed.msubmit.net/cgi-bin/main.plex

I look forward to receiving your revised manuscript.

Yours sincerely,

Zeljko Durdevic

Zeljko Durdevic
Editor
EMBO Molecular Medicine

***** Reviewer's comments *****

Referee #1 (Comments on Novelty/Model System for Author):

It is a new therapeutic development for AML. They test both human and mouse model in vitro and in vivo.

Referee #1 (Remarks for Author):

This is an interesting and important study; the amount of work is enormous. The authors utilize innovative experimental and analytical methodologies to show the therapeutic effect of IMPDH inhibition on MLL-fusion acute myeloid leukemia (AML). Their results suggest that inhibition of IMPDH induces activation of TLR signaling and Vcam1 upregulation in MLL-fusion AML. Authors also tested combination treatment with TLR1/2 agonist and IMPDH inhibitor as a novel strategy to suppress the development of AML. The in vivo effect of IMPDH inhibitors on AML has not been fully investigated, therefore their study will add significant impact to the field. In general, it is a well-designed study and well-written manuscript, however, several additional experiments are needed to strengthen their conclusions and increase the impact of this study.

Major Comments:

1. The authors showed that AML cells with MLL-fusions are sensitive to IMPDH inhibitors in Figure 1. Whether MLL-fusion AMLs express higher levels of IMPDH1 and IMPDH2 than non-MLL-fusion AMLs? The expression levels of IMPDH1 and IMPDH2 in these AML cells should be examined. Are the levels of IMPDH1 and/or IMPDH2 corelates to the drug sensitivity?

2. In Figure 1H, the authors showed that genetic depletion of IMPDH2 inhibited the growth of MOLM13 and MLL-ENL-cord blood cells. Depletion of IMPDH2 needs to be confirmed by western blotting. In addition, the effect of IMPDH1 depletion should also be examined.

3. In Figure 2, the authors used only one PDX model (AML #1) to assess the effect of IMPDH inhibitors. The authors should perform this experiment using more PDX lines. Whether other AML cells are sensitive to IMPDH inhibition in the PDX models?

4. The authors showed that MPA enhanced Pam3CSK4-induced activation of TLR signaling only in THP1 cells in Figure 5F. These finding should be independently confirmed in this study, such as MLL-AF9-cord blood cells.

5. The authors showed that IMPDH inhibition increased Vcam1 expression in AML cells (Figure 6 and Figure 7). Does the guanosine supplementation reverse the Vcam1 upregulation induced by IMPDH inhibitors? Or they are independent with each other? It is also important to examine whether the Vcam1 upregulation is relevant with the induction of myeloid differentiation of AML cells.

6. Although the authors focused on MLL-fusion AML in this study, MLL rearrangements are also found frequently in acute lymphoid leukemia (ALL, both B and T-ALL). Could MLL-rearranged ALLs also be sensitive to IMPDH inhibitors?

Minor Comment:

1. Pam3CSK4 is a ligand for TLR1/2, but the authors used TLR7-expressed reporter cells in Figure 5B. Please confirm if it is correct or they share the same signaling pathway.

Referee #2 (Comments on Novelty/Model System for Author):

The data presented are innovative and provide some rationale for the clinical testing of MMF +- TLR agonists in MLL-fusion gene containing leukemias.

Referee #2 (Remarks for Author):

Liu and colleagues summarize an interesting set of experimental data suggesting that blockade of IMPDH by mycophenolic acid (MPA) leads to a susceptibility of MLL-fusion leukemia towards TLR-mediated cell death/apoptosis. The growth of various leukemia cell lines and hematopoietic cells were shown to be suppressed by an incubation with MMF preferentially when MLL-fusion genes were present of expressed in cord blood hematopoietic cells. Similar effects could be observed when targeting IMPDH1/2 by respective crispr/cas gene editing. The oral application of MMF in an alternating scheme lead to a prolonged survival of mice inoculated with the MLL-AF9 cell line and an inhibition of leukemic engraftment. Similar antileukemic effects could be observed in PDX models transplanted with human MLLL-AML cells. Biochemically, the incubation of leukemic cells with MPA induced anti-leukemic effects in a p53-independent manner. Combining MPa treatment with a TLR agonist had additive or even synergistic effects in-vitro and in-vivo and induces VCAM-1 expression. The data presented are innovative and provide some rationale for the clinical testing of MMF +- TLR agonists in MLL-fusion gene containing leukemias.
Overall, the manuscript is well written and the quality of the Figures and tables is adequate.

Major comments/suggestions:
The authors should consider and discuss that IMPDH activity may be increased in 15-20% of Caucasians with a 3757T>C polymorphic variant (rs11706052) and MPA would have less effects in such cases. Could the authors generate CB HSPC expressing this specific variant and test the effect of MPA in this setting?

What would be the clinical dosing regimen envisioned for phase I trials? Which combination partners would be advisable? With advent of new compounds would there by synergism with bcl-1 anatagonists and hyomethlyating agents?

Although IMPDH antagonism delays progression of leukemia, all animals succumb to disease only a few days later. What are the mechanisms of resistance, how could these be tackled?

Referee #3 (Remarks for Author):

The authors demonstrate the anti-leukemic activity of specific IMPDH inhibitors against MLL-fusion leukemia. Their findings may indicate that IMPDH inhibition induces TLR-TRAF6-NF-kB signaling resulting in AML cell differentiation.

This work is novel and potentially interesting. I do have several comments:

Major:
1.) IC50s of MPA should also be assessed in several non-MLL-fusion AML cell lines (including the FAB M4/M5 subtype of NPM1mut leukemias) to determine if these effects are specific for MLL-fusion leukemias.

2.) Primary AML cells are not easy to culture for longer than a couple of days. The authors should provide the information, which assay they used to determine IC50s and for how long those cells were cultured? Dose-response curves should be added to the supplement and information about the proliferation of the vehicle control-treated cells in these assays.

3.) For the experiments shown in Fig. 1E-H the authors use different MLL-fusion models (CB-MLL-AF9, MV411 cells, CB-MLL-ENL, MOLM13). The data from these different models should be consistently presented for all of these experiments.

4.) In the in vivo experiments presented in Fig.2 drug treatment was initiated on day 1 preventing engraftment of the leukemic cells. Do these drugs also have activity against overt leukemia in mice?

5.) The authors claim that CD98/Lat1 downregulation may contribute to the therapeutic effect of IMPDH inhibitors, while the expression of HOXA9 and MEIS1 is mainly unaffected (Fig. 4G, H) These findings are interesting and should be confirmed at least in some of the human AML cell lines as well.

6.) Why was the effect of MPA treatment on IkBa degradation and p38 phosphorylation induced by Pam3CSK4 only assessed in THP1 cells? As the authors claim that these inhibitors act at least in part via TLR-TRAF6-NF-kB signaling these findings should also be confirmed by assessing some of the other human MLL-r leukemia cell lines (e.g. MOLM13, MV411) and the CB-MLL-AF9 cells.

Minor:
1.) It is surprising to me that only 2 days of in vivo drug treatment induce substantial leukemia cell differentiation (Fig.2E+F) as differentiation induction with many other agents commonly need more time. After how many days can differentiation be observed in vitro?

Referee #1

We truly appreciate the insightful and constructive comments from the reviewer 1. The followings are point-by-point responses to the reviewer's comments and concerns.

Comment #1

The authors showed that AML cells with MLL-fusions are sensitive to IMPDH inhibitors in Figure 1. Whether MLL-fusion AMLs express higher levels of IMPDH1 and IMPDH2 than non-MLL-fusion AMLs? The expression levels of IMPDH1 and IMPDH2 in these AML cells should be examined. Are the levels of IMPDH1 and/or IMPDH2 corelates to the drug sensitivity?

Response to Comment #1

We assessed mRNA expression of IMPDH1 and IMPDH2 in human PDX cells with/without MLL-fusions and found no significant differences for the levels of IMPDH1/IMPDH2 between MLL-fusion AMLs and AMLs without MLL-fusions. Therefore, it appears that MLL-fusion leukemias are sensitive to IMPDH inhibitors not because they express high levels of IMPDH1/2. Instead, our data suggest that the active TLR signaling in MLL-fusion leukemia could explain why they are susceptible to IMPDH inhibition. We added the data of IMPDH1/IMPDH2 expression in PDX cells as **Figure EV2A** in the revised manuscript.

Comment #2

In Figure 1H, the authors showed that genetic depletion of IMPDH2 inhibited the growth of MOLM13 and MLL-ENL-cord blood cells. Depletion of IMPDH2 needs to be confirmed by western blotting. In addition, the effect of IMPDH1 depletion should also be examined.

Response to Comment #2

Thank you for this insightful suggestion. We confirmed efficient depletion of IMPDH2 in MOLM13 and MLL-ENL-expressing cord blood cells by western blotting. We also assessed the effect of IMPDH1 depletion in MOLM13 and MLL-AF9-expressing cord blood cells and found that IMPDH1 is dispensable for the growth of these MLL-fusion leukemia cells. These data suggest that IMPDH2 plays a critical role in promoting the development of MLL-fusion leukemia. We added these data (**Figure 1I and Figure EV2C, D**) to the revised manuscript.

Comment #3

In Figure 2, the authors used only one PDX model (AML #1) to assess the effect of IMPDH inhibitors. The authors should perform this experiment using more PDX lines. Whether other AML cells are sensitive to IMPDH inhibition in the PDX models?

Response to Comment #3

We assess the *in vivo* effect of MPA and FF-10501-01 using other PDX models (R/R AML harboring MLL-AF9, R/R AML harboring MLL-SEPT6 and AML harboring MLL-AF9 and NRAS mutation). Similar to the result of Figure 2, the IMPDH inhibitors did not show significant anti-leukemia effect in these PDX models (**Appendix Figure S5**), indicating that the importance of antitumor immunity to enhance the effect of IMPDH inhibitors against AML. We added the data to the revised manuscript.

Comment #4

The authors showed that MPA enhanced Pam3CSK4-induced activation of TLR signaling only in THP1 cells in Figure 5F. These finding should be independently confirmed in this study, such as MLL-AF9-cord blood cells.

Response to Comment #4

We performed a similar experiment using MLL-AF9-expressing cord blood cells and got the essentially same result to that obtained with THP1 cells. We added the data (**Figure 5G**) in the revised manuscript.

Comment #5

The authors showed that IMPDH inhibition increased Vcam1 expression in AML cells (Figure 6 and Figure 7). Does the guanosine supplementation reverse the Vcam1 upregulation induced by IMPDH inhibitors? Or they are independent with each other? It is also important to examine whether the Vcam1 upregulation is relevant with the induction of myeloid differentiation of AML cells.

Response to Comment #5

We appreciate this reviewer's comment. Guanosine supplementation partially reversed the Vcam1 upregulation (**Appendix Figure S10A**), indicating that Vcam1 upregulation in MLL-AF9 cells was induced by both direct and indirect (probably related to enhanced inflammation and myeloid differentiation induced by the IMPDH inhibitors) effects of IMPDH inhibition. We also assessed the effect of Vcam1 depletion on myeloid differentiation. Vcam1 depletion showed little effects on FF-10501-01-induced myeloid differentiation in MLL-AF9 cells (**Appendix Figure S10B**). Therefore, it appears that Vcam1 suppresses AML cell growth

mainly by inhibiting cell cycle progression (please see Figure 6F and H). We added these data and explanations to the revised manuscript.

Comment #6

Although the authors focused on MLL-fusion AML in this study, MLL rearrangements are also found frequently in acute lymphoid leukemia (ALL, both B and T-ALL). Could MLL-rearranged ALLs also be sensitive to IMPDH inhibitors?

Response to Comment #6

According to the reviewer's suggestion, we assessed the effect of MPA on CB cells expressing MLL-Af4 that recapitulate t (4;11) pro-B ALL. Addition of MPA induced a dramatic decrease in the formation of leukemic cobblestone-forming cells with a concomitant increase of $CD10^+$ expression in MLL-Af4 cells in a concentration-dependent manner (**Appendix Figure S4B, C**). Furthermore, MPA inhibited the cobblestone formation of PDX cells derived from patients with B-ALL with MLL-AF4 or MLL-AF9 from 1 μM of MPA. This effect was partially reversed by guanosine supplementation (**Appendix Figure S4D, E**). These data suggest that IMPDH inhibitors probably have growth-inhibitory effects on ALLs with MLL-fusions, which warrants further investigation. We added these data and explanations to the revised manuscript.

Comment #7

Pam3CSK4 is a ligand for TLR1/2, but the authors used TLR7-expressed reporter cells in Figure 5B. Please confirm if it is correct or they share the same signaling pathway.

Response to Comment #7

Thank you for pointing it out. Because TLR1 and TLR2 are highly expressed in Ba/F3 cells, we used original BaκB cells for the experiment. We corrected the corresponding label in **Figure 5B**.

Referee #2

We truly appreciate the insightful and constructive comments from the Referee #2. The followings are point-by-point responses to the reviewer's comments and concerns.

Comment #1

The authors should consider and discuss that IMPDH activity may be increased in 15 -20% of Caucasians with a 3757T>C polymorphic variant (rs11706052) and MPA would

have less effects in such cases. Could the authors generate CB HSPC expressing this specific variant and test the effect of MPA in this setting?

Response to Comment #1

Whether hematopoietic or leukemic cells with the 3757T>C variant are less sensitive to MPA is definitely an interesting question. Unfortunately, it is still technically challenging to knock-in specific variants in cord blood cells. (We have established the experimental system to knockout individual genes in cord blood cells, but "knock-in" is far more difficult than "knock-out".) We would like to try this experiment in the next project, but it is beyond the scope of this paper.

Comment #2

What would be the clinical dosing regimen envisioned for phase I trials? Which combination partners would be advisable? With advent of new compounds would there by synergism with bcl-1 antagonists and hypomethylating agents?

Response to Comment #2

We appreciate this insightful comment. In the Phase 1/2a study, patients with relapsed/refractory AML or MDS received FF-10501-01 oral doses 50-500 mg/m2 twice daily for 14 or 21 days out of each 28-day cycle. Although FF-10501-01 demonstrated clinical activity and target inhibition in these patients, it also increased mucositis events with this treatment schedule, which led to Phase 2a closure [Leuk Lymphoma 61(8):1943-1953, 2020. doi: 10.1080/10428194.2020.1747065]. In this study, we showed that alternate-day administration of MPA and FF-10501-01 to mice effectively suppressed MLL-AF9-driven leukemogenesis without having devastating side effects. The therapeutic effect of the alternate-day administration of IMPDH inhibitors warrants further investigation in clinical trials.

For the combination therapy, we previously showed that azacitidine-resistant leukemia cell lines were still sensitive to IMPDH inhibitors [Pharmacol Res Perspect. 4(1): e00206, 2016. doi: 10.1002/prp2.206), indicating that IMPDH inhibition could be an alternative treatment for AML/MDS patients with acquired resistance to hypomethylating agents (azacitidine or decitabine). Therefore, we first assessed the drug synergism between MPA and decitabine against human CB cells expressing MLL-AF9. Although both drugs inhibited the growth of MLL-AF9 cells, we did not observe the synergistic antileukemia effect between them. Interestingly, we found that MPA synergized with the BCL2 inhibitor (Venetoclax) to inhibit the growth of MLL-AF9 cells, indicating that combined treatment with IMPDH and BCL2 inhibitors could be promising frontline therapies for MLL-fusion leukemia. We added these

data (**Appendix Figure S3A, B**) and explanations to the revised manuscript.

Comment #3

Although IMPDH antagonism delays progression of leukemia, all animals succumb to disease only a few days later. What are the mechanisms of resistance, how could these be tackled?

Response to Comment #3

As the referee #2 pointed out, IMPDH inhibitors could not eradicate all MLL-fusion AML cells *in vivo*. Co-treatment with IMPDH inhibitors and TLR1/2 agonist (Pam3CSK4) showed the stronger anti-leukemia effect, but this combination therapy was still not sufficient to cure the MLL-fusion AML. Thus, Mechanisms of resistance to IMPDH inhibition remain to be elucidated in future studies. We are currently planning to perform CRISPR/Cas9 library screening to identify key molecules associated with the resistance to IMPDH inhibitors, but it is beyond the scope of this paper.

Referee #3

We truly appreciate the insightful and constructive comments from the Referee #3. The followings are point-by-point responses to the reviewer's comments and concerns.

Comment #1

IC50s of MPA should also be assessed in several non-MLL-fusion AML cell lines (including the FAB M4/M5 subtype of NPM1mut leukemias) to determine if these effects are specific for MLL-fusion leukemias.

Response to Comment #1

According to this comment, we assessed the effect of MPA on four additional AML cell lines without MLL-fusions, HL60, Kasumi1, OCI-AML3 and U937, and found that all these four cell lines are relatively resistant to MPA. These data support our conclusion that MLL-fusion leukemias are particularly sensitive to IMPDH inhibitors. We added the data (**Figure 1B**, **Figure EV1A and Appendix Figure S1D**) to the revised manuscript.

Comment #2

Primary AML cells are not easy to culture for longer than a couple of days. The authors should provide the information, which assay they used to determine IC50s and for how long those cells were cultured? Dose-response curves should be added to the

supplement and information about the proliferation of the vehicle control-treated cells in these assays.

Response to Comment #2

We cultured the PDX-derived AML cells in IMDM containing 20% BIT9500 or StemSpan SFEM II medium with 10 ng/mL SCF, TPO, Flt-3 ligand, IL-3 and IL-6. In this culture condition, the PDX cells grow well for at least several weeks. They were cultured with titrating doses of MPA or FF-10501-01 together with or without 100 μM Guanosine. The growth of the cells was assessed by WST-8 assay after 72 hours of culture. Dose-response curves for the PDX cells were shown in **Figure EV1 and Appendix Figure S1**.

Comment #3

For the experiments shown in Fig. 1E-H, the authors use different MLL-fusion models (CB-MLL-AF9, MV411 cells, CB-MLL-ENL, MOLM13). The data from these different models should be consistently presented for all of these experiments.

Response to Comment #3

We appreciate this reviewer's comment. To validate the findings, we assessed the effect of MPA on MOLM13 cells and confirmed that MPA induced cell cycle arrest and apoptosis in MOLM13 cells. We also confirmed that the effect of MPA on MOLM13 cells was reversed, at least partially, by the guanosine supplementation. We added the data (**Appendix Figure S2**) to the revised manuscript.

Comment #4

In the in vivo experiments presented in Fig.2 drug treatment was initiated on day 1 preventing engraftment of the leukemic cells. Do these drugs also have activity against overt leukemia in mice?

Response to Comment #4

To address this question, we transplanted MLL-AF9 cells into mice and started treatment on day12. This delayed start of FF-10501-01 treatment also inhibited the development of MLL-AF9-induced AML. We added the data (**Figure EV3**) to the revised manuscript.

Comment #5

The authors claim that CD98/Lat1 downregulation may contribute to the therapeutic effect of IMPDH inhibitors, while the expression of HOXA9 and MEIS1 is mainly

unaffected (Fig. 4G, H) These findings are interesting and should be confirmed at least in some of the human AML cell lines as well.

Response to Comment #5

To address this question, we examined if MPA treatment downregulates CD98 expression in MOLM13 cells and MV4;11 cells. As shown in **Figure EV4**, MPA induced CD98 downregulation in both cell lines, which was partially reversed by the guanosine supplementation. These data suggest that CD98 downregulation is a direct consequence of IMPDH inhibition. We added the data to the revised manuscript.

Comment #6

Why was the effect of MPA treatment on IkBa degradation and p38 phosphorylation induced by Pam3CSK4 only assessed in THP1 cells? As the authors claim that these inhibitors act at least in part via TLR-TRAF6-NF-kB signaling these findings should also be confirmed by assessing some of the other human MLL-r leukemia cell lines (e.g. MOLM13, MV411) and the CB-MLL-AF9 cells.

Response to Comment #6

We assessed the activity of TLR signaling upon Pam3CSK4 treatment in CB-MLL-AF9 cells and got essentially the same results to those obtained in THP1 cells. We added the data (**Figure 5G**) to the revised manuscript.

Comment #7

It is surprising to me that only 2 days of in vivo drug treatment induce substantial leukemia cell differentiation (Fig.2E+F) as differentiation induction with many other agents commonly need more time. After how many days can differentiation be observed in vitro?

Response to Comment #7

Yes, IMPDH inhibition induced differentiation of mouse MLL-AF9 cells *in vivo* only in 2 days. IMPDH inhibition also induced differentiation of human MLL-AF9 cells *in vitro* in 3-4 days. These data indicate that IMPDH inhibition suppresses MLL-fusion-induced leukemogenesis mainly by inducing myeloid differentiation.

17th Oct 2022

Dear Prof. Goyama,

Thank you for the submission of your revised manuscript to EMBO Molecular Medicine. I am pleased to inform you that we will be able to accept your manuscript pending the following final amendments:

1) Authors: E-mail correspondence to Kensuke Komatsu, Takeshi Fujino, Moe Tamura could not be delivered. Please update their e-mail addresses and make sure to enter correct e-mail addresses for all authors in our submission system.
2) In the main manuscript file, please do the following:
- Correct/answer the track changes suggested by our data editors by working from the attached document.
- Remove text highlight colour.
- Correct callout for Supplementary Table 4 to Appendix Table 4.
- In M&M, in addition to the statement about the informed consent and the WMA Declaration of Helsinki confirm that the experiments also conformed to the principles set out in the Department of Health and Human Services Belmont Report.
- In M&M, statistical paragraph should reflect all information that you have filled in the Authors Checklist, especially regarding randomization, blinding, replication.
- Indicate in legends exact n= and exact p= values, not a range, along with the statistical test used. To keep the figures "clear" some authors found providing an Appendix table Sx with all exact p-values preferable. You are welcome to do this if you want to.
- Please rename "Competing Interest" to "Disclosure Statement & Competing Interests" and move it after the "Acknowledgements". We updated our journal's competing interests policy in January 2022 and request authors to consider both actual and perceived competing interests. Please review the policy https://www.embopress.org/competing-interests and update your competing interests if necessary.
- Rename "Data and material availability" to "Data availability" and move it to the end of M&M section. We noticed that deposited Mass Cytometry data are not accessible, please be aware that all datasets should be made freely available upon acceptance, without restriction.
- Correct the reference citation in the text and reference list. In the text, a reference should be cited by author and year of publication. Include a space between a word and the opening parenthesis of the reference that follows. In the reference list, citations should be listed in alphabetical order. Where there are more than 10 authors on a paper, 10 will be listed, followed by "et al.". Please check "Author Guidelines" for more information.
https://www.embopress.org/page/journal/17574684/authorguide#referencesformat
3) Funding: Add complete project numbers in our submission system. Please make sure that information about all sources of funding are complete in both our submission system and in the manuscript.
4) Appendix: All appendix files should be compiled in one PDF with a table of contents on the title page and figure legends blow the figures. Please provide the antibody dilutions that were used for each antibody in corresponding Appendix tables.
5) Author contributions: Please remove it from the manuscript and specify author contributions in our submission system. CRediT has replaced the traditional author contributions section because it offers a systematic machine-readable author contributions format that allows for more effective research assessment. You are encouraged to use the free text boxes beneath each contributing author's name to add specific details on the author's contribution. More information is available in our guide to authors:
https://www.embopress.org/page/journal/17574684/authorguide#authorshipguidelines
6) Synopsis: Please check your synopsis text and image and submit their final versions with your revised manuscript. Please be aware that in the proof stage minor corrections only are allowed (e.g., typos).
7) For more information: Please remove data availability information. This space should be used to list relevant web links for further consultation by our readers. Could you identify some relevant ones and provide such information as well? Some examples are patient associations, relevant databases, OMIM/proteins/genes links, author's websites, etc...
8) Source data: We encourage you to include the source data for figure panels that show essential data. Numerical data should be provided as individual .xls or .csv files (including a tab describing the data). For blots or microscopy, uncropped images should be submitted (using a zip archive if multiple images need to be supplied for one panel). Please check "Author Guidelines" for more information. https://www.embopress.org/page/journal/17574684/authorguide#sourcedata
9) As part of the EMBO Publications transparent editorial process initiative (see our Editorial at http://embomolmed.embopress.org/content/2/9/329), EMBO Molecular Medicine will publish online a Review Process File (RPF) to accompany accepted manuscripts. This file will be published in conjunction with your paper and will include the anonymous referee reports, your point-by-point response and all pertinent correspondence relating to the manuscript. Let us know whether you agree with the publication of the RPF and as here, if you want to remove or not any figures from it prior to publication. Please note that the Authors checklist will be published at the end of the RPF.
10) Please provide a point-by-point letter INCLUDING my comments as well as the reviewer's reports and your detailed responses (as Word file).

I look forward to reading a new revised version of your manuscript as soon as possible.

Yours sincerely,

Zeljko Durdevic

Zeljko Durdevic
Editor
EMBO Molecular Medicine

***** Reviewer's comments *****

Referee #1 (Comments on Novelty/Model System for Author):

It is a highly innovative study with potential of huge clinical impacts.

Referee #1 (Remarks for Author):

All my concerns and other reviewers' concerns have been addressed sufficiently. It is a very nice paper as it is.

Referee #2 (Remarks for Author):

Comments have been adressed adequately

The authors addressed the editorial issues.

2nd Revision - Editorial Decision 8th Nov 2022

We are pleased to inform you that your manuscript is accepted for publication and is now being sent to our publisher to be included in the next available issue of EMBO Molecular Medicine.

EMBO Press Author Checklist

Corresponding Author Name: Susumu Goyama	
Journal Submitted to: EMBO Molecular Medicine	
Manuscript Number: EMM-2021-15631	

USEFUL LINKS FOR COMPLETING THIS FORM
The EMBO Journal - Author Guidelines
EMBO Reports - Author Guidelines
Molecular Systems Biology - Author Guidelines
EMBO Molecular Medicine - Author Guidelines

Reporting Checklist for Life Science Articles (updated
This checklist is adapted from Materials Design Analysis Reporting (MDAR) Checklist for Authors. MDAR establishes a minimum set of requirements in transparent reporting in the life sciences (see Statement of Task: 10.31222/osf.io/9sm4x). Please follow the journal's guidelines in
Please note that a copy of this checklist will be published alongside your article.

Abridged guidelines for figures
1. Data
The data shown in figures should satisfy the following conditions:
- the data were obtained and processed according to the field's best practice and are presented to reflect the results of the experiments in an accurate and unbiased manner.
- ideally, figure panels should include only measurements that are directly comparable to each other and obtained with the same assay.
- plots include clearly labeled error bars for independent experiments and sample sizes. Unless justified, error bars should not be shown for
- if n<5, the individual data points from each experiment should be plotted. Any statistical test employed should be justified.
- Source Data should be included to report the data underlying figures according to the guidelines set out in the authorship guidelines on

2. Captions
Each figure caption should contain the following information, for each panel where they are relevant:
- a specification of the experimental system investigated (eg cell line, species name).
- the assay(s) and method(s) used to carry out the reported observations and measurements.
- an explicit mention of the biological and chemical entity(ies) that are being measured.
- an explicit mention of the biological and chemical entity(ies) that are altered/varied/perturbed in a controlled manner.
- the exact sample size (n) for each experimental group/condition, given as a number, not a range;
- a description of the sample collection allowing the reader to understand whether the samples represent technical or biological replicates (including how many animals, litters, cultures, etc.).
- a statement of how many times the experiment shown was independently replicated in the laboratory.
- definitions of statistical methods and measures:

- common tests, such as t-test (please specify whether paired vs. unpaired), simple χ2 tests, Wilcoxon and Mann-Whitney tests, can be unambiguously identified by name only, but more complex techniques should be described in the methods section;

- are tests one-sided or two-sided?
- are there adjustments for multiple comparisons?
- exact statistical test results, e.g., P values = x but not P values < x;
- definition of 'center values' as median or average;
- definition of error bars as s.d. or s.e.m.

Please complete ALL of the questions below.
Select "Not Applicable" only when the requested information is not relevant for your study.

Materials

Newly Created Materials	Information included in the manuscript?	In which section is the information available? (Reagents and Tools Table, Materials and Methods, Figures, Data Availability Section)
New materials and reagents need to be available; do any restrictions apply?	Not Applicable	

Antibodies	Information included in the manuscript?	In which section is the information available? (Reagents and Tools Table, Materials and Methods, Figures, Data Availability Section)
For **antibodies** provide the following information:<br>- Commercial antibodies: RRID (if possible) or supplier name, catalogue number and or/clone number<br>- Non-commercial: RRID or citation	Yes	Reagents Table in Appendix

DNA and RNA sequences	Information included in the manuscript?	In which section is the information available? (Reagents and Tools Table, Materials and Methods, Figures, Data Availability Section)
**Short novel DNA or RNA including primers, probes**: provide the sequences.	Yes	Methods section

Cell materials	Information included in the manuscript?	In which section is the information available? (Reagents and Tools Table, Materials and Methods, Figures, Data Availability Section)
**Cell lines:** Provide species information, strain. Provide accession number in repository **OR** supplier name, catalog number, clone number, and/**OR** RRID.	Yes	Materials and Methods
**Primary cultures:** Provide species, strain, sex of origin, genetic modification status.	Yes	Materials and Methods and Appendix
Report if the cell lines were recently **authenticated** (e.g., by STR profiling) and tested for mycoplasma contamination.	Not Applicable	

Experimental animals	Information included in the manuscript?	In which section is the information available? (Reagents and Tools Table, Materials and Methods, Figures, Data Availability Section)
**Laboratory animals or Model organisms:** Provide species, strain, sex, age, genetic modification status. Provide accession number in repository **OR** supplier name, catalog number, clone number, **OR** RRID.	Yes	Materials and Methods
**Animal observed in or captured from the field:** Provide species, sex, and age where possible**.**	Not Applicable	
Please detail **housing and husbandry conditions**.	Yes	Materials and Methods

Plants and microbes	Information included in the manuscript?	In which section is the information available? (Reagents and Tools Table, Materials and Methods, Figures, Data Availability Section)
**Plants:** provide species and strain, ecotype and cultivar where relevant, unique accession number if available, and source (including location for collected wild specimens).	Not Applicable	
**Microbes:** provide species and strain, unique accession number if available, and source.	Not Applicable	

Human research participants	Information included in the manuscript?	In which section is the information available? (Reagents and Tools Table, Materials and Methods, Figures, Data Availability Section)
If collected and within the bounds of privacy constraints report on age, sex and gender or ethnicity for all study participants.	Not Applicable	

Core facilities	Information included in the manuscript?	In which section is the information available? (Reagents and Tools Table, Materials and Methods, Figures, Data Availability Section)

If your work benefited from core facilities, was their service mentioned in the acknowledgments section?	Yes	acknowledgments

Design

**Study protocol**	**Information included in the manuscript?**	**In which section is the information available?** (Reagents and Tools Table, Materials and Methods, Figures, Data Availability Section)
If study protocol has been **pre-registered, provide DOI in the manuscript**. For clinical trials, provide the trial registration number **OR** cite DOI.	Not Applicable	
Report the **clinical trial registration number** (at ClinicalTrials.gov or equivalent), where applicable.	Not Applicable	

**Laboratory protocol**	**Information included in the manuscript?**	**In which section is the information available?** (Reagents and Tools Table, Materials and Methods, Figures, Data Availability Section)
Provide DOI OR other citation details if **external detailed step-by-step protocols** are available.	Not Applicable	

**Experimental study design and statistics**	**Information included in the manuscript?**	**In which section is the information available?** (Reagents and Tools Table, Materials and Methods, Figures, Data Availability Section)
Include a statement about **sample size** estimate even if no statistical methods were used.	Yes	Materials and Methods
Were any steps taken to minimize the effects of subjective bias when allocating animals/samples to treatment (e.g. **randomization procedure**)? If yes, have they been described?	Not Applicable	
Include a statement about **blinding** even if no blinding was done.	Yes	Materials and Methods
Describe **inclusion/exclusion criteria** if samples or animals were excluded from the analysis. Were the criteria pre-established?<br><br>If sample or data points were omitted from analysis, report if this was due to attrition or intentional exclusion and provide justification.	Not Applicable	
For every figure, are **statistical tests** justified as appropriate? Do the data meet the assumptions of the tests (e.g., normal distribution)? Describe any methods used to assess it. Is there an estimate of variation within each group of data? Is the variance similar between the groups that are being statistically compared?	Yes	Materials and Methods, Figures.

**Sample definition and in-laboratory replication**	**Information included in the manuscript?**	**In which section is the information available?** (Reagents and Tools Table, Materials and Methods, Figures, Data Availability Section)
In the figure legends: state number of times the experiment was **replicated** in laboratory.	Yes	Figure legends
In the figure legends: define whether data describe **technical or biological replicates**.	Yes	Figure legends

Ethics

**Ethics**	**Information included in the manuscript?**	**In which section is the information available?** (Reagents and Tools Table, Materials and Methods, Figures, Data Availability Section)
Studies involving **human participants**: State details of **authority granting ethics approval** (IRB or equivalent committee(s), provide	Yes	Materials and Methods
Studies involving **human participants**: Include a statement confirming that **informed consent** was obtained from all subjects	Yes	Materials and Methods
Studies involving **human participants:** For publication of **patient photos**, include a statement confirming that consent to publish was obtained.	Not Applicable	
Studies involving experimental **animals**: State details of **authority granting ethics approva**l (IRB or equivalent committee(s), provide reference number for approval. Include a statement of compliance with ethical regulations.	Yes	Materials and Methods
Studies involving **specimen and field samples:** State if relevant **permits** obtained, provide details of authority approving study; if none were required, explain why.	Not Applicable	

**Dual Use Research of Concern (DURC)**	**Information included in the manuscript?**	**In which section is the information available?** (Reagents and Tools Table, Materials and Methods, Figures, Data Availability Section)
Could your study fall under dual use research restrictions? Please check biosecurity documents and list of **select agents and toxins** (CDC): https://www.selectagents.gov/sat/list.htm	Not Applicable	
If you used a select agent, is the security level of the lab appropriate and reported in the manuscript?	Not Applicable	
If a study is subject to dual use research of concern regulations, is the name of the **authority granting approval and reference number** for the regulatory approval provided in the manuscript?	Not Applicable	

Reporting

The MDAR framework recommends adoption of discipline-specific guidelines, established and endorsed through community initiatives. Journals have their own policy about requiring specific guidelines and recommendations to complement MDAR.

**Adherence to community standards**	**Information included in the manuscript?**	**In which section is the information available?** (Reagents and Tools Table, Materials and Methods, Figures, Data Availability Section)
State if relevant guidelines or checklists (e.g., **ICMJE, MIBBI, ARRIVE, PRISMA**) have been followed or provided.	Not Applicable	
For **tumor marker prognostic studies,** we recommend that you follow the **REMARK** reporting guidelines (see link list at top right)	Not Applicable	
For **phase II and III randomized controlled trials**, please refer to the **CONSORT** flow diagram (see link list at top right) and submit the CONSORT checklist (see link list at top right) with your submission. See author guidelines, under 'Reporting Guidelines'. Please confirm you have submitted this list.	Not Applicable	

Data Availability

**Data availability**	**Information included in the manuscript?**	**In which section is the information available?** (Reagents and Tools Table, Materials and Methods, Figures, Data Availability Section)
Have **primary datasets** been deposited according to the journal's guidelines (see 'Data Deposition' section) and the respective accession numbers provided in the Data Availability Section?	Yes	Data Availability Section
Were **human clinical and genomic datasets** deposited in a public access-controlled repository in accordance to ethical obligations to the patients and to the applicable consent agreement?	Not Applicable	
Are **computational models** that are central and integral to a study available without restrictions in a machine-readable form? Were the relevant accession numbers or links provided?	Not Applicable	
If publicly available data were reused, provide the respective **data citations in the reference list**.	Yes	Materials and Methods, Figure legends
